# Exosomal Transport of Hepatocyte‐Derived Drug‐Modified Proteins to the Immune System

**DOI:** 10.1002/hep.30701

**Published:** 2019-06-29

**Authors:** Monday O. Ogese, Rosalind E. Jenkins, Kareena Adair, Arun Tailor, Xiaoli Meng, Lee Faulkner, Bright O. Enyindah, Amy Schofield, Rafael Diaz‐Nieto, Lorenzo Ressel, Gina L. Eagle, Neil R. Kitteringham, Chris E. Goldring, B. Kevin Park, Dean J. Naisbitt, Catherine Betts

**Affiliations:** ^1^ New Modality Safety, Clinical Pharmacology and Safety Sciences, R&D Biopharmaceuticals AstraZeneca Cambridge United Kingdom; ^2^ MRC Centre for Drug Safety Science, Department of Molecular and Clinical Pharmacology University of Liverpool Liverpool United Kingdom; ^3^ Department of Veterinary Pathology and Public Health, Institute of Veterinary Science University of Liverpool, Leahurst Campus Neston United Kingdom; ^4^ North Western Hepatobiliary Unit Aintree University Hospital NHS Foundation Trust Liverpool United Kingdom

## Abstract

Idiosyncratic drug‐induced liver injury (DILI) is a rare, often difficult‐to‐predict adverse reaction with complex pathomechanisms. However, it is now evident that certain forms of DILI are immune‐mediated and may involve the activation of drug‐specific T cells. Exosomes are cell‐derived vesicles that carry RNA, lipids, and protein cargo from their cell of origin to distant cells, and they may play a role in immune activation. Herein, primary human hepatocytes were treated with drugs associated with a high incidence of DILI (flucloxacillin, amoxicillin, isoniazid, and nitroso‐sulfamethoxazole) to characterize the proteins packaged within exosomes that are subsequently transported to dendritic cells for processing. Exosomes measured between 50 and 100 nm and expressed enriched CD63. Liquid chromatography–tandem mass spectrometry (LC/MS‐MS) identified 2,109 proteins, with 608 proteins being quantified across all exosome samples. Data are available through ProteomeXchange with identifier PXD010760. Analysis of gene ontologies revealed that exosomes mirrored whole human liver tissue in terms of the families of proteins present, regardless of drug treatment. However, exosomes from nitroso‐sulfamethoxazole‐treated hepatocytes selectively packaged a specific subset of proteins. LC/MS‐MS also revealed the presence of hepatocyte‐derived exosomal proteins covalently modified with amoxicillin, flucloxacillin, and nitroso‐sulfamethoxazole. Uptake of exosomes by monocyte‐derived dendritic cells occurred silently, mainly through phagocytosis, and was inhibited by latrunculin A. An amoxicillin‐modified 9‐mer peptide derived from the exosomal transcription factor protein SRY (sex determining region Y)‐box 30 activated naïve T cells from human leukocyte antigen A*02:01–positive human donors. *Conclusion:* This study shows that exosomes have the potential to transmit drug‐specific hepatocyte‐derived signals to the immune system and provide a pathway for the induction of drug hapten‐specific T‐cell responses.

**Abbreviations**
ACN: Acetonitrile
ANOVA: analysis of variance
DILI: drug‐induced liver injury
HLA: human leukocyte antigen
HSP: heat shock protein
IL: interleukin
LC/MS‐MS: liquid chromatography–tandem mass spectrometry
MHC: major histocompatibility complex
PBMC: peripheral blood mononuclear cells
SOX30: SRY (sex determining region Y)‐box 30
TFA: trifluoroacetic acid

Drug‐induced liver injury (DILI) is a complex, multistep, and sometimes fatal adverse drug reaction.^1^ Whereas type A reactions can be explained by the pharmacology of the drug, the molecular mechanisms of type B or idiosyncratic reactions remain the focus of intensive research. Idiosyncratic DILI is rare and difficult to predict; hence, it is reasonable to assume that these reactions are associated with specific patient risk factors. Genome‐wide association studies have linked specific human leukocyte antigens (HLAs) to DILI, and as HLA molecules present antigenic determinants to T cells, the genetic studies implicate the adaptive immune system in the disease pathogenesis. Adverse reactions to amoxicillin clavulanate,^2^ flucloxacillin,^3^ lapatinib,^4^ lumiracoxib,^5^ minocycline,^6^ ticlopidine,^7^ and ximelagatran^8^ are all associated with a specific risk allele. Although the exact role of drug‐specific T cells in DILI is not fully understood, recent studies have detected drug‐specific T cells in (1) the peripheral blood^9^, ^10^, ^11^ and (2) liver biopsies from patients with DILI.^12^, ^13^ Similarly, T cells have also been shown to induce cytotoxicity of hepatocyte‐like cells transfected with the risk allele HLA‐B*57:01.^13^ It is often assumed that antigenic and stress‐related signals from the liver are important for adaptive immune stimulation in patients with DILI; however, the origin and mechanism of transmission of these signals are difficult to define after systemic drug exposure.^14^ Because primary human hepatocytes are the principle target for DILI drugs, we hypothesize that they transmit drug‐specific or at least drug‐dependent signals to the immune system.^15^ Hence, the purpose of this study was to characterize the proteins encapsulated within exosomes derived from hepatocytes treated with DILI drugs and to determine whether exosomes deliver drug‐specific signals to dendritic cells that subsequently activate the adaptive immune system.

Exosomes are membrane‐bound nano vesicles that originate from the endosomal compartment. They are either degraded by lysosomes or secreted into the extracellular space upon fusion with the plasma membrane.^16^ The biogenesis, cargo sorting, and ubiquitin‐dependent degradation of exosomes are regulated by a combination of Endosomal sorting complexes required for transport (ESCRTs) and multiprotein complexes.^17^, ^18^ These vesicles transport functional macromolecular components from their cells of origin to distant cells and are thought to play an important role in intercellular communication.^19^ Although the biogenesis of exosomes is conserved in eukaryotes, Kruger et al. demonstrated significant differences in proteomic and miRNA profiles of exosomes derived from Michigan Cancer Foundation 7 and MDA‐MB 231 cells.^20^ In addition, the composition of exosomes can be regulated by factors like infection, stress, and disease.^21^, ^22^ Therefore, it is plausible that the sorting and packaging of hepatocyte‐derived exosomes may be influenced by drug exposure, disease state, and/or the unique phenotype of patients with drug‐induced liver injury.

Interestingly, tumor‐derived exosomes transport multiple membrane‐bound and soluble factors that suppress the function of human cytotoxic CD4^+^ and CD8^+^ T cells.^23^ Administration of the immunotherapeutic agent IRX‐2 was shown to protect CD8^+^ T cells from tumor‐derived exosome‐induced apoptosis, resulting in enhanced T‐cell‐mediated antitumor activity.^24^ Paradoxically, the ability of exosomes to cross the cell membrane has also been explored for targeted drug delivery systems in cancer immunotherapy.^25^

A dogma of drug immunogenicity research is that the binding of drugs to cellular proteins induces a tissue‐specific signature to direct the immune response. In this respect, exosomes have the potential to transport drug‐modified proteins from hepatocytes to dendritic cells for protein processing and ultimately the display of antigenic peptides. Thus, this study aimed to (1) characterize protein profiles of hepatocyte‐derived exosomes and identify specific drug modifications and (2) explore the cellular uptake of hepatocyte‐derived exosomes and the impact of this process on dendritic cell function and T‐cell activation.

## Materials and Methods

### Isolation of Hepatocytes From Human Liver Tissue

Liver biopsies from consenting donors were used for primary human hepatocyte isolation. Liver biopsies were obtained from consented donors following hepatic resections of varying aetiologies conducted at the University of Liverpool teaching hospital, Aintree. All donors provided written informed consent to partake in the study approved by the Liverpool local research ethics committee. Biopsies were first perfused with 4‐(2‐hydroxyethyl)‐1‐piperazine ethanesulfonic acid buffer for 20‐30 minutes. This was followed by tissue digest using collagenase type IV (Sigma Aldrich, United Kingdom). Hepatocytes were harvested using Williams E medium and washed twice by gradient centrifugation for 5 minutes, at 4°C and 80*g*. Hepatocytes were cultured in Williams E supplemented with L‐glutamine (2 mM), penicillin (100 µg/mL), streptomycin (100 U/mL), insulin‐transferrin‐selenium (100×), and dexamethasone (1 µM/mL) using 6‐well plates precoated with collagen‐I (Corning Flintshire, United Kingdom). Cells were then maintained in fresh culture media overnight before exposure to test drugs for 24 hours.

### Drug Hepatocyte Treatment and Isolation of Exosomes From Culture Supernatant

Culture supernatant from hepatocytes treated with subtoxic concentrations of amoxicillin (0.05 mM), flucloxacillin (0.05 mM), isoniazid (0.03 mM), and nitroso‐sulfamethoxazole (0.01 mM)^26^ was collected for exosome isolation after 24 hours and cells were lysed in radio immunoprecipitation assay (RIPA) buffer for further proteomic analysis. Amoxicillin, flucloxacillin, and isoniazid were soluble in cell culture media, whereas nitroso‐sulfamethoxazole was dissolved in 0.05% dimethyl sulfoxide (DMSO). The vehicle cell culture media contained 0.05% DMSO. Hepatocytes from 3 donors were used for the analysis. Supernatant was first centrifuged at 3000*g* for 15 minutes to remove debris. Afterward, supernatant was gently mixed with ExoQuick‐TC solution (5:1; System Biosciences, Palo Alto, CA). The mixture was incubated at 4°C for 12 hours. Samples were then centrifuged at 1500*g* for 30 minutes. Supernatant was discarded and the tubes were centrifuged for a further 5 minutes. Exosomes pellets were suspended in either RIPA buffer or phosphate‐buffered saline (PBS).

### Transmission Electron Microscopy

The exosome suspension was placed on formvar‐coated copper grids for 20 minutes and then washed several times with PBS. Exosomes were then fixed by placing grids on drops of 2% glutaraldehyde on parafilm for 5 minutes. Exosomes were contrasted and embedded by transferring grids to 2% uranyl acetate for 5 minutes and then adding a drop of 0.13% methyl cellulose and 0.4% uranyl acetate for 5 minutes. The grids were visualized using a Philips (Hillsboro, OR) EM 208S transmission electron microscope at 80 kV and images captured with a Gatan (Pleasanton, CA) CCD camera.

### Assessment of the Expression of Human Exosomal Markers and the Presence of Drug‐Modified Exosomal Proteins

Twenty micrograms of protein was separated using 12% sodium dodecyl sulfate–polyacrylamide gel electrophoresis (SDS‐PAGE; 170 V, 75 minutes) and transferred onto a nitrocellulose blotting paper (230 mA, 1 hour). After blocking, blots were incubated in primary antibodies specific for CD9, CD63, CD81, and heat shock protein (HSP) 70 (System Biosciences, Palo Alto, CA) overnight. Anti‐rabbit and anti‐goat horseradish peroxidase–conjugated secondary antibodies were then applied, and the proteins were visualized using enhanced chemoluminescence substrate (Perkin Elmer, the Netherlands).

### Assessment of Drug‐Modified Exosomal Proteins

Drug‐modification within exosomes was visualized by immunoblotting using rabbit anti‐flucloxacillin (custom antibody; Eurogentec, Belgium), rabbit anti‐nitroso‐sulfamethoxazole 1:2,000 (Panigen), and mouse anti‐penicillin (AbD Serotec) antibodies.

### Proteomic Profiling of Hepatocyte‐Derived Exosomes

To investigate changes in exosomal protein expression and drug modification of exosomal proteins, protein lysates were separated using 12% SDS‐PAGE.^27^ Each lane was rehydrated in 10 ng/µL trypsin solution and incubated at 37°C overnight. The peptides were extracted and dried in a SpeedVac, followed by resuspension in 10 µL 0.1% formic acid for LC/MS‐MS analysis. Samples were delivered into a Triple TOF 6600 mass spectrometer (Sciex) by automated in‐line reversed phase liquid chromatography. A gradient of 2%‐50% (vol/vol) Acetonitrile (ACN), 0.1 % (vol/vol) formic acid over 90 minutes was applied to the column at a flow rate of 300 nL/minute. Spectra were acquired automatically in positive ion mode using information‐dependent acquisition powered by Analyst TF 1.7 software (Sciex), using mass ranges of 400‐1,800 atomic mass units (amu) in MS and 100‐1,500 amu in MS‐MS. Up to 25 MS‐MS spectra were acquired per cycle using a threshold of 300 counts per second, with dynamic exclusion for 12 secondss and rolling collision energy.

### Proteomic Data Analysis

Proteins were identified by ProteinPilot software v5.0 (Sciex) using the Paragon algorithm and the SwissProt database with biological modifications allowed. The mass tolerance for both precursor and fragment ions was 10 ppm. The data were also searched against the reversed database, and only proteins that fell within a 1% global false discovery rate were included in further analyses.

Two approaches were used to assess potential differences in the exosomal protein profile as a result of each of the drug treatments. The decision to use two independent approaches for the data analysis was based on the observed heterogeneity of the hepatocyte donors and the passage of time between the acquisition of the first data set and the last.

In approach 1, peptide intensities for each protein derived from ProteinPilot were summed and normalized to total ion count for each exosome sample. The data were sorted in RStudio (version 3.4.0) to produce a composite list of proteins present in every sample and log10 transformed. In approach 2, the data were analyzed using label‐free quantification in MaxQuant:^28^ All settings used were default apart from the minimal Label‐free quantification (LFQ) ratio count being set to 1 and the retention time window being increased to 5 minutes. The data were searched using human UniProt database. The MaxQuant output file was edited to remove contaminants and proteins matching the reverse database. The MS proteomics data have been deposited to the ProteomeXchange Consortium by means of the PRIDE^29^ partner repository with the data set identifier PXD010760.

Data generated were then subjected to further analysis using the Partek Genomics Suite (version 7.18.0130). Hierarchical cluster analysis was performed using standardization, while a correlative principal component analysis (PCA) was performed assuming all variables had an equal influence on the principal components. The data were then batch corrected for donor, and the above analyses were repeated. Two‐way analysis of variance (ANOVA) analysis was performed on the uncorrected data with drug treatment and donor as variables. Proteins significantly associated with drug treatment were classified using PANTHER.

### Characterization of the Uptake of Hepatocyte‐Derived Exosomes by Human Monocyte‐Derived Dendritic Cells

Antibody‐conjugated magnetic bead separation was used to isolate CD14^+^ monocytes from healthy donor peripheral blood mononuclear cells (PBMC). Approval for the extraction of blood from human donors was acquired from the Liverpool local research ethics committee and informed written consent was obtained. Monocytes were then cultured with interleukin (IL)‐4 (800 U/mL) and granulocyte‐macrophage colony‐stimulating factor (GM‐CSF; 800 U/mL) for 7 days to generate dendritic cells. To assess the uptake of hepatocyte‐derived exosomes by dendritic cells, exosomes were first stained with PKH26 red fluorescent dye (Sigma‐Aldrich, United Kingdom). Dendritic cells were then cultured with dye‐stained exosomes on cover slips embedded in 12‐well cell culture plates. In other experiments, dendritic cells were cultured with exosomes isolated from hepatocytes exposed to either flucloxacillin or nitroso‐sulfamethoxazole for 24 hours followed by confocal microscopy to determine exosome uptake.

Dendritic cells were rinsed and fixed with 4% paraformaldehyde. After rinsing and permeabilization, dendritic cells were incubated with primary antibodies, mouse anti‐actin 1:50 (Santa Cruz Biotechnology), and rabbit anti‐flucloxacillin or rabbit anti‐nitroso‐sulfamethoxazole antibodies overnight at 4°C. Actin was visualized using Alexa Fluor chicken anti‐mouse 488 1:500 (molecular probes), whereas flucloxacillin and nitroso‐sulfamethoxazole were visualized using Alexa Fluor chicken anti‐rabbit 1:500. Nuclei were stained with 4´,6‐diamidino‐2‐phenylindole (5 µg/mL; Invitrogen). Cells were visualized using a confocal microscope (Zeiss LSM 800, Germany) and dendritic cells uptake of hepatocyte‐derived exosomes quantified using Fiji software. The amount of hepatocyte‐derived exosomes taken up by dendritic cells was estimated by selecting exosomes within a representative field of view and the fluorescence intensity measured.

### Flow Cytometry Assessment of Activation and Maturation Markers on Dendritic Cells Exposed to Test Drugs and Drug‐Modified Exosomes

To access the effect of hepatocyte‐derived exosomes on dendritic cell activation and maturation, dendritic cells were cultured with exosomes derived from hepatocytes exposed to either cell culture media or the test compounds flucloxacillin and nitroso‐sulfamethoxazole. In order to investigate the direct effect of soluble drug on dendritic cell surface markers, dendritic cells were also exposed to soluble flucloxacillin (0.05 mM) and nitroso‐sulfamethoxazole (0.01 mM) for 24 hours. Drug‐induced changes in the expression of major histocompatibility complex (MHC) class II, CD40, CD80, CD83, and HLA‐DR expression were analyzed by flow cytometry using FACS Canto II system. A total of 10,000 events were acquired.

### Peptide Design and Generation of Amoxicillin‐Modified HLA‐A*02:01 Binding 9‐mer Peptides

Peptide binding prediction of MHC class I peptide epitopes for exosomal proteins was performed using the NetMHCpan Server 4.0 search criteria. The search parameters included restriction to 9mer peptides and the HLA‐A*02:01 or the HLA‐B*57:01 allele, and the threshold for strong binders was set to 0.5%. Epitopes that ranked below 0.5% that contained a central modified residue were considered for immunogenicity studies. Finally, because of cysteine‐containing peptides being difficult to synthesize, these were excluded from the suitability criteria. The peptide SLLEPSVKI was chosen for the immunogenicity studies and was purchased with and without an Fmoc protection group on the n‐terminus from Synpeptide Ltd. The n‐terminus was protected to ensure site‐specific modification of the lysine at position 8 during synthesis.

Fmoc‐SLLEPSVKI was incubated with amoxicillin at a 50:1 molar ratio in 70% ACN/30% H_2_O for 48 hours at 37°C. Analytes were acidified to 0.1% trifluoroacetic acid (TFA) and loaded onto a Phenomenex C_18_ Kinetex 5 µm column coupled with an Agilent 1200 HPLC at λ214. The following HPLC gradient was applied over 30 minutes: (minutes, %B; 0, 2; 20, 75, 20.10, 98; 25, 98; 25.10, 2; 30, 2) with solvent A as 0.1% TFA and solvent B as ACN/0.1% TFA. The modified peptide peak was identified through a series of HPLC experiments to identify candidate peptide peaks. Fractions were collected and pooled from several HPLC runs; this was followed by the addition of piperidine at a 10:1 molar ratio in 70% ACN/30% H_2_O for 24 hours to remove the Fmoc protecting group. The incubation was subjected to the same HPLC conditions to purify the deprotected drug‐modified peptide. A full scheme of peptide purification and optimization is shown in Supporting Fig. S3. Detection of the drug‐modified peptide peak was analyzed using LC/MS‐MS. Samples were resuspended in 0.1% Formic acid (FA)/2% ACN and loaded onto an AB Sciex TripleT of 5600 mass spectrometer for analysis. Peptides were manually sequenced using PeakView 1.2 and annotated with the peptide sequence, modified amino acids, and characteristic fragment ions of amoxicillin.

### Determination of the Activation of Naïve Human T Cells From Healthy HLA‐A*02:01+ Donors With an Amoxicillin‐Modified Sllepsvki Peptide

PBMC from HLA*02:01+ healthy human donors were used to assess the T‐cell immunogenicity of the amoxicillin‐modified SLLEPSVKI peptide. Briefly, PBMC were isolated from venous blood of 3 donors using density centrifugation. This was followed by separation of CD14‐positive monocytes and naïve T cells using antibody‐conjugated magnetic beads. Dendritic cells were generated from the CD14+ monocytes with a cocktail of IL‐4 and GM‐CSF over 7 days. On day 7, dendritic cells were loaded with unmodified (50 µM) or amoxicillin‐modified (50 µM) SLLEPSVKI peptide for 6 hours followed by maturation with lipopolysaccharide and tumor necrosis factor α overnight. Naïve T cells (2 × 10^5^/well; 24‐32 wells per condition depending on the availability of naïve T cells) were cocultured with either unmodified or amoxicillin‐modified peptide pulsed dendritic cells (8 × 10^3^) for 14 days. T cells were then restimulated with a fresh batch of autologous dendritic cells loaded with the unmodified or amoxicillin‐modified peptide. T‐cell proliferative responses were evaluated through the addition of ^3^[H]‐thymidine incorporation for the final 16 hours of the experiment.

### Statistical Analysis

ANOVA or paired *t* test were used for statistical analysis. *P* < 0.05 was considered statistically significant.

## Results

### Morphology and Proteomic Characterization of Hepatocyte‐Derived Exosomes

**Figure 1:**
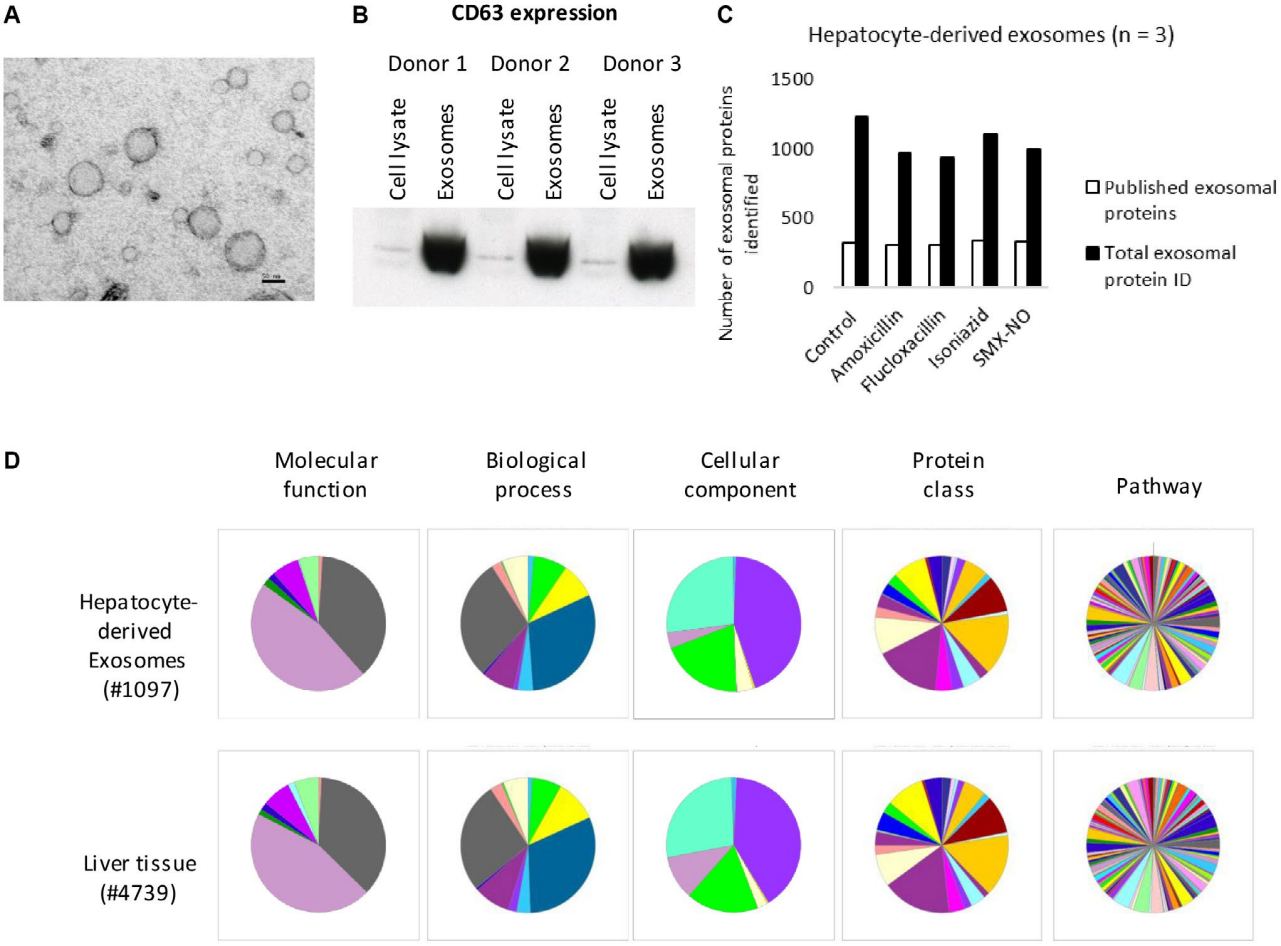
Morphology and proteomic characterization of hepatocyte‐derived exosomes. (A) Hepatocyte‐derived exosomes measured ≤100 nm in diameter, scale bar = 50 nm. (B) CD63 expression by hepatocyte‐derived exosomes and corresponding whole cell lysates from 3 donors determined by immunoblotting using anti‐CD63 specific antibody. (C) Proteins identified in hepatocyte‐derived exosomes by mass spectrometry (n = 3). Dark bars represent total exosomal proteins identified across treatment groups, and open bars represent human exosomal proteins published in the ExoCarta database. (D) Comparison of the whole liver tissue proteome with that of hepatocyte‐derived exosomes based on molecular function, biological process, cellular component, protein class, and pathway using PANTHER gene list analysis.

The hepatocyte‐derived exosomes measured ≤100 nm in diameter (Fig. 1A).^19^ Immunoblotting revealed the expression of CD63 but not CD81, CD9, and HSP70 (Fig. 1B). Furthermore, CD63 was overexpressed in the exosomal fraction compared with the cell lysate. LC/MS analysis identified peptides consistent with CD63, CD81, CD9, and HSP70 protein expression.

On average, approximately 1,200 proteins were identified by LC/MS in untreated hepatocyte‐derived exosomes across 3 donors (Fig. 1C). Comparison of the control hepatocyte exosome proteome with that of the whole liver (in‐house database of 4,739 proteins) revealed that they shared a similar protein profile based on molecular function, biological process, cellular component, protein class, and pathway (Fig. 1D). Furthermore, exosomes expressed high‐mobility group box 1 and HSPs that play important roles in inflammation.^30^

### Characterization of Global Drug‐Induced Changes in Proteins Packaged by Hepatocyte‐Derived Exosomes

**Figure 2:**
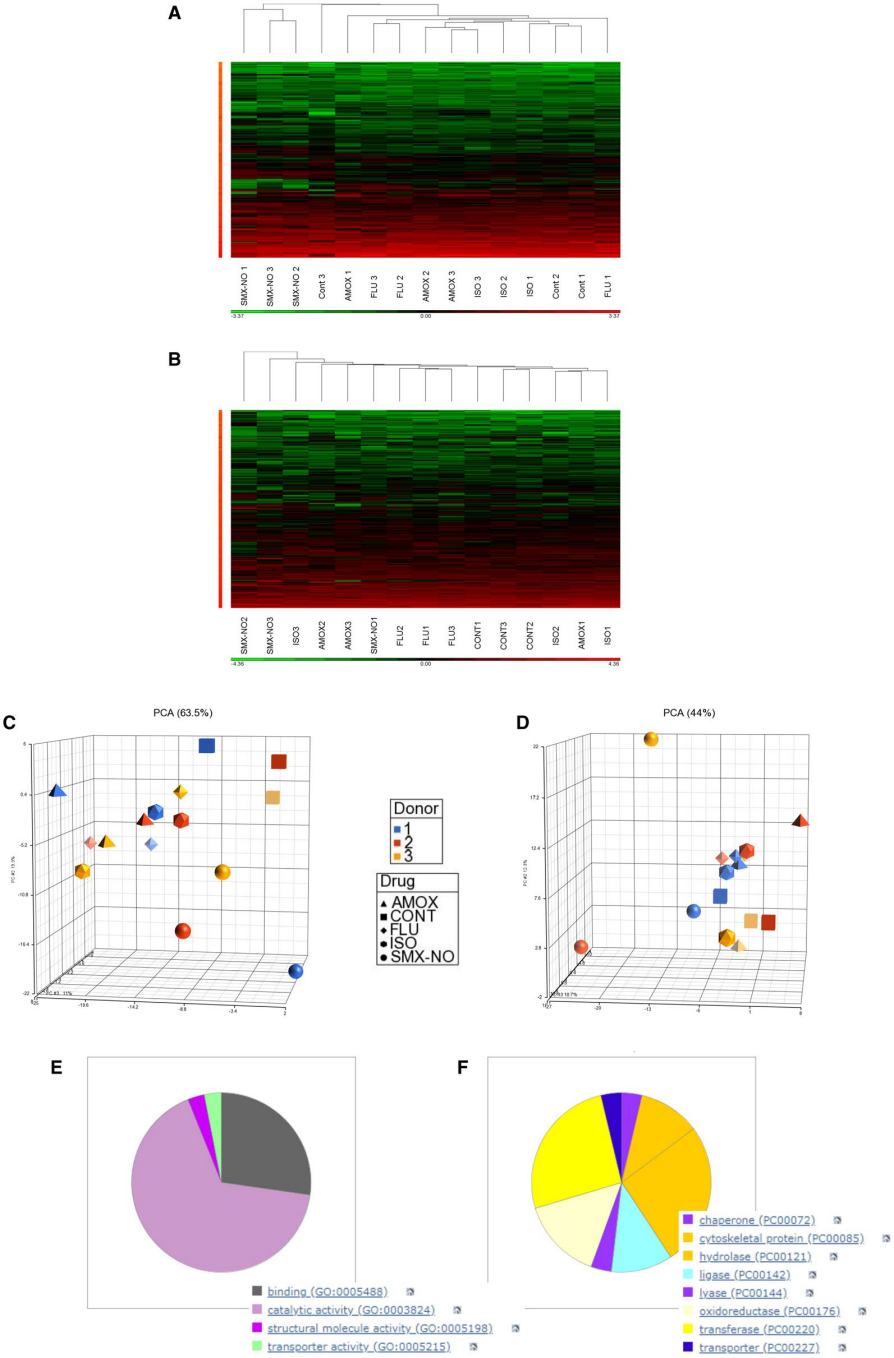
Characterization of global drug‐induced changes in proteins expressed by hepatocyte‐derived exosomes. (A) Hierarchical cluster analysis of data processed using approach 1 and batch corrected for hepatocyte donor. (B) Hierarchical cluster analysis of data processed using approach 2 and batch corrected for hepatocyte donor. Green and red represent low and high protein levels, respectively. (C) PCA of data processed using approach 1 and batch corrected for hepatocyte donor. (D) PCA of data processed using approach 2 and batch corrected for hepatocyte donor. (E) PANTHER molecular function classification of proteins differentially represented in exosomes from nitroso‐sulfamethoxazole‐treated hepatocytes compared with those exposed to all other treatments. (F) PANTHER protein class classification of proteins differentially represented in exosomes from nitroso‐sulfamethoxazole‐treated hepatocytes compared with those exposed to all other treatments.

When all data files were processed together using ProteinPilot, 2,109 proteins were identified within an False Discovery rate (FDR) of 1% (Supporting Table S1 and in the PRIDE repository PXD010760). One third of the proteins identified have previously been published in the ExoCarta database (exocarta.org). There was a subset of 522 proteins for which there was intensity data in every sample (approach 1). MaxQuant identified 1,571 proteins within an FDR of 1%, for which there was quantitative data in every sample for 608 proteins (approach 2). The data were subjected to hierarchical, PCA, and ANOVA analysis using Partek Genomics Suite. Samples treated with nitroso‐sulfamethoxazole appeared to cluster away from the other samples when the data were processed using either approach 1 (Fig. 2A,C) or approach 2 (Fig. 2B,D). Two‐way ANOVA analysis identified 148 proteins from approach 1 and 113 proteins from approach 2 that were significantly (*P* < 0.05) associated with the nitroso‐sulfamethoxazole treatment. Thirty‐five of these proteins were present in both data sets (Supporting Fig. S1) and are listed in Supporting Table S2. Comparing each drug treatment individually with the control exosome sample revealed few significant changes (amoxicillin, 3 proteins; flucloxacillin, 10 proteins; isoniazid, 7 proteins; Supporting Table S3A‐C). However, the nitroso‐sulfamethoxazole sample compared with the control sample exhibited 35 differentially encapsulated proteins (Supporting Table S3D). These overlapped (23/35) with the analysis of nitroso‐sulfamethoxazole compared with all the other treatments combined. PANTHER analysis of the list of 35 proteins revealed that most of them are involved in catalytic activity (Fig. 2E) and that this included a range of enzyme classes (Fig. 2F).

### Covalent Modification of Exosomal Proteins by Amoxicillin, Flucloxacillin, and Nitroso‐ Sulfamethoxazole

**Figure 3:**
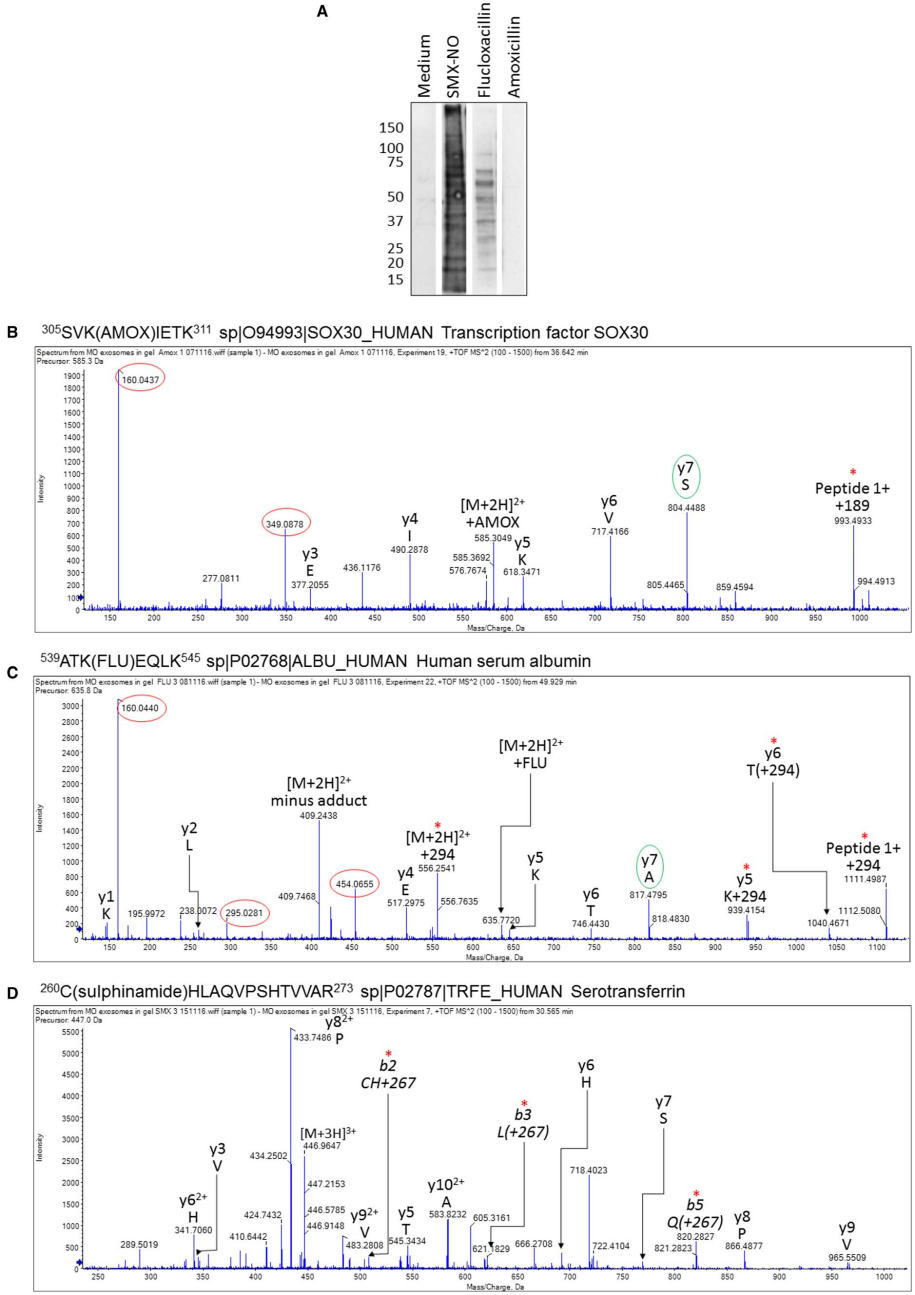
Detection of drug modification of exosomal proteins derived from drug‐treated hepatocytes from a single donor. (A) Western blot characterization of human exosomal proteins modified by either nitroso‐sulfamethoxazole or flucloxacillin using specific antidrug antibodies. (B) MS‐MS spectrum of amoxicillin‐modified peptide from SOX30. (C) MS‐MS spectrum of flucloxacillin‐modified peptide from HSA. (D) MS‐MS spectrum of nitroso‐sulfamethoxazole‐modified peptide from serotransferrin. Fragment ions derived from the drug are circled in red, the N‐terminal y ion is circled in green (resulting from cleavage of the entire drug adduct before cleavage of the peptide backbone), and ions with full or partial drug adducts are marked with an asterisk.

**Figure 4:**
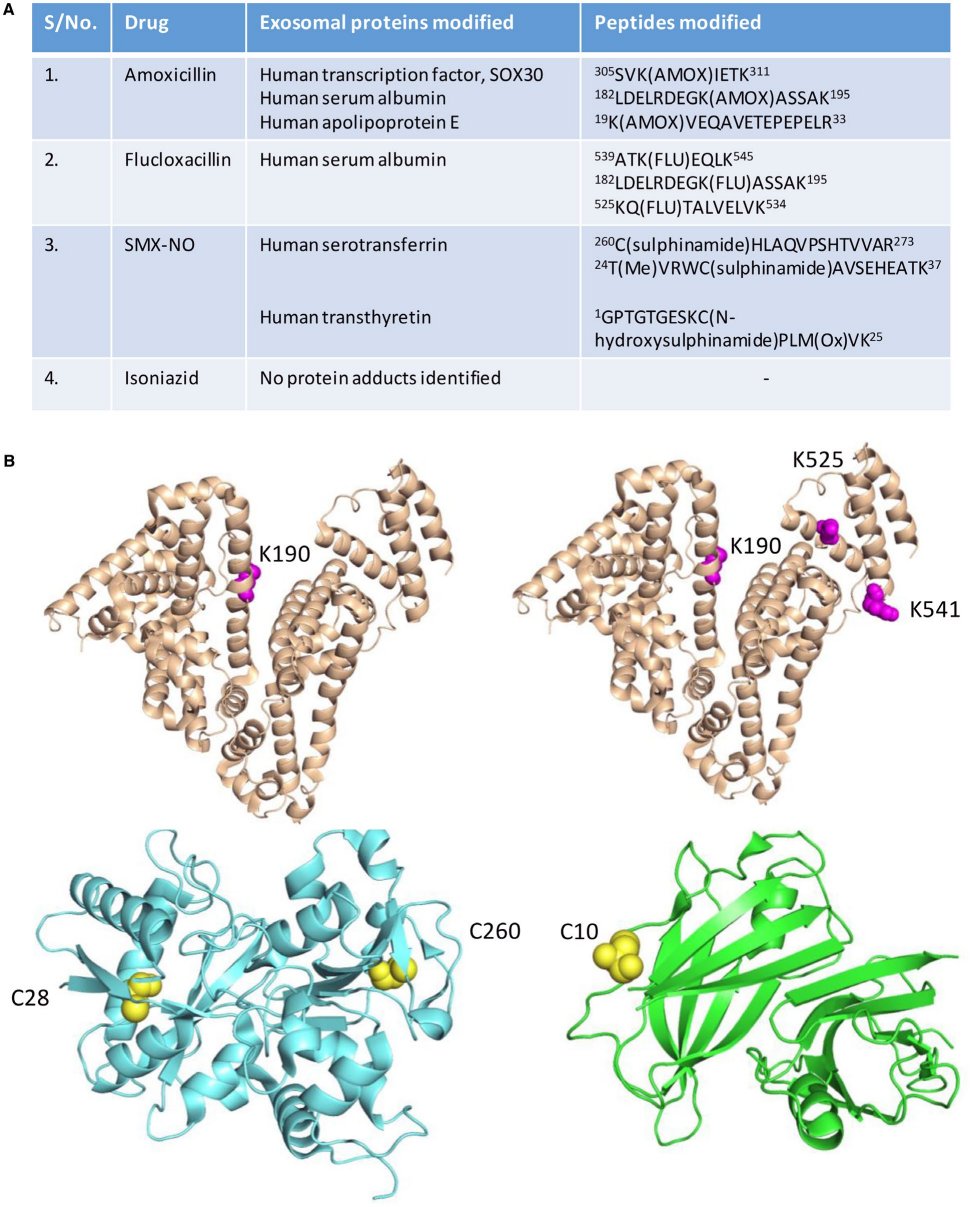
Exosomal proteins modified with drugs. (A) List of the exosomal proteins modified by nitroso‐sulfamethoxazole, flucloxacillin, and amoxicillin and the specific sites of modification. (B‐E) Three‐dimensional structures of exosomal proteins showing the sites of drug modification: (B) HSA (PDB‐1O9X)‐amoxicillin, (C) HSA (PDB‐1O9X)‐flucloxacillin, (D) serotransferrin (PDB‐1DTG)‐sulfinamide, (E) transthyretin (PDB‐4N86)‐N‐hydroxysulfinamide.

Immunoblotting with drug‐specific antibodies detected nitroso‐sulfamethoxazole and flucloxacillin adducts within hepatocyte‐derived exosomes (Fig. 3A). Furthermore, LC/MS‐MS analysis of exosomes revealed amoxicillin modification of lysine residues on human transcription factor SRY (sex determining region Y)‐box 30 (SOX30), albumin, and apolipoprotein E (Figs. 3B, 4A,B). Flucloxacillin‐modified peptides from albumin were also observed (Figs. 3C, 4A,C). In addition, sulfinamide and N‐hydroxysulfinamide adducts on serotransferrin (Figs. 3A, 4A,D) and transthyretin (Fig. 4A,E), respectively, were detected for nitroso‐sulfamethoxazole‐treated samples. Interestingly, both cysteine residues (C28 and C260) on human serotransferrin that were shown to have been modified by nitroso‐sulfamethoxazole would normally be present as disulfides with other cysteine residues. Figure 4A‐E shows a list of all the drug‐modified peptides identified and the structure of the proteins with sites of modification highlighted.

### Human Monocyte‐Derived Dendritic Cell Uptake of Exosomes by Phagocytosis and Endocytosis

**Figure 5:**
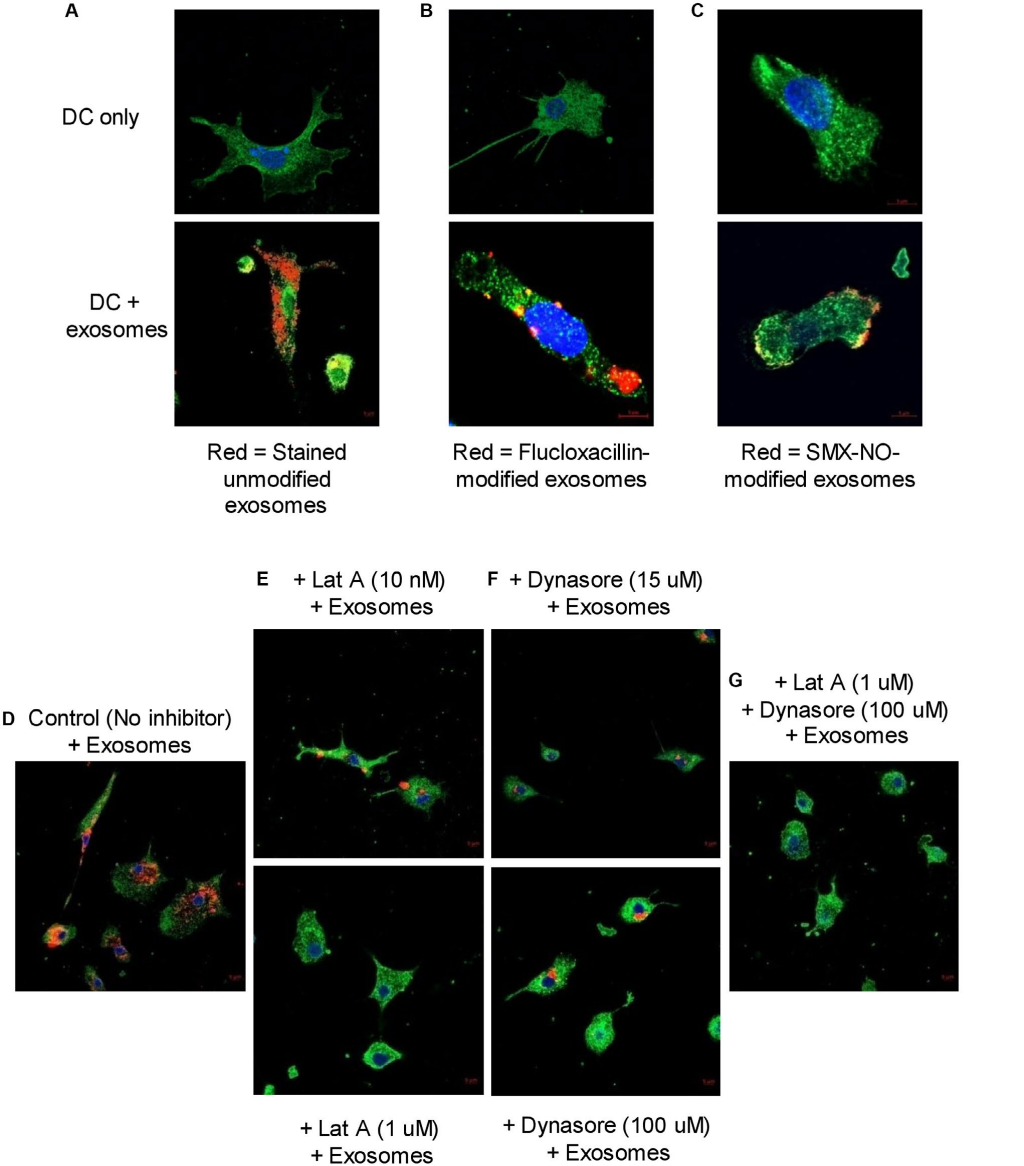
The uptake of unmodified and drug‐modified hepatocyte‐derived exosomes by human monocyte‐derived dendritic cells. Exosomes derived from drug‐treated hepatocytes were cultured with human monocyte‐derived dendritic cells for 24 hours and exosome uptake was evaluated using confocal microscopy. Scale bar represents 5 μm. Green = F‐actin, blue = nucleus, red = unmodified or drug‐modified exosomes. (A) Uptake of unmodified hepatocyte‐derived exosomes by dendritic cells. Exosomes were first stained red with PKH26 dye before 24‐hour culture with dendritic cells and confocal microscopy. (B,C) Uptake of flucloxacillin‐ and nitroso‐sulfamethoxazole‐modified hepatocyte‐derived exosomes by dendritic cells, respectively. Exosomes derived from drug‐treated hepatocytes were cultured with dendritic cells for 24 hours, and drug‐specific antibodies were used to tag drug‐modified exosomal proteins followed by confocal microscopy to determine the uptake of drug‐modified exosomes. (D) Exosome uptake by dendritic cells in the absence of inhibitor. (E) The effect of latrunculin A (phagocytosis inhibitor) on dendritic cell exosome uptake. (F) Effect of dynasore (endocytosis inhibitor) on dendritic cell exosome uptake. (G) Combined effect of latrunculin A and dynasore on dendritic cell exosome uptake. Abbreviation: DC, dendritic cell.

Dendritic cells were found to engulf both unmodified and drug‐modified hepatocyte‐derived exosomes (Fig. 5A‐C; Supporting Fig. S2A). Figure 5A shows exosomes stained with PKH26 red fluorescent dye, whereas Fig. 5B,C show red staining with anti‐flucloxacillin and anti‐nitroso‐sulfamethoxazole antibodies, respectively. Pretreatment of dendritic cells with either latrunculin‐A (phagocytosis inhibitor) or dynasore (endocytosis inhibitor) or both inhibitors for 2 hours before addition of hepatocyte‐derived exosomes for 24 hours resulted in a significant reduction or inhibition of exosome uptake (Fig. 5D‐G; Supporting Fig. S2B).

### Effect of Hepatocyte‐Derived Exosomes on the Activation of Human Monocyte‐Derived Dendritic Cells

**Figure 6:**
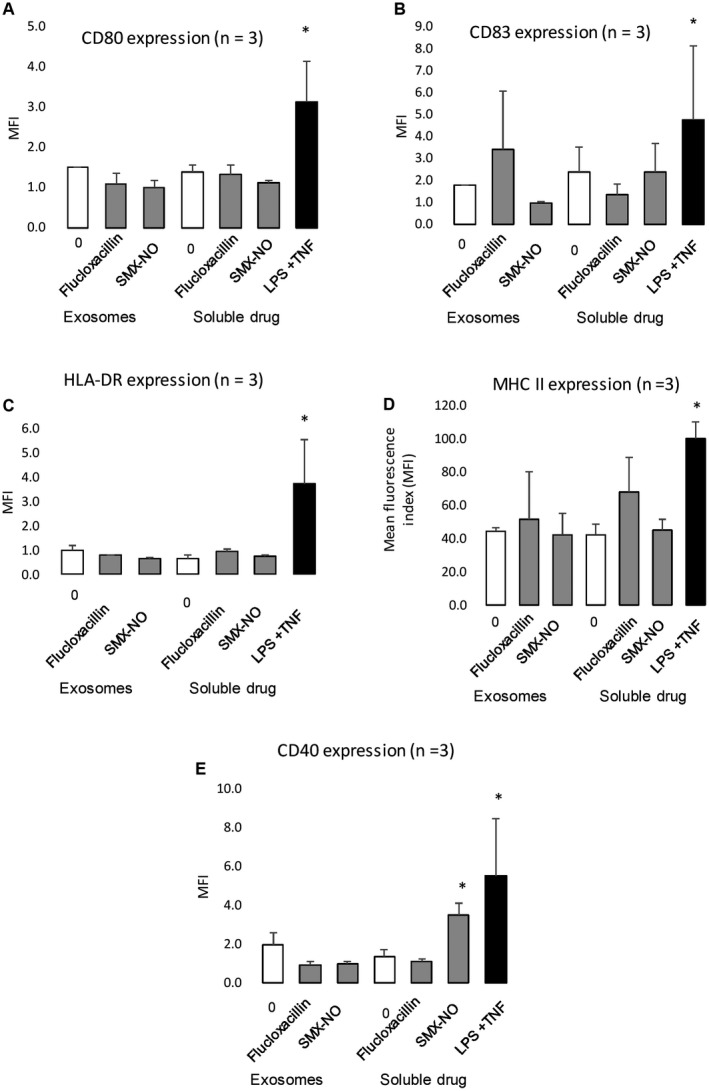
Effect of hepatocyte‐derived exosomes on dendritic cell surface marker expression. Dendritic cells were cultured with exosomes derived from either cell culture media– or drug‐treated hepatocytes or soluble drugs for 24 hours before analysis of (A‐C) maturation markers (CD80, CD83, HLA‐DR) and (D,E) activation markers (MHC class II, CD40) by flow cytometry. Dendritic cells were then stained with fluorochrome‐conjugated antibodies and the expression of markers was evaluated using a FACS Canto II flow cytometer. A minimum of 10,000 events were acquired.

Hepatocyte‐derived exosomes did not significantly alter the expression of either dendritic cell maturation or activation markers (Fig. 6). Furthermore, flucloxacillin and nitroso‐sulfamethoxazole exposure did not interfere with the expression of most markers. However, as described, nitroso‐sulfamethoxazole treatment resulted in a significant increase in the expression of CD40.^31^ Binding of CD40 on antigen presenting cells to CD40L on helper T cells activates the antigen presenting cells leading to a pro‐inflammatory response.^32^ Dendritic cell interferon‐γ and IL‐10 secretion were also assessed by ELIspot after drug or exosome treatment. No significant increase in secretion of either cytokine was observed.

### Activation of Naïve T Cells From HLA‐A*02:01 Human Donors With the Amoxicillin‐Modified 9‐mer Peptide Sllepsvki

**Figure 7:**
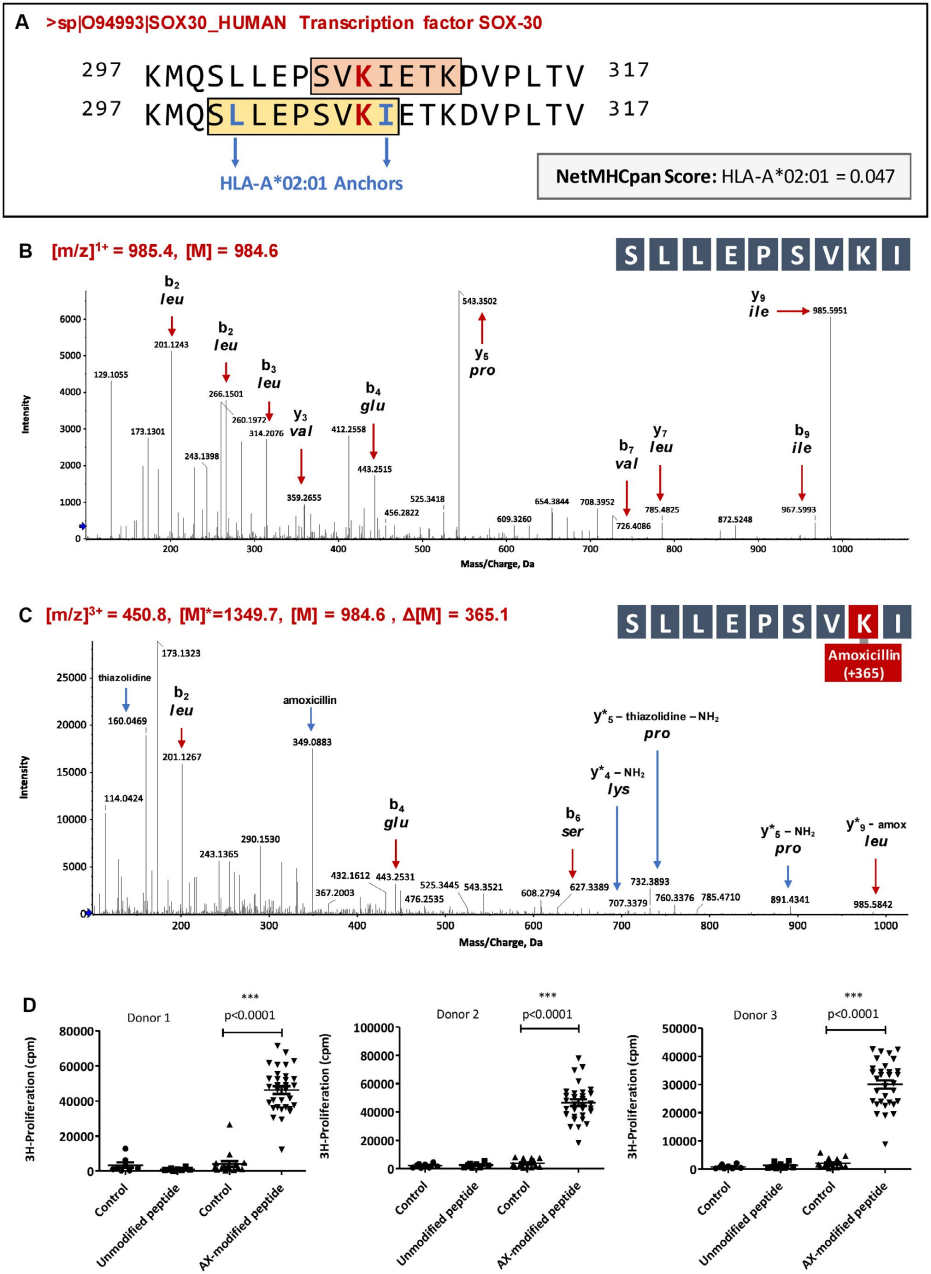
LC/MS‐MS characterization of an amoxicillin‐modified HLA‐A*02:01 binding peptide derived from transcription factor protein SOX30 and functional evaluation of T‐cell immunogenicity. (A) SLLEPSVKI peptide design rationale. The tryptic peptide identified in exosomal proteins is highlighted in orange with the SOX30 amino acid sequence expanded to include 10 amino acids in either direction of the modified lysine. The HLA‐A*02:01 binding epitope is highlighted in yellow labeled with the anchor residues in blue. The NetMHCpan score for this peptide was 0.047. (B) MS‐MS spectrum of the unmodified SLLEPSVKI peptide derived from SOX30. (C) MS‐MS spectrum of the amoxicillin modified SLLEPSVKI peptide derived from SOX30. Fragment ion peaks of the peptide sequence have been labeled with a red arrow and drug associated peaks labeled with a blue arrow. (D) Naïve T cells from 3 HLA‐A*02:01+ human donors were cocultured with unmodified or amoxicillin‐modified peptide‐pulsed monocyte‐derived dendritic cells at a 25:1 ratio in a 96 U‐bottomed plate (24‐32 wells per condition), in a final volume of 200 µL for 14 days. Cultures were restimulated with a fresh batch of autologous peptide loaded dendritic cells for 48 hours. ^3^[H]‐thymidine was added during the final 16 hours of culture to measure the extent of T‐cell proliferation. Wells were harvested and incorporated radioactivity analyzed using a beta counter. Each data point represents proliferative T‐cell responses in a single well of a 96‐well plate.

Assessing the immunogenicity of whole proteins or exosomes *per se* was not possible. Therefore, peptides containing the drug‐modified residues identified on the exosomal proteins aligned to relevant HLA binding epitopes were synthesized to assess their immunogenicity. To do this, protein sequences were expanded to include 10 amino acids in both directions from the modified residue, and these sequences were inputted into the NetMHCpan 4.0 server to search for potential MHC class I epitopes. Drug‐induced liver injury to amoxicillin and flucloxacillin are associated with the class I alleles HLA‐A*02:01 and HLA‐B*57:01, respectively, whereas Sulfamethoxazole (SMX) has not shown any specific association with HLA alleles. Therefore, these alleles were selected to assess potential peptide epitopes for all four modified proteins. Peptide epitopes were selected for study if they scored less than 0.5 on the NetMHCpan 4.0 server and only if the modified residue fell within the anchor motifs. No peptide epitopes met this selection criteria for HLA‐B*57:01, although strong binding epitopes for HLA‐A*02:01 were identified for the amoxicillin‐modified SOX30 transcription factor protein (SLLEPSVKI ‐ NetMHCpan 4.0 Score = 0.047) and the amoxicillin‐modified apolipoprotein e protein (FLAGCQAKV ‐ NetMHCpan 4.0 Score = 0.128). Both epitopes contained a modified lysine in position 8 falling within the P2 and PΩ anchors for HLA‐A*02:01; however, the apolipoprotein peptide sequence also contained a central cysteine residue. Previous studies synthesizing drug‐modified peptides have shown that free cysteines cause difficulty as a result of peptide dimerization during synthesis resulting in low product yield and multiple oxidation states complicating assay variability. As such, the amoxicillin‐modified peptide epitope for HLA‐A*02:01, SLLEPSVK(Amox)I derived from SOX30 was the only epitope that met suitable criteria for immunogenicity studies (Fig. 7A). The unmodified and amoxicillin‐modified peptides yielded the expected peptide fragmentation pattern, with characteristic fragments for amoxicillin including a peak at 160 da indicative of the cleaved thiazolidine ring and a peak at 349 da indicative of the whole cleaved amoxicillin molecule. Several drug‐modified fragment peaks were also identified (Fig. 7B,C; Supporting Fig. S3).

Naïve T cells isolated from three HLA‐A*02:01‐positive donors were cocultured with autologous monocyte‐derived dendritic cells that had been pulsed with either unmodified or amoxicillin‐modified SLLEPSVKI peptide, derived from the transcription factor protein SOX30, for 2 weeks. The primed T cells were then restimulated with a second batch of peptide‐loaded dendritic cells before detection of proliferation using [^3^H] thymidine. Naïve T cells from the amoxicillin‐modified peptide‐primed cultures were stimulated to proliferate when restimulated with the amoxicillin‐modified peptide (Fig. 7D; *P* < 0.0001). In contrast, no proliferative response was observed in similar experiments using dendritic cells pulsed with the unmodified peptide.

## Discussion

Idiosyncratic drug reactions targeting the liver are a major challenge for patients, clinicians, and the pharmaceutical industry because there is currently no way to accurately predict which individuals will develop tissue injury. This is partly because the adverse reactions are associated with individual susceptibility factors. The discovery of (1) strong associations between DILI and expression of specific HLA alleles and (2) the selective drug‐specific activation of T cells from patients with DILI indicates that the adaptive immune system is involved in the disease pathogenesis. However, the reason why a drug activates T cells that target the liver has not been defined. One possibility is that drugs that induce liver injury form covalent adducts with critical hepatic proteins and that these adducts are taken up and processed within dendritic cells before presentation of the derived peptides to T cells. Activation of the T cells within the vicinity of the covalently modified hepatic proteins would result in localized tissue damage. Given the importance of exosomes in the crosstalk between tissue cells and the immune system and the accumulation of T cells in liver of patients with early‐stage tissue injury,^12^, ^13^ we sought to understand the impact of drug exposure on the selective packaging and sorting of hepatocyte components into exosomes as well as the interaction between hepatocyte‐derived exosomes and immune cells. Of particular importance was the detailed assessment of exosomal proteins to identify sites of drug modification. Our study focused on three of the drugs commonly associated with idiosyncratic DILI in humans: amoxicillin, flucloxacillin, and isoniazid.^26^ Although primary human hepatocytes express drug‐metabolizing enzymes for up to 24 hours,^33^ each drug selected has been shown to forms adducts with lysine residues on protein spontaneously, with no requirement for drug metabolism.^34^, ^35^, ^36^ Furthermore, we used nitroso‐sulfamethoxazole, a synthetic metabolite of sulfamethoxazole that binds covalently to cysteine residues.^37^ Drug‐responsive T cells have been isolated from the peripheral blood of patients with DILI exposed to each of these drugs, confirming the immune pathogenesis.^9^, ^10^, ^11^

Global analysis of the hepatocyte exosome proteome identified 2,109 proteins; of these, one third (681) of the proteins have previously been published in the ExoCarta database, which includes 5,402 exosomal proteins derived from all tissues. Those listed in the database as having been derived from human liver (326 proteins) were predominantly identified in hepatocellular carcinoma cell lines. Of these, only 60 proteins were observed in the data set presented here. This indicates that the exosome proteome derived from freshly isolated hepatocytes is markedly different to that of cancer cell lines. This study has provided the most complete proteome for hepatocyte‐derived exosomes to date.

Comparison of the hepatocyte‐derived exosome proteome with the whole liver proteome revealed that the distribution of proteins in terms of function, class, and pathways was essentially the same between the two sources of hepatic material. This suggested that proteins were passively captured rather than selectively packaged into the vesicles. Although there is evidence for enrichment of RNA species and certain membrane‐associated proteins in exosomes, the case for cytosolic proteins is less clear. When intraluminal vesicles are formed, they encapsulate cytosolic proteins in their lumen, which remain in place as the vesicle matures into an exosome.^38^ Data presented here do indeed suggest that the hepatocyte exosome proteome provides a snapshot of the cytosolic proteome.

In order to determine whether drug treatment of hepatocytes resulted in a different subset of proteins being packaged and exported, a label‐free, semiquantitative analysis was performed. Exosomes isolated from hepatocytes exposed to nitroso‐sulfamethoxazole were most dissimilar. Thirty five proteins were revealed to be associated with the nitroso‐sulfamethoxazole‐treated sample. Because the data presented here suggest that the exosomal proteome reflects the cellular proteome from which it is derived, we can extrapolate that the catalytic makeup of the hepatocyte is altered by treatment with nitroso‐sulfamethoxazole. Several enzymes involved in xenobiotic metabolism, including epoxide hydrolase, uridine diphosphoglucuronate glucuronyl transferase, and arylacetamide deacetylase, are present at lower levels in the exosomes from nitroso‐sulfamethoxazole‐treated hepatocytes. This suggests a possible dysfunction in pathways involved in response to chemical stress on treatment with the metabolite of sulfamethoxazole.

Drug protein adducts play an important role in immune activation. T cells from patients with flucloxacillin‐ and amoxicillin clavulanate–induced DILI and sulfamethoxazole hypersensitivity can be activated through a pathway that is dependent on the processing of the protein adducts.^9^, ^10^ Recently, Sanchez‐Gomez et al. demonstrated that amoxicillin forms adducts with cellular proteins and that these adducts are detectable in exosomes found in B‐cell culture supernatant.^39^ Hence, the exosome proteome derived from drug‐treated hepatocytes was screened for drug modification. Adducts of amoxicillin, flucloxacillin, and nitroso‐sulfamethoxazole were detected on a total of five exosomal proteins. One of these, albumin, is targeted *in vitro* and *in vivo* by different β‐lactam antibiotics, and the modified protein stimulates patient T cells *in vitro*.^9^, ^34^, ^36^ These data support the hypothesis originally proposed by Sanchez‐Gomez et al. that drug‐modified proteins are encapsulated and transported within exosomes to the extracellular matrix. Importantly, however, the data also identifies hepatic protein targets modified by DILI drugs that might be critical for T‐cell activation.

In order for haptenated proteins to trigger an immune response, they must be taken up by antigen presenting cells, processed, and presented to T cells in an HLA‐restricted manner. Exosomes were shown to be internalized by both phagocytosis and endocytosis. Exosomes containing nitroso‐sulfamethoxazole‐ and flucloxacillin‐modified proteins were also internalized. Hence, our data show that exosomes transfer tissue‐derived drug‐modified proteins that have the potential to act as neoantigens.

To investigate whether drug‐modified exosomal proteins activate T cells, we used *in silico* peptide HLA binding software to identify the 9‐mer peptide sequence containing an amoxicillin modification that was most likely to bind to HLA‐A*02:01 (an HLA class I allele associated with amoxicillin‐clavulanate‐induced liver injury^2^). Restimulation of amoxicillin‐modified peptide primed T cells with peptide‐loaded dendritic cells resulted in a significant proliferative response in all 3 donors. In contrast, the unmodified peptide subjected to the same extraction protocol did not activate the patient T cells. These data demonstrate that the adducts generated in hepatocytes and transported to dendritic cells through exosomes can trigger antigen‐specific T‐cell responses and provide a foundation to explore in more detail the relationship between expression of specific HLA alleles and the development of drug‐induced liver injury.

Danger signals stimulate Toll‐like receptors and NOD‐like receptor family and pyrin containing 3 (NLRP3), and this results in dendritic cell maturation, which can be visualized by up‐regulation of cell surface receptors and cytokine secretion.^40^ Haptenic chemicals and certain drugs have also been shown to stimulate dendritic cell maturation *in vitro*.^32^ Thus, the final objective of this project was to determine whether dendritic cell uptake of hepatocyte‐derived drug‐modified proteins encapsulated within exosomes occurs silently or with a concurrent stress response. The uptake of hepatic exosomes with or without drug treatment did not trigger increased dendritic cell surface receptor expression or cytokine release. These data show that drug‐modified proteins can be transferred from tissue cells to the immune system through exosome transport to ultimately be displayed as peptide fragments on HLA molecules on the surface of dendritic cells without stimulating a stress response.

In conclusion, we have demonstrated a significant heterogeneity in the proteome of exosomes isolated from hepatocytes obtained from different individuals. Exosomes derived from nitroso‐sulfamethoxazole‐treated hepatocytes contained a subset of proteins with altered levels of representation, suggesting perturbation of the stress response in hepatocytes exposed to nitroso‐sulfamethoxazole. Furthermore, the drug‐modified intracellular hepatic proteins transported by exosomes to dendritic cells serve as antigenic determinants for immune activation.
