# Supplementary material for: Exosomal Transport of Hepatocyte‐Derived Drug‐Modified Proteins to the Immune System
Source: Hepatology. 2019 Jun 29;70(5):1732–49. doi: 10.1002/hep.30701 (PMC6899733; doi:10.1002/hep.30701)
Supplement: Supplementary file 1 [file HEP-70-1732-s001.pdf]

Supplementary Figure 1

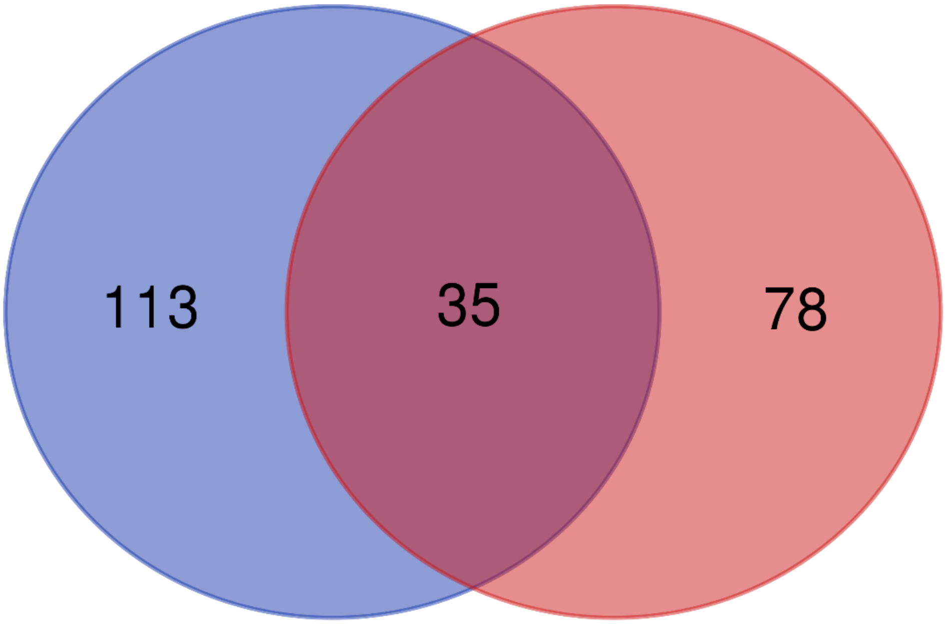

Supplementary Figure 2

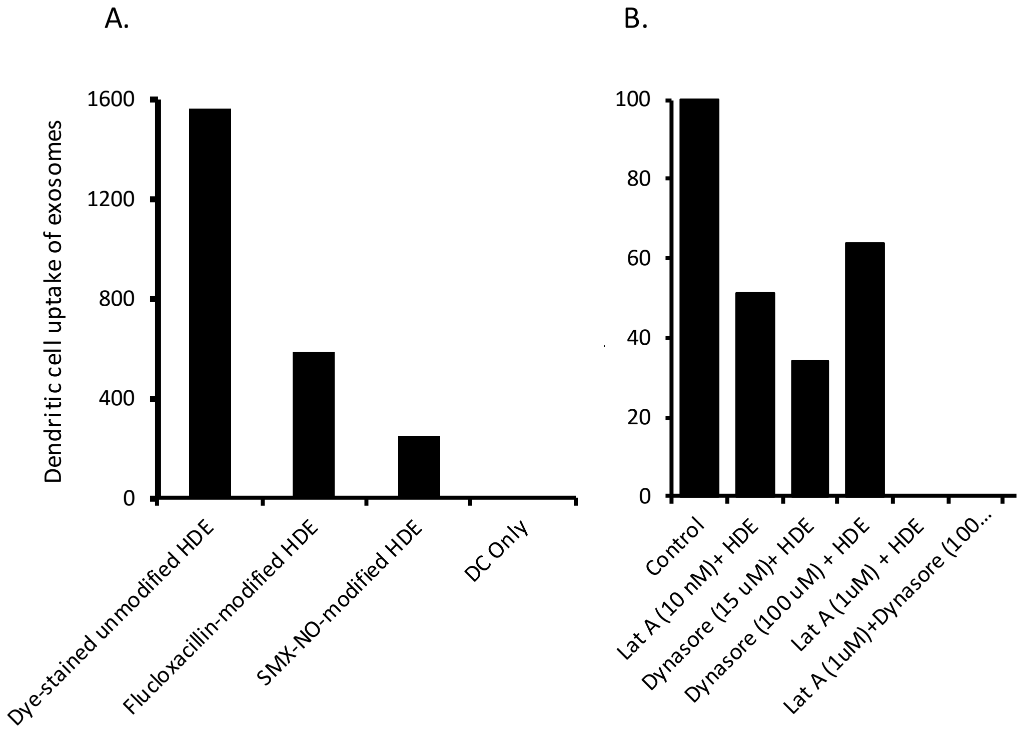

Supplementary Figure 3

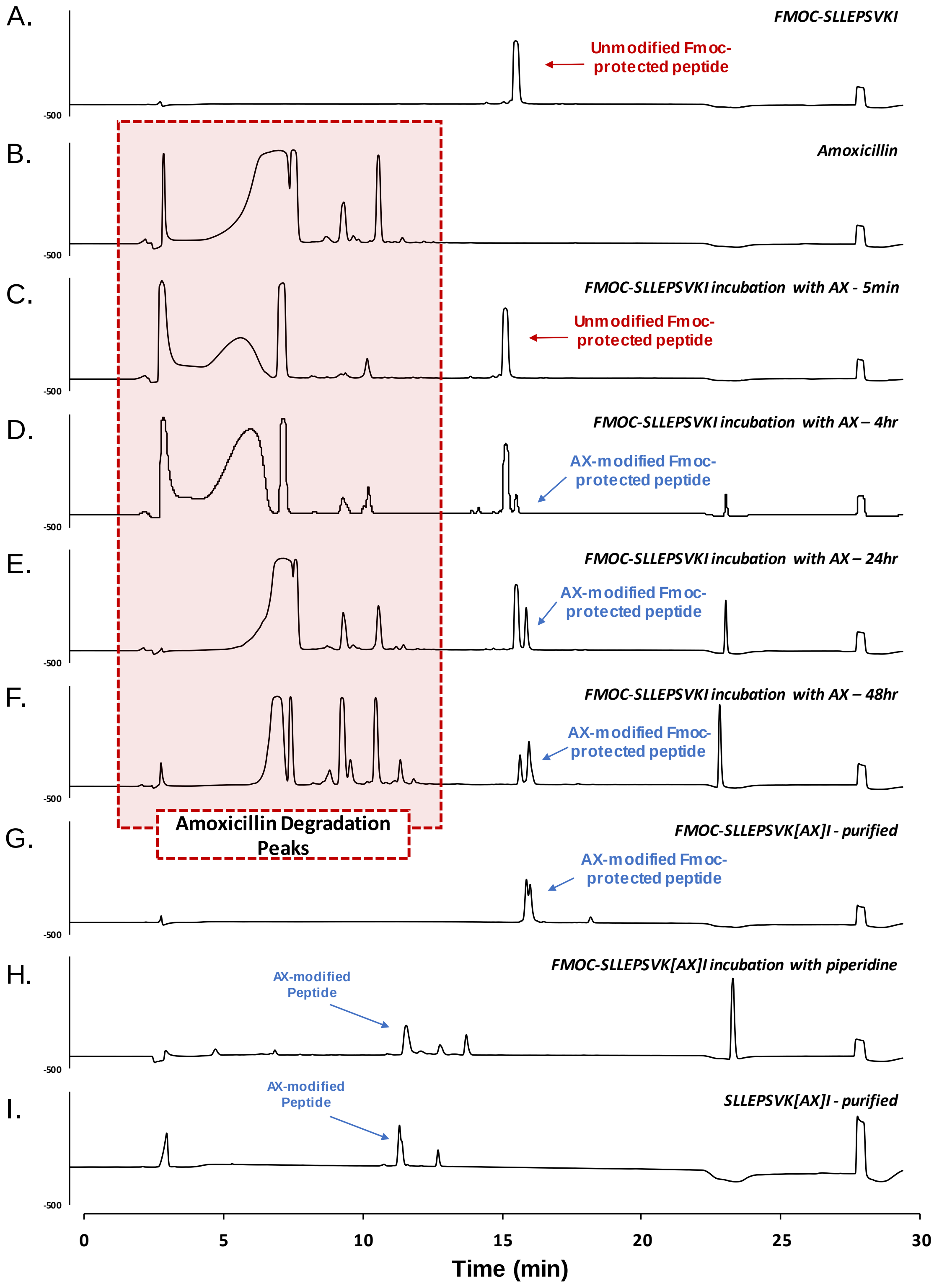

**Supplementary Table 1** Accession no.	Protein	% sequence coverage at 95% confidence	No. non-unique peptides at 95% confidence
sp|Q13813|SPTN1_HUMAN	Spectrin alpha chain, non-erythrocytic 1	71.40	526
sp|Q15149|PLEC_HUMAN	Plectin	37.98	296
sp|Q01082|SPTB2_HUMAN	Spectrin beta chain, non-erythrocytic 1	67.05	416
sp|O75369|FLNB_HUMAN	Filamin-B	64.49	306
sp|P04114|APOB_HUMAN	Apolipoprotein B-100	34.76	148
sp|Q09666|AHNK_HUMAN	Neuroblast differentiation-associated protein AHNAK	45.70	152
sp|P01024|CO3_HUMAN	Complement C3	72.10	412
sp|P31327|CPSM_HUMAN	Carbamoyl-phosphate synthase [ammonia], mitochondrial	71.00	432
sp|P06737|PYGL_HUMAN	Glycogen phosphorylase, liver form	76.86	492
sp|P35579|MYH9_HUMAN	Myosin-9	45.10	150
sp|Q15075|EEA1_HUMAN	Early endosome antigen 1	57.62	176
sp|P12270|TPR_HUMAN	Nucleoprotein TPR	38.26	98
sp|P02751|FINC_HUMAN	Fibronectin	50.34	331
sp|Q9Y490|TLN1_HUMAN	Talin-1	39.98	104
sp|P05787|K2C8_HUMAN	Keratin, type II cytoskeletal 8	83.85	423
sp|Q16851|UGPA_HUMAN	UTP--glucose-1-phosphate uridylyltransferase	85.63	419
sp|Q9P2E9|RRBP1_HUMAN	Ribosome-binding protein 1	54.04	253
sp|P0C0L4|CO4A_HUMAN	Complement C4-A	49.54	173
sp|P05783|K1C18_HUMAN	Keratin, type I cytoskeletal 18	84.65	324
sp|Q86UP2|KTN1_HUMAN	Kinectin	54.61	110
sp|P55072|TERA_HUMAN	Transitional endoplasmic reticulum ATPase	76.05	211
sp|Q06278|AOXA_HUMAN	Aldehyde oxidase	55.23	224
sp|P35573|GDE_HUMAN	Glycogen debranching enzyme	49.35	106
sp|P07099|HYEP_HUMAN	Epoxide hydrolase 1	81.54	273
sp|P08133|ANXA6_HUMAN	Annexin A6	72.07	145
sp|P23141|EST1_HUMAN	Liver carboxylesterase 1	77.60	228
sp|P02768|ALBU_HUMAN	Albumin	72.74	234
sp|O75891|AL1L1_HUMAN	Cytosolic 10-formyltetrahydrofolate dehydrogenase	67.29	116
sp|O15020|SPTN2_HUMAN	Spectrin beta chain, non-erythrocytic 2	34.39	89
sp|P04264|K2C1_HUMAN	Keratin, type II cytoskeletal 1	72.98	267
sp|P49327|FAS_HUMAN	Fatty acid synthase	27.04	61
sp|P00325|ADH1B_HUMAN	Alcohol dehydrogenase 1B	82.67	276
sp|P02545|LMNA_HUMAN	Prelamin-A/C	63.25	175
sp|P07900|HS90A_HUMAN	Heat shock protein HSP 90-alpha	52.05	94
sp|O43707|ACTN4_HUMAN	Alpha-actinin-4	63.23	109
sp|P35908|K22E_HUMAN	Keratin, type II cytoskeletal 2 epidermal	84.04	179
sp|P02649|APOE_HUMAN	Apolipoprotein E	81.07	295
sp|P11021|GRP78_HUMAN	78 kDa glucose-regulated protein	53.98	94
sp|P15144|AMPN_HUMAN	Aminopeptidase N	41.26	83
sp|P35527|K1C9_HUMAN	Keratin, type I cytoskeletal 9	82.34	174
sp|O60437|PEPL_HUMAN	Periplakin	24.66	46
sp|P01023|A2MG_HUMAN	Alpha-2-macroglobulin	37.52	62
sp|P30101|PDIA3_HUMAN	Protein disulfide-isomerase A3	70.10	101
sp|P29144|TPP2_HUMAN	Tripeptidyl-peptidase 2	43.39	54
sp|P11586|C1TC_HUMAN	C-1-tetrahydrofolate synthase, cytoplasmic	49.41	50
sp|P13667|PDIA4_HUMAN	Protein disulfide-isomerase A4	52.40	56
sp|Q7Z406|MYH14_HUMAN	Myosin-14	23.76	59
sp|P13639|EF2_HUMAN	Elongation factor 2	51.05	70
sp|P02788|TRFL_HUMAN	Lactotransferrin	66.20	114
sp|P26038|MOES_HUMAN	Moesin	53.03	66
sp|P13645|K1C10_HUMAN	Keratin, type I cytoskeletal 10	67.47	173
sp|P10809|CH60_HUMAN	60 kDa heat shock protein, mitochondrial	69.81	91

sp|P14625|ENPL_HUMAN	Endoplasmin	46.82	67
sp|Q00610|CLH1_HUMAN	Clathrin heavy chain 1	27.52	50
sp|Q07954|LRP1_HUMAN	Prolow-density lipoprotein receptor-related protein 1	9.38	40
sp|P05023|AT1A1_HUMAN	Sodium/potassium-transporting ATPase subunit alpha-1	38.51	59
sp|P00352|AL1A1_HUMAN	Retinal dehydrogenase 1	64.87	141
sp|P0DMV8|HS71A_HUMAN	Heat shock 70 kDa protein 1A	59.44	87
sp|Q9P2M7|CING_HUMAN	Cingulin	29.07	40
sp|P02787|TRFE_HUMAN	Serotransferrin	56.73	127
sp|P56199|ITA1_HUMAN	Integrin alpha-1	28.67	63
sp|Q13576|IQGA2_HUMAN	Ras GTPase-activating-like protein IQGAP2	28.63	39
sp|P00367|DHE3_HUMAN	Glutamate dehydrogenase 1, mitochondrial	60.57	133
sp|Q9UPN3|MACF1_HUMAN	Microtubule-actin cross-linking factor 1, isoforms 1/2/3/5	5.67	35
sp|P20700|LMNB1_HUMAN	Lamin-B1	50.00	44
sp|Q7KZF4|SND1_HUMAN	Staphylococcal nuclease domain-containing protein 1	44.07	50
sp|P02647|APOA1_HUMAN	Apolipoprotein A-I	86.89	190
sp|P05165|PCCA_HUMAN	Propionyl-CoA carboxylase alpha chain, mitochondrial	50.96	45
sp|P18206|VINC_HUMAN	Vinculin	40.48	46
sp|P05062|ALDOB_HUMAN	Fructose-bisphosphate aldolase B	60.71	185
sp|P09871|C1S_HUMAN	Complement C1s subcomponent	56.25	120
sp|P08319|ADH4_HUMAN	Alcohol dehydrogenase 4	84.21	157
sp|P07237|PDIA1_HUMAN	Protein disulfide-isomerase	68.50	107
sp|P36871|PGM1_HUMAN	Phosphoglucomutase-1	66.73	69
sp|Q16531|DDB1_HUMAN	DNA damage-binding protein 1	38.07	43
sp|O60610|DIAP1_HUMAN	Protein diaphanous homolog 1	30.90	38
sp|P08670|VIME_HUMAN	Vimentin	60.73	56
sp|P04040|CATA_HUMAN	Catalase	65.28	67
sp|Q8IUD2|RB6I2_HUMAN	ELKS/Rab6-interacting/CAST family member 1	32.53	34
sp|P11216|PYGB_HUMAN	Glycogen phosphorylase, brain form	55.63	101
sp|P35221|CTNA1_HUMAN	Catenin alpha-1	44.15	55
sp|P04406|G3P_HUMAN	Glyceraldehyde-3-phosphate dehydrogenase	80.90	123
sp|P22314|UBA1_HUMAN	Ubiquitin-like modifier-activating enzyme 1	43.01	65
sp|P55157|MTP_HUMAN	Microsomal triglyceride transfer protein large subunit	36.24	40
sp|P12814|ACTN1_HUMAN	Alpha-actinin-1	56.73	73
sp|P02671|FIBA_HUMAN	Fibrinogen alpha chain	37.88	74
sp|P47989|XDH_HUMAN	Xanthine dehydrogenase/oxidase	30.91	69
sp|P06733|ENOA_HUMAN	Alpha-enolase	60.37	97
sp|P11142|HSP7C_HUMAN	Heat shock cognate 71 kDa protein	61.46	115
sp|P01009|A1AT_HUMAN	Alpha-1-antitrypsin	72.49	91
sp|P00966|ASSY_HUMAN	Argininosuccinate synthase	66.02	87
sp|Q9NYU2|UGGG1_HUMAN	UDP-glucose:glycoprotein glucosyltransferase 1	21.80	32
sp|P05164|PERM_HUMAN	Myeloperoxidase	51.54	95
sp|Q10567|AP1B1_HUMAN	AP-1 complex subunit beta-1	37.62	36
sp|P22033|MUTA_HUMAN	Methylmalonyl-CoA mutase, mitochondrial	50.40	55
sp|P12956|XRCC6_HUMAN	X-ray repair cross-complementing protein 6	46.31	43
sp|P02675|FIBB_HUMAN	Fibrinogen beta chain	58.25	87
sp|P00738|HPT_HUMAN	Haptoglobin	70.44	199
sp|P00558|PGK1_HUMAN	Phosphoglycerate kinase 1	62.59	56
sp|P50990|TCPQ_HUMAN	T-complex protein 1 subunit theta	52.55	33
sp|P06576|ATPB_HUMAN	ATP synthase subunit beta, mitochondrial	68.24	74
sp|P60709|ACTB_HUMAN	Actin, cytoplasmic 1	74.13	108
sp|Q06210|GFPT1_HUMAN	Glutamine--fructose-6-phosphate aminotransferase [isomerizing] 1	49.79	31
sp|P09467|F16P1_HUMAN	Fructose-1,6-bisphosphatase 1	68.93	96
sp|P49588|SYAC_HUMAN	Alanine--tRNA ligase, cytoplasmic	33.88	35
sp|P68871|HBB_HUMAN	Hemoglobin subunit beta	96.60	136
sp|P00736|C1R_HUMAN	Complement C1r subcomponent	53.33	104
sp|O14841|OPLA_HUMAN	5-oxoprolinase	33.54	35

sp|P33176|KINH_HUMAN	Kinesin-1 heavy chain	28.87	29
sp|P26639|SYTC_HUMAN	Threonine--tRNA ligase, cytoplasmic	37.07	38
sp|Q14980|NUMA1_HUMAN	Nuclear mitotic apparatus protein 1	14.42	28
sp|P32754|HPPD_HUMAN	4-hydroxyphenylpyruvate dioxygenase	64.89	39
sp|P00450|CERU_HUMAN	Ceruloplasmin	27.70	35
sp|Q13200|PSMD2_HUMAN	26S proteasome non-ATPase regulatory subunit 2	41.30	31
sp|Q9P2B2|FPRP_HUMAN	Prostaglandin F2 receptor negative regulator	30.15	34
sp|P42357|HUTH_HUMAN	Histidine ammonia-lyase	58.45	52
sp|Q3LXA3|TKFC_HUMAN	Triokinase/FMN cyclase	62.61	83
sp|Q8WUM4|PDC6I_HUMAN	Programmed cell death 6-interacting protein	35.60	25
sp|P38646|GRP75_HUMAN	Stress-70 protein, mitochondrial	47.42	34
sp|P05091|ALDH2_HUMAN	Aldehyde dehydrogenase, mitochondrial	63.25	101
sp|P0DJI9|SAA2_HUMAN	Serum amyloid A-2 protein	81.97	122
sp|P04843|RPN1_HUMAN	Dolichyl-diphosphooligosaccharide--protein glycosyltransferase subunit 1	43.66	33
sp|Q00796|DHSO_HUMAN	Sorbitol dehydrogenase	86.55	151
sp|O95954|FTCD_HUMAN	Formimidoyltransferase-cyclodeaminase	57.86	61
sp|P21399|ACOC_HUMAN	Cytoplasmic aconitate hydratase	34.87	30
sp|O75116|ROCK2_HUMAN	Rho-associated protein kinase 2	19.74	28
sp|P24752|THIL_HUMAN	Acetyl-CoA acetyltransferase, mitochondrial	57.61	61
sp|P10909|CLUS_HUMAN	Clusterin	47.88	86
sp|P00439|PH4H_HUMAN	Phenylalanine-4-hydroxylase	62.61	40
sp|Q13228|SBP1_HUMAN	Selenium-binding protein 1	56.99	31
sp|P27824|CALX_HUMAN	Calnexin	44.76	40
sp|P08758|ANXA5_HUMAN	Annexin A5	75.63	44
sp|P07996|TSP1_HUMAN	Thrombospondin-1	22.48	35
sp|Q03252|LMNB2_HUMAN	Lamin-B2	44.68	36
sp|P30613|KPYR_HUMAN	Pyruvate kinase PKLR	48.95	37
sp|P01008|ANT3_HUMAN	Antithrombin-III	43.97	35
sp|Q15393|SF3B3_HUMAN	Splicing factor 3B subunit 3	23.99	28
sp|P07384|CAN1_HUMAN	Calpain-1 catalytic subunit	30.39	34
sp|O60701|UGDH_HUMAN	UDP-glucose 6-dehydrogenase	52.43	29
sp|P05166|PCCB_HUMAN	Propionyl-CoA carboxylase beta chain, mitochondrial	58.26	31
sp|O14818|PSA7_HUMAN	Proteasome subunit alpha type-7	68.15	66
sp|O60763|USO1_HUMAN	General vesicular transport factor p115	29.63	29
sp|O94979|SC31A_HUMAN	Protein transport protein Sec31A	25.00	32
sp|P68104|EF1A1_HUMAN	Elongation factor 1-alpha 1	74.46	89
sp|Q14697|GANAB_HUMAN	Neutral alpha-glucosidase AB	31.14	28
sp|P04424|ARLY_HUMAN	Argininosuccinate lyase	45.47	35
sp|P40939|ECHA_HUMAN	Trifunctional enzyme subunit alpha, mitochondrial	35.91	24
sp|P13796|PLSL_HUMAN	Plastin-2	39.71	41
sp|P31948|STIP1_HUMAN	Stress-induced-phosphoprotein 1	41.62	31
sp|P50395|GDIB_HUMAN	Rab GDP dissociation inhibitor beta	54.83	31
sp|P08603|CFAH_HUMAN	Complement factor H	35.34	53
sp|P02679|FIBG_HUMAN	Fibrinogen gamma chain	49.23	67
sp|P22413|ENPP1_HUMAN	Ectonucleotide pyrophosphatase/phosphodiesterase family member 1	33.41	30
sp|P09525|ANXA4_HUMAN	Annexin A4	65.20	47
sp|P49748|ACADV_HUMAN	Very long-chain specific acyl-CoA dehydrogenase, mitochondrial	40.15	28
sp|Q93088|BHMT1_HUMAN	Betaine--homocysteine S-methyltransferase 1	65.52	86
sp|Q99460|PSMD1_HUMAN	26S proteasome non-ATPase regulatory subunit 1	34.63	28
sp|P62258|1433E_HUMAN	14-3-3 protein epsilon	74.90	53
sp|P34896|GLYC_HUMAN	Serine hydroxymethyltransferase, cytosolic	49.90	31
sp|P06133|UD2B4_HUMAN	UDP-glucuronosyltransferase 2B4	52.84	34
sp|O75874|IDHC_HUMAN	Isocitrate dehydrogenase [NADP] cytoplasmic	57.25	75
sp|P42765|THIM_HUMAN	3-ketoacyl-CoA thiolase, mitochondrial	63.98	45

sp|Q6FI13|H2A2A_HUMAN	Histone H2A type 2-A	60.77	102
sp|P80404|GABT_HUMAN	4-aminobutyrate aminotransferase, mitochondrial	50.80	32
sp|Q6XQN6|PNCB_HUMAN	Nicotinate phosphoribosyltransferase	54.46	29
sp|P25786|PSA1_HUMAN	Proteasome subunit alpha type-1	74.90	69
sp|P51659|DHB4_HUMAN	Peroxisomal multifunctional enzyme type 2	41.44	28
sp|P78371|TCPB_HUMAN	T-complex protein 1 subunit beta	52.15	25
sp|Q14624|ITIH4_HUMAN	Inter-alpha-trypsin inhibitor heavy chain H4	26.24	34
sp|Q14117|DPYS_HUMAN	Dihydropyrimidinase	58.19	36
sp|O95831|AIFM1_HUMAN	Apoptosis-inducing factor 1, mitochondrial	38.66	31
sp|P28838|AMPL_HUMAN	Cytosol aminopeptidase	63.58	50
sp|P05556|ITB1_HUMAN	Integrin beta-1	25.19	41
sp|P17174|AATC_HUMAN	Aspartate aminotransferase, cytoplasmic	60.05	33
sp|P80723|BASP1_HUMAN	Brain acid soluble protein 1	91.63	32
sp|P27797|CALR_HUMAN	Calreticulin	55.88	87
sp|P13647|K2C5_HUMAN	Keratin, type II cytoskeletal 5	49.83	72
sp|Q15046|SYK_HUMAN	Lysine--tRNA ligase	34.67	26
sp|Q96QK1|VPS35_HUMAN	Vacuolar protein sorting-associated protein 35	30.28	24
sp|P49419|AL7A1_HUMAN	Alpha-aminoadipic semialdehyde dehydrogenase	48.61	40
sp|Q15582|BGH3_HUMAN	Transforming growth factor-beta-induced protein ig-h3	39.39	31
sp|Q14764|MVP_HUMAN	Major vault protein	28.67	23
sp|P02790|HEMO_HUMAN	Hemopexin	55.19	55
sp|Q96I99|SUCB2_HUMAN	Succinate--CoA ligase [GDP-forming] subunit beta, mitochondrial	44.91	28
sp|P49721|PSB2_HUMAN	Proteasome subunit beta type-2	82.09	47
sp|Q9UI17|M2GD_HUMAN	Dimethylglycine dehydrogenase, mitochondrial	28.87	28
sp|P08779|K1C16_HUMAN	Keratin, type I cytoskeletal 16	57.29	55
sp|Q15436|SC23A_HUMAN	Protein transport protein Sec23A	40.65	32
sp|Q9UBR1|BUP1_HUMAN	Beta-ureidopropionase	57.03	39
sp|P21980|TGM2_HUMAN	Protein-glutamine gamma-glutamyltransferase 2	39.74	38
sp|Q15084|PDIA6_HUMAN	Protein disulfide-isomerase A6	50.91	40
sp|P11940|PABP1_HUMAN	Polyadenylate-binding protein 1	31.60	20
sp|P14550|AK1A1_HUMAN	Alcohol dehydrogenase [NADP(+)]	61.85	32
sp|P61158|ARP3_HUMAN	Actin-related protein 3	61.00	37
sp|P09327|VILI_HUMAN	Villin-1	25.51	22
sp|P01031|CO5_HUMAN	Complement C5	16.41	23
sp|P23526|SAHH_HUMAN	Adenosylhomocysteinase	43.29	33
sp|P19224|UD16_HUMAN	UDP-glucuronosyltransferase 1-6	36.47	30
sp|P01871|IGHM_HUMAN	Immunoglobulin heavy constant mu	43.49	58
sp|Q03154|ACY1_HUMAN	Aminoacylase-1	62.01	26
sp|P00751|CFAB_HUMAN	Complement factor B	21.60	26
sp|P34932|HSP74_HUMAN	Heat shock 70 kDa protein 4	25.24	22
sp|P22234|PUR6_HUMAN	Multifunctional protein ADE2	44.71	41
sp|P29401|TKT_HUMAN	Transketolase	35.79	31
sp|P08238|HS90B_HUMAN	Heat shock protein HSP 90-beta	45.86	74
sp|Q02818|NUCB1_HUMAN	Nucleobindin-1	44.90	22
sp|O15144|ARPC2_HUMAN	Actin-related protein 2/3 complex subunit 2	62.33	32
sp|Q05682|CALD1_HUMAN	Caldesmon	24.72	24
sp|P40222|TXLNA_HUMAN	Alpha-taxilin	36.81	20
sp|Q9UBQ7|GRHPR_HUMAN	Glyoxylate reductase/hydroxypyruvate reductase	60.06	45
sp|Q7Z5P4|DHB13_HUMAN	17-beta-hydroxysteroid dehydrogenase 13	56.00	27
sp|P07355|ANXA2_HUMAN	Annexin A2	58.11	31
sp|O95479|G6PE_HUMAN	GDH/6PGL endoplasmic bifunctional protein	26.42	24
sp|O00748|EST2_HUMAN	Cocaine esterase	39.53	33
sp|Q08380|LG3BP_HUMAN	Galectin-3-binding protein	28.55	27
sp|Q9Y266|NUDC_HUMAN	Nuclear migration protein nudC	53.47	21
sp|Q06323|PSME1_HUMAN	Proteasome activator complex subunit 1	64.26	25
sp|P78417|GSTO1_HUMAN	Glutathione S-transferase omega-1	61.00	34

sp|P06744|G6PI_HUMAN	Glucose-6-phosphate isomerase	36.02	26
sp|P30084|ECHM_HUMAN	Enoyl-CoA hydratase, mitochondrial	65.17	58
sp|P04792|HSPB1_HUMAN	Heat shock protein beta-1	78.05	41
sp|Q00341|VIGLN_HUMAN	Vigilin	15.77	18
sp|Q96KP4|CNDP2_HUMAN	Cytosolic non-specific dipeptidase	46.11	27
sp|P19971|TYPH_HUMAN	Thymidine phosphorylase	51.04	31
sp|P00505|AATM_HUMAN	Aspartate aminotransferase, mitochondrial	48.37	27
sp|Q14554|PDIA5_HUMAN	Protein disulfide-isomerase A5	35.65	22
sp|Q16822|PCKGM_HUMAN	Phosphoenolpyruvate carboxykinase [GTP], mitochondrial	35.94	27
sp|Q99798|ACON_HUMAN	Aconitate hydratase, mitochondrial	30.77	20
sp|Q9UNZ2|NSF1C_HUMAN	NSFL1 cofactor p47	56.49	23
sp|P53618|COPB_HUMAN	Coatomer subunit beta	25.71	22
sp|Q86VP6|CAND1_HUMAN	Cullin-associated NEDD8-dissociated protein 1	18.37	18
sp|P25787|PSA2_HUMAN	Proteasome subunit alpha type-2	71.37	53
sp|P13929|ENOB_HUMAN	Beta-enolase	51.15	41
sp|P21695|GPDA_HUMAN	Glycerol-3-phosphate dehydrogenase [NAD(+)], cytoplasmic	55.01	23
sp|Q9UQE7|SMC3_HUMAN	Structural maintenance of chromosomes protein 3	21.94	22
sp|O95782|AP2A1_HUMAN	AP-2 complex subunit alpha-1	22.31	21
sp|P68371|TBB4B_HUMAN	Tubulin beta-4B chain	49.44	21
sp|P62805|H4_HUMAN	Histone H4	60.19	137
sp|P19823|ITIH2_HUMAN	Inter-alpha-trypsin inhibitor heavy chain H2	18.18	17
sp|P26641|EF1G_HUMAN	Elongation factor 1-gamma	35.24	27
sp|P28332|ADH6_HUMAN	Alcohol dehydrogenase 6	63.86	47
sp|P00338|LDHA_HUMAN	L-lactate dehydrogenase A chain	58.73	45
sp|P60900|PSA6_HUMAN	Proteasome subunit alpha type-6	65.85	45
sp|P05155|IC1_HUMAN	Plasma protease C1 inhibitor	37.40	33
sp|P49720|PSB3_HUMAN	Proteasome subunit beta type-3	61.95	55
sp|P33121|ACSL1_HUMAN	Long-chain-fatty-acid--CoA ligase 1	25.93	20
sp|Q9NQX3|GEPH_HUMAN	Gephyrin	32.88	19
sp|O43242|PSMD3_HUMAN	26S proteasome non-ATPase regulatory subunit 3	42.32	26
sp|P13010|XRCC5_HUMAN	X-ray repair cross-complementing protein 5	25.00	18
sp|P16152|CBR1_HUMAN	Carbonyl reductase [NADPH] 1	75.81	62
sp|Q14974|IMB1_HUMAN	Importin subunit beta-1	24.89	21
sp|P13797|PLST_HUMAN	Plastin-3	38.57	30
sp|P15924|DESP_HUMAN	Desmoplakin	6.72	18
sp|P08236|BGLR_HUMAN	Beta-glucuronidase	32.87	38
sp|Q08257|QOR_HUMAN	Quinone oxidoreductase	72.34	25
sp|P35241|RADI_HUMAN	Radixin	40.99	45
sp|P35520|CBS_HUMAN	Cystathionine beta-synthase	44.83	36
sp|Q14914|PTGR1_HUMAN	Prostaglandin reductase 1	55.02	30
sp|P30837|AL1B1_HUMAN	Aldehyde dehydrogenase X, mitochondrial	35.78	19
sp|Q9Y678|COPG1_HUMAN	Coatomer subunit gamma-1	21.05	22
sp|Q9HDC9|APMAP_HUMAN	Adipocyte plasma membrane-associated protein	46.88	31
sp|P54868|HMCS2_HUMAN	Hydroxymethylglutaryl-CoA synthase, mitochondrial	42.13	31
sp|P25325|THTM_HUMAN	3-mercaptopyruvate sulfurtransferase	68.35	28
sp|Q9ULD0|OGDHL_HUMAN	2-oxoglutarate dehydrogenase-like, mitochondrial	19.70	18
sp|P11712|CP2C9_HUMAN	Cytochrome P450 2C9	35.92	21
sp|P30041|PRDX6_HUMAN	Peroxiredoxin-6	68.75	42
sp|P53992|SC24C_HUMAN	Protein transport protein Sec24C	18.74	16
sp|P05362|ICAM1_HUMAN	Intercellular adhesion molecule 1	32.89	26
sp|Q01518|CAP1_HUMAN	Adenylyl cyclase-associated protein 1	54.11	29
sp|Q02790|FKBP4_HUMAN	Peptidyl-prolyl cis-trans isomerase FKBP4	47.93	24
sp|P05089|ARGI1_HUMAN	Arginase-1	63.98	42
sp|P60174|TPIS_HUMAN	Triosephosphate isomerase	71.33	40
sp|Q13561|DCTN2_HUMAN	Dynactin subunit 2	53.62	18
sp|Q99832|TCPH_HUMAN	T-complex protein 1 subunit eta	36.28	20

sp|P28066|PSA5_HUMAN	Proteasome subunit alpha type-5	66.39	47
sp|P14314|GLU2B_HUMAN	Glucosidase 2 subunit beta	30.87	41
sp|P11047|LAMC1_HUMAN	Laminin subunit gamma-1	12.93	17
sp|O43175|SERA_HUMAN	D-3-phosphoglycerate dehydrogenase	39.40	21
sp|Q86TX2|ACOT1_HUMAN	Acyl-coenzyme A thioesterase 1	55.82	19
sp|Q12906|ILF3_HUMAN	Interleukin enhancer-binding factor 3	20.02	15
sp|P09972|ALDOC_HUMAN	Fructose-bisphosphate aldolase C	58.79	26
sp|Q86YZ3|HORN_HUMAN	Hornerin	22.14	26
sp|P52209|6PGD_HUMAN	6-phosphogluconate dehydrogenase, decarboxylating	41.61	23
sp|P01011|AACT_HUMAN	Alpha-1-antichymotrypsin	44.92	45
sp|P62191|PRS4_HUMAN	26S protease regulatory subunit 4	52.95	19
sp|O00231|PSD11_HUMAN	26S proteasome non-ATPase regulatory subunit 11	49.05	21
sp|P34913|HYES_HUMAN	Bifunctional epoxide hydrolase 2	44.68	28
sp|Q00266|METK1_HUMAN	S-adenosylmethionine synthase isoform type-1	44.56	27
sp|P23284|PPIB_HUMAN	Peptidyl-prolyl cis-trans isomerase B	58.80	35
sp|P25788|PSA3_HUMAN	Proteasome subunit alpha type-3	58.43	43
sp|P07954|FUMH_HUMAN	Fumarate hydratase, mitochondrial	48.24	17
sp|P55268|LAMB2_HUMAN	Laminin subunit beta-2	9.18	17
sp|Q99536|VAT1_HUMAN	Synaptic vesicle membrane protein VAT-1 homolog	59.54	22
sp|P51572|BAP31_HUMAN	B-cell receptor-associated protein 31	42.28	18
sp|Q14152|EIF3A_HUMAN	Eukaryotic translation initiation factor 3 subunit A	17.51	19
sp|P49189|AL9A1_HUMAN	4-trimethylaminobutyraldehyde dehydrogenase	30.97	26
sp|P54136|SYRC_HUMAN	Arginine--tRNA ligase, cytoplasmic	29.24	17
sp|Q07075|AMPE_HUMAN	Glutamyl aminopeptidase	18.60	18
sp|P46940|IQGA1_HUMAN	Ras GTPase-activating-like protein IQGAP1	11.83	20
sp|P21589|5NTD_HUMAN	5'-nucleotidase	30.84	15
sp|P07339|CATD_HUMAN	Cathepsin D	40.53	31
sp|Q9Y2S2|CRYL1_HUMAN	Lambda-crystallin homolog	59.25	29
sp|P06396|GELS_HUMAN	Gelsolin	30.56	19
sp|P19827|ITIH1_HUMAN	Inter-alpha-trypsin inhibitor heavy chain H1	23.82	18
sp|P28074|PSB5_HUMAN	Proteasome subunit beta type-5	58.17	36
sp|O75083|WDR1_HUMAN	WD repeat-containing protein 1	30.20	16
sp|P11215|ITAM_HUMAN	Integrin alpha-M	15.02	16
sp|P02748|CO9_HUMAN	Complement component C9	27.91	24
sp|P20810|ICAL_HUMAN	Calpastatin	27.54	17
sp|P22307|NLTP_HUMAN	Non-specific lipid-transfer protein	30.16	16
sp|Q15008|PSMD6_HUMAN	26S proteasome non-ATPase regulatory subunit 6	35.99	15
sp|P40227|TCPZ_HUMAN	T-complex protein 1 subunit zeta	29.00	13
sp|P50440|GATM_HUMAN	Glycine amidinotransferase, mitochondrial	43.50	22
sp|P05387|RLA2_HUMAN	60S acidic ribosomal protein P2	95.65	24
sp|Q99436|PSB7_HUMAN	Proteasome subunit beta type-7	53.79	44
sp|P45954|ACDSB_HUMAN	Short/branched chain specific acyl-CoA dehydrogenase, mitochondrial	41.90	16
sp|P17516|AK1C4_HUMAN	Aldo-keto reductase family 1 member C4	45.82	22
sp|P00480|OTC_HUMAN	Ornithine carbamoyltransferase, mitochondrial	32.77	16
sp|P16662|UD2B7_HUMAN	UDP-glucuronosyltransferase 2B7	53.12	44
sp|P20618|PSB1_HUMAN	Proteasome subunit beta type-1	65.56	56
sp|Q06830|PRDX1_HUMAN	Peroxiredoxin-1	56.28	34
sp|P16435|NCPR_HUMAN	NADPH--cytochrome P450 reductase	24.67	15
sp|P10586|PTPRF_HUMAN	Receptor-type tyrosine-protein phosphatase F	9.54	14
sp|P31939|PUR9_HUMAN	Bifunctional purine biosynthesis protein PURH	28.38	14
sp|P27487|DPP4_HUMAN	Dipeptidyl peptidase 4	19.06	21
sp|P04083|ANXA1_HUMAN	Annexin A1	47.98	17
sp|P23396|RS3_HUMAN	40S ribosomal protein S3	58.44	15
sp|P08684|CP3A4_HUMAN	Cytochrome P450 3A4	40.56	18
sp|Q9Y4L1|HYOU1_HUMAN	Hypoxia up-regulated protein 1	17.82	13

sp|P50991|TCPD_HUMAN	T-complex protein 1 subunit delta	39.89	17
sp|Q68CK6|ACS2B_HUMAN	Acyl-coenzyme A synthetase ACSM2B, mitochondrial	33.45	18
sp|Q04446|GLGB_HUMAN	1,4-alpha-glucan-branching enzyme	26.21	17
sp|P69905|HBA_HUMAN	Hemoglobin subunit alpha	83.10	95
sp|P27338|AOFB_HUMAN	Amine oxidase [flavin-containing] B	34.81	20
sp|P34897|GLYM_HUMAN	Serine hydroxymethyltransferase, mitochondrial	44.25	22
sp|P25705|ATPA_HUMAN	ATP synthase subunit alpha, mitochondrial	32.91	16
sp|P45974|UBP5_HUMAN	Ubiquitin carboxyl-terminal hydrolase 5	20.75	13
sp|Q9Y6L6|SO1B1_HUMAN	Solute carrier organic anion transporter family member 1B1	22.72	23
sp|P48643|TCPE_HUMAN	T-complex protein 1 subunit epsilon	31.79	18
sp|Q9BWD1|THIC_HUMAN	Acetyl-CoA acetyltransferase, cytosolic	49.62	20
sp|P61978|HNRPK_HUMAN	Heterogeneous nuclear ribonucleoprotein K	43.41	17
sp|P55786|PSA_HUMAN	Puromycin-sensitive aminopeptidase	15.56	13
sp|P48444|COPD_HUMAN	Coatomer subunit delta	30.92	17
sp|P04075|ALDOA_HUMAN	Fructose-bisphosphate aldolase A	50.55	18
sp|P02786|TFR1_HUMAN	Transferrin receptor protein 1	21.71	15
sp|P0DP23|CALM1_HUMAN	Calmodulin-1	83.22	38
sp|Q06033|ITIH3_HUMAN	Inter-alpha-trypsin inhibitor heavy chain H3	20.11	14
sp|P12268|IMDH2_HUMAN	Inosine-5'-monophosphate dehydrogenase 2	26.65	14
sp|P54855|UDB15_HUMAN	UDP-glucuronosyltransferase 2B15	41.32	28
sp|P10635|CP2D6_HUMAN	Cytochrome P450 2D6	36.62	14
sp|P09622|DLDH_HUMAN	Dihydrolipoyl dehydrogenase, mitochondrial	35.36	22
sp|Q96N76|HUTU_HUMAN	Urocanate hydratase	24.41	19
sp|P11509|CP2A6_HUMAN	Cytochrome P450 2A6	38.46	18
sp|P30040|ERP29_HUMAN	Endoplasmic reticulum resident protein 29	65.90	30
sp|P38117|ETFB_HUMAN	Electron transfer flavoprotein subunit beta	45.49	16
sp|P43490|NAMPT_HUMAN	Nicotinamide phosphoribosyltransferase	40.73	14
sp|Q14203|DCTN1_HUMAN	Dynactin subunit 1	13.62	14
sp|Q02252|MMSA_HUMAN	Methylmalonate-semialdehyde dehydrogenase [acylating], mitochondrial	29.16	15
sp|Q00577|PURA_HUMAN	Transcriptional activator protein Pur-alpha	57.45	19
sp|P36957|ODO2_HUMAN	Dihydrolipoyllysine-residue succinyltransferase component of 2-oxoglutarate dehydrogenase complex, mitochondrial	38.19	19
sp|P25789|PSA4_HUMAN	Proteasome subunit alpha type-4	72.41	60
sp|Q9UL46|PSME2_HUMAN	Proteasome activator complex subunit 2	61.09	22
sp|P13861|KAP2_HUMAN	cAMP-dependent protein kinase type II-alpha regulatory subunit	42.82	14
sp|P30039|PBLD_HUMAN	Phenazine biosynthesis-like domain-containing protein	65.28	25
sp|P55060|XPO2_HUMAN	Exportin-2	18.43	16
sp|Q71U36|TBA1A_HUMAN	Tubulin alpha-1A chain	31.49	20
sp|Q93099|HGD_HUMAN	Homogentisate 1,2-dioxygenase	43.15	30
sp|P51570|GALK1_HUMAN	Galactokinase	37.50	15
sp|O75452|RDH16_HUMAN	Retinol dehydrogenase 16	45.74	24
sp|P61160|ARP2_HUMAN	Actin-related protein 2	43.65	24
sp|P54619|AAKG1_HUMAN	5'-AMP-activated protein kinase subunit gamma-1	54.38	14
sp|P62807|H2B1C_HUMAN	Histone H2B type 1-C/E/F/G/I	58.73	135
sp|P09110|THIK_HUMAN	3-ketoacyl-CoA thiolase, peroxisomal	41.51	15
sp|P04844|RPN2_HUMAN	Dolichyl-diphosphooligosaccharide--protein glycosyltransferase subunit 2	32.01	17
sp|Q9HD40|SPCS_HUMAN	O-phosphoseryl-tRNA(Sec) selenium transferase	35.33	13
sp|O43747|AP1G1_HUMAN	AP-1 complex subunit gamma-1	21.53	15
sp|P27169|PON1_HUMAN	Serum paraoxonase/arylesterase 1	54.93	25
sp|P60228|EIF3E_HUMAN	Eukaryotic translation initiation factor 3 subunit E	38.20	18
sp|P48449|ERG7_HUMAN	Lanosterol synthase	19.95	13
sp|P49257|LMAN1_HUMAN	Protein ERGIC-53	41.57	48
sp|P42704|LPPRC_HUMAN	Leucine-rich PPR motif-containing protein, mitochondrial	10.90	12
sp|Q02928|CP4AB_HUMAN	Cytochrome P450 4A11	38.34	17

sp|P21549|SPYA_HUMAN	Serine--pyruvate aminotransferase	47.70	27
sp|Q6NVY1|HIBCH_HUMAN	3-hydroxyisobutyryl-CoA hydrolase, mitochondrial	35.75	18
sp|P31040|SDHA_HUMAN	Succinate dehydrogenase [ubiquinone] flavoprotein subunit, mitochondrial	24.55	14
sp|P12081|SYHC_HUMAN	Histidine--tRNA ligase, cytoplasmic	28.88	13
sp|P55084|ECHB_HUMAN	Trifunctional enzyme subunit beta, mitochondrial	31.65	13
sp|Q12905|ILF2_HUMAN	Interleukin enhancer-binding factor 2	50.77	16
sp|O60271|JIP4_HUMAN	C-Jun-amino-terminal kinase-interacting protein 4	12.26	13
sp|Q16401|PSMD5_HUMAN	26S proteasome non-ATPase regulatory subunit 5	29.96	14
sp|P35998|PRS7_HUMAN	26S protease regulatory subunit 7	30.95	14
sp|P37837|TALDO_HUMAN	Transaldolase	37.39	24
sp|P50053|KHK_HUMAN	Ketohexokinase	56.04	23
sp|Q96G03|PGM2_HUMAN	Phosphoglucomutase-2	23.69	15
sp|P16930|FAAA_HUMAN	Fumarylacetoacetase	45.82	19
sp|P54920|SNAA_HUMAN	Alpha-soluble NSF attachment protein	53.56	12
sp|Q14683|SMC1A_HUMAN	Structural maintenance of chromosomes protein 1A	15.17	18
sp|P30153|2AAA_HUMAN	Serine/threonine-protein phosphatase 2A 65 kDa regulatory subunit A alpha isoform	29.71	14
sp|P0DOX5|IGG1_HUMAN	Immunoglobulin gamma-1 heavy chain	50.11	40
sp|Q13011|ECH1_HUMAN	Delta(3,5)-Delta(2,4)-dienoyl-CoA isomerase, mitochondrial	60.37	35
sp|P02538|K2C6A_HUMAN	Keratin, type II cytoskeletal 6A	53.72	84
sp|P08195|4F2_HUMAN	4F2 cell-surface antigen heavy chain	24.13	13
sp|Q9BXS5|AP1M1_HUMAN	AP-1 complex subunit mu-1	38.30	13
sp|Q16881|TRXR1_HUMAN	Thioredoxin reductase 1, cytoplasmic	18.49	13
sp|P11498|PYC_HUMAN	Pyruvate carboxylase, mitochondrial	17.40	14
sp|P01889|1B07_HUMAN	HLA class I histocompatibility antigen, B-7 alpha chain	40.06	19
sp|P18428|LBP_HUMAN	Lipopolysaccharide-binding protein	25.99	23
sp|P40926|MDHM_HUMAN	Malate dehydrogenase, mitochondrial	61.54	28
sp|P05388|RLA0_HUMAN	60S acidic ribosomal protein P0	58.04	31
sp|Q12959|DLG1_HUMAN	Disks large homolog 1	16.26	14
sp|P21333|FLNA_HUMAN	Filamin-A	10.73	25
sp|P08865|RSSA_HUMAN	40S ribosomal protein SA	44.41	15
sp|P67936|TPM4_HUMAN	Tropomyosin alpha-4 chain	45.97	20
sp|Q9Y617|SERC_HUMAN	Phosphoserine aminotransferase	40.00	17
sp|Q9Y262|EIF3L_HUMAN	Eukaryotic translation initiation factor 3 subunit L	22.16	14
sp|P30046|DOPD_HUMAN	D-dopachrome decarboxylase	83.90	25
sp|P35232|PHB_HUMAN	Prohibitin	52.94	12
sp|P00491|PNPH_HUMAN	Purine nucleoside phosphorylase	52.25	13
sp|P08473|NEP_HUMAN	Neprilysin	16.93	13
sp|P62906|RL10A_HUMAN	60S ribosomal protein L10a	43.78	15
sp|P51665|PSMD7_HUMAN	26S proteasome non-ATPase regulatory subunit 7	45.06	13
sp|O00264|PGRC1_HUMAN	Membrane-associated progesterone receptor component 1	44.62	17
sp|P07358|CO8B_HUMAN	Complement component C8 beta chain	21.32	15
sp|Q9BS26|ERP44_HUMAN	Endoplasmic reticulum resident protein 44	45.32	17
sp|Q8N0X4|CLYBL_HUMAN	Citrate lyase subunit beta-like protein, mitochondrial	51.76	19
sp|P51649|SSDH_HUMAN	Succinate-semialdehyde dehydrogenase, mitochondrial	30.28	14
sp|Q13616|CUL1_HUMAN	Cullin-1	20.36	14
sp|Q16698|DECR_HUMAN	2,4-dienoyl-CoA reductase, mitochondrial	53.73	23
sp|P22626|ROA2_HUMAN	Heterogeneous nuclear ribonucleoproteins A2/B1	37.96	17
sp|Q13093|PAFA_HUMAN	Platelet-activating factor acetylhydrolase	30.39	14
sp|Q9UQ80|PA2G4_HUMAN	Proliferation-associated protein 2G4	33.25	14
sp|Q9UHG3|PCYOX_HUMAN	Prenylcysteine oxidase 1	39.41	20
sp|Q92499|DDX1_HUMAN	ATP-dependent RNA helicase DDX1	17.97	11
sp|Q06520|ST2A1_HUMAN	Bile salt sulfotransferase	43.51	22
sp|P49589|SYCC_HUMAN	Cysteine--tRNA ligase, cytoplasmic	17.78	13
sp|P13798|ACPH_HUMAN	Acylamino-acid-releasing enzyme	17.90	12

sp|Q16836|HCDH_HUMAN	Hydroxyacyl-coenzyme A dehydrogenase, mitochondrial	54.14	19
sp|Q96I15|SCLY_HUMAN	Selenocysteine lyase	45.39	13
sp|P50225|ST1A1_HUMAN	Sulfotransferase 1A1	52.20	13
sp|Q9NVS9|PNPO_HUMAN	Pyridoxine-5'-phosphate oxidase	41.38	22
sp|P35606|COPB2_HUMAN	Coatomer subunit beta'	14.46	12
sp|O95154|ARK73_HUMAN	Aflatoxin B1 aldehyde reductase member 3	46.83	13
sp|P30086|PEBP1_HUMAN	Phosphatidylethanolamine-binding protein 1	79.68	52
sp|P02533|K1C14_HUMAN	Keratin, type I cytoskeletal 14	59.96	60
sp|Q12907|LMAN2_HUMAN	Vesicular integral-membrane protein VIP36	38.48	19
sp|P54577|SYYC_HUMAN	Tyrosine--tRNA ligase, cytoplasmic	24.81	13
sp|P09210|GSTA2_HUMAN	Glutathione S-transferase A2	48.20	38
sp|Q53GQ0|DHB12_HUMAN	Very-long-chain 3-oxoacyl-CoA reductase	41.03	12
sp|P27105|STOM_HUMAN	Erythrocyte band 7 integral membrane protein	53.47	23
sp|P00167|CYB5_HUMAN	Cytochrome b5	73.13	44
sp|P02774|VTDB_HUMAN	Vitamin D-binding protein	27.64	17
sp|Q9UKK9|NUDT5_HUMAN	ADP-sugar pyrophosphatase	57.08	16
sp|Q96NU7|HUTI_HUMAN	Probable imidazolonepropionase	36.38	14
sp|P11310|ACADM_HUMAN	Medium-chain specific acyl-CoA dehydrogenase, mitochondrial	28.50	11
sp|P49755|TMEDA_HUMAN	Transmembrane emp24 domain-containing protein 10	50.68	16
sp|Q9NQR4|NIT2_HUMAN	Omega-amidase NIT2	31.52	13
sp|P17987|TCPA_HUMAN	T-complex protein 1 subunit alpha	25.36	12
sp|P07814|SYEP_HUMAN	Bifunctional glutamate/proline--tRNA ligase	10.19	12
sp|Q13510|ASAH1_HUMAN	Acid ceramidase	34.43	15
sp|Q13884|SNTB1_HUMAN	Beta-1-syntrophin	27.32	12
sp|Q9Y2T3|GUAD_HUMAN	Guanine deaminase	27.97	13
sp|P49368|TCPG_HUMAN	T-complex protein 1 subunit gamma	29.17	12
sp|P04179|SODM_HUMAN	Superoxide dismutase [Mn], mitochondrial	47.30	19
sp|P31937|3HIDH_HUMAN	3-hydroxyisobutyrate dehydrogenase, mitochondrial	46.13	19
sp|P22760|AAAD_HUMAN	Arylacetamide deacetylase	32.58	15
sp|O94973|AP2A2_HUMAN	AP-2 complex subunit alpha-2	23.54	19
sp|P36269|GGT5_HUMAN	Gamma-glutamyltransferase 5	22.18	16
sp|P19338|NUCL_HUMAN	Nucleolin	15.07	11
sp|P11168|GTR2_HUMAN	Solute carrier family 2, facilitated glucose transporter member 2	15.84	21
sp|P02794|FRIH_HUMAN	Ferritin heavy chain	44.26	27
sp|P32455|GBP1_HUMAN	Guanylate-binding protein 1	21.79	13
sp|P14923|PLAK_HUMAN	Junction plakoglobin	20.27	13
sp|Q8WVM8|SCFD1_HUMAN	Sec1 family domain-containing protein 1	23.83	11
sp|Q99714|HCD2_HUMAN	3-hydroxyacyl-CoA dehydrogenase type-2	56.32	11
sp|P11766|ADHX_HUMAN	Alcohol dehydrogenase class-3	32.62	23
sp|P05107|ITB2_HUMAN	Integrin beta-2	14.69	11
sp|P40925|MDHC_HUMAN	Malate dehydrogenase, cytoplasmic	45.21	22
sp|O75367|H2AY_HUMAN	Core histone macro-H2A.1	34.95	21
sp|Q9UIJ7|KAD3_HUMAN	GTP:AMP phosphotransferase AK3, mitochondrial	49.34	11
sp|Q5R3I4|TTC38_HUMAN	Tetratricopeptide repeat protein 38	31.13	18
sp|Q12882|DPYD_HUMAN	Dihydropyrimidine dehydrogenase [NADP(+)]	13.66	13
sp|P18669|PGAM1_HUMAN	Phosphoglycerate mutase 1	59.84	33
sp|P06756|ITAV_HUMAN	Integrin alpha-V	15.65	13
sp|Q8NBS9|TXND5_HUMAN	Thioredoxin domain-containing protein 5	27.08	22
sp|P01903|DRA_HUMAN	HLA class II histocompatibility antigen, DR alpha chain	48.03	31
sp|P0DJI8|SAA1_HUMAN	Serum amyloid A-1 protein	80.33	131
sp|P23378|GCSP_HUMAN	Glycine dehydrogenase (decarboxylating), mitochondrial	15.10	14
sp|P07148|FABPL_HUMAN	Fatty acid-binding protein, liver	81.10	38
sp|O15372|EIF3H_HUMAN	Eukaryotic translation initiation factor 3 subunit H	39.77	14
sp|P05121|PAI1_HUMAN	Plasminogen activator inhibitor 1	43.03	16
sp|Q14103|HNRPD_HUMAN	Heterogeneous nuclear ribonucleoprotein D0	38.87	17
sp|P17980|PRS6A_HUMAN	26S protease regulatory subunit 6A	42.37	15

sp|Q16181|SEPT7_HUMAN	Septin-7	27.00	14
sp|P14866|HNRPL_HUMAN	Heterogeneous nuclear ribonucleoprotein L	32.43	14
sp|P14543|NID1_HUMAN	Nidogen-1	10.99	13
sp|P29966|MARCS_HUMAN	Myristoylated alanine-rich C-kinase substrate	46.69	11
sp|O43396|TXNL1_HUMAN	Thioredoxin-like protein 1	44.29	10
sp|Q9Y265|RUVB1_HUMAN	RuvB-like 1	28.51	11
sp|P00326|ADH1G_HUMAN	Alcohol dehydrogenase 1C	81.33	192
sp|P36955|PEDF_HUMAN	Pigment epithelium-derived factor	25.60	11
sp|P09417|DHPR_HUMAN	Dihydropteridine reductase	54.51	30
sp|P53621|COPA_HUMAN	Coatomer subunit alpha	11.44	12
sp|P10412|H14_HUMAN	Histone H1.4	24.66	29
sp|P28845|DHI1_HUMAN	Corticosteroid 11-beta-dehydrogenase isozyme 1	39.38	21
sp|Q9H4A4|AMPB_HUMAN	Aminopeptidase B	26.77	15
sp|Q6IB77|GLYAT_HUMAN	Glycine N-acyltransferase	45.61	11
sp|O94760|DDAH1_HUMAN	N(G),N(G)-dimethylarginine dimethylaminohydrolase 1	37.19	11
sp|P83111|LACTB_HUMAN	Serine beta-lactamase-like protein LACTB, mitochondrial	23.58	13
sp|P28070|PSB4_HUMAN	Proteasome subunit beta type-4	55.68	60
sp|Q13618|CUL3_HUMAN	Cullin-3	20.96	12
sp|P54578|UBP14_HUMAN	Ubiquitin carboxyl-terminal hydrolase 14	21.86	9
sp|P54819|KAD2_HUMAN	Adenylate kinase 2, mitochondrial	56.49	14
sp|Q9UN36|NDRG2_HUMAN	Protein NDRG2	49.06	13
sp|P35270|SPRE_HUMAN	Sepiapterin reductase	50.96	17
sp|P48506|GSH1_HUMAN	Glutamate--cysteine ligase catalytic subunit	15.38	12
sp|Q16775|GLO2_HUMAN	Hydroxyacylglutathione hydrolase, mitochondrial	42.86	14
sp|P52758|UK114_HUMAN	Ribonuclease UK114	74.45	19
sp|Q9Y230|RUVB2_HUMAN	RuvB-like 2	27.86	11
sp|P19440|GGT1_HUMAN	Gamma-glutamyltranspeptidase 1	23.55	11
sp|P39656|OST48_HUMAN	Dolichyl-diphosphooligosaccharide--protein glycosyltransferase 48 kDa subunit	43.20	15
sp|O95394|AGM1_HUMAN	Phosphoacetylglucosamine mutase	20.11	12
sp|P63010|AP2B1_HUMAN	AP-2 complex subunit beta	32.44	31
sp|P32929|CGL_HUMAN	Cystathionine gamma-lyase	31.36	11
sp|P02741|CRP_HUMAN	C-reactive protein	37.50	38
sp|Q9UNM6|PSD13_HUMAN	26S proteasome non-ATPase regulatory subunit 13	33.24	12
sp|Q9NSK0|KLC4_HUMAN	Kinesin light chain 4	21.16	10
sp|P26440|IVD_HUMAN	Isovaleryl-CoA dehydrogenase, mitochondrial	33.57	13
sp|P62195|PRS8_HUMAN	26S protease regulatory subunit 8	36.95	11
sp|Q9H2A2|AL8A1_HUMAN	Aldehyde dehydrogenase family 8 member A1	30.60	11
sp|Q9UBS4|DJB11_HUMAN	DnaJ homolog subfamily B member 11	25.98	9
sp|Q9Y315|DEOC_HUMAN	Deoxyribose-phosphate aldolase	46.54	13
sp|Q9BSE5|SPEB_HUMAN	Agmatinase, mitochondrial	40.34	21
sp|Q13131|AAPK1_HUMAN	5'-AMP-activated protein kinase catalytic subunit alpha-1	20.93	10
sp|P05534|1A24_HUMAN	HLA class I histocompatibility antigen, A-24 alpha chain	36.71	21
sp|P84243|H33_HUMAN	Histone H3.3	72.06	40
sp|Q9ULA0|DNPEP_HUMAN	Aspartyl aminopeptidase	29.89	15
sp|P55884|EIF3B_HUMAN	Eukaryotic translation initiation factor 3 subunit B	15.60	11
sp|Q15493|RGN_HUMAN	Regucalcin	34.11	15
sp|P01876|IGHA1_HUMAN	Immunoglobulin heavy constant alpha 1	41.64	60
sp|P62937|PPIA_HUMAN	Peptidyl-prolyl cis-trans isomerase A	70.91	28
sp|P29692|EF1D_HUMAN	Elongation factor 1-delta	41.28	22
sp|P40306|PSB10_HUMAN	Proteasome subunit beta type-10	55.31	16
sp|Q9BVK6|TMED9_HUMAN	Transmembrane emp24 domain-containing protein 9	41.70	17
sp|P55327|TPD52_HUMAN	Tumor protein D52	57.14	9
sp|P15104|GLNA_HUMAN	Glutamine synthetase	31.90	16
sp|Q16853|AOC3_HUMAN	Membrane primary amine oxidase	12.45	10
sp|O94905|ERLN2_HUMAN	Erlin-2	38.05	12

sp|P35914|HMGCL_HUMAN	Hydroxymethylglutaryl-CoA lyase, mitochondrial	44.92	16
sp|P62269|RS18_HUMAN	40S ribosomal protein S18	48.03	18
sp|Q96AE4|FUBP1_HUMAN	Far upstream element-binding protein 1	20.34	10
sp|P02792|FRIL_HUMAN	Ferritin light chain	54.86	22
sp|O60568|PLOD3_HUMAN	Procollagen-lysine,2-oxoglutarate 5-dioxygenase 3	20.05	12
sp|P28072|PSB6_HUMAN	Proteasome subunit beta type-6	61.92	28
sp|P38606|VATA_HUMAN	V-type proton ATPase catalytic subunit A	16.86	8
sp|Q7Z4W1|DCXR_HUMAN	L-xylulose reductase	65.98	34
sp|Q9BRX8|F213A_HUMAN	Redox-regulatory protein FAM213A	36.24	11
sp|Q15166|PON3_HUMAN	Serum paraoxonase/lactonase 3	43.22	19
sp|O94826|TOM70_HUMAN	Mitochondrial import receptor subunit TOM70	23.85	10
sp|P37059|DHB2_HUMAN	Estradiol 17-beta-dehydrogenase 2	34.11	11
sp|P46952|3HAO_HUMAN	3-hydroxyanthranilate 3,4-dioxygenase	37.06	12
sp|P13716|HEM2_HUMAN	Delta-aminolevulinic acid dehydratase	47.27	31
sp|P02766|TTHY_HUMAN	Transthyretin	74.83	14
sp|O75191|XYLB_HUMAN	Xylulose kinase	25.75	17
sp|O00303|EIF3F_HUMAN	Eukaryotic translation initiation factor 3 subunit F	31.65	9
sp|O00410|IPO5_HUMAN	Importin-5	12.12	10
sp|P21912|SDHB_HUMAN	Succinate dehydrogenase [ubiquinone] iron-sulfur subunit, mitochondrial	36.43	12
sp|P05186|PPBT_HUMAN	Alkaline phosphatase, tissue-nonspecific isozyme	24.43	10
sp|P13804|ETFA_HUMAN	Electron transfer flavoprotein subunit alpha, mitochondrial	49.85	13
sp|P07195|LDHB_HUMAN	L-lactate dehydrogenase B chain	39.82	12
sp|P54727|RD23B_HUMAN	UV excision repair protein RAD23 homolog B	25.67	11
sp|P01833|PIGR_HUMAN	Polymeric immunoglobulin receptor	17.28	13
sp|P51648|AL3A2_HUMAN	Fatty aldehyde dehydrogenase	27.42	17
sp|Q9HC35|EMAL4_HUMAN	Echinoderm microtubule-associated protein-like 4	9.58	10
sp|P02655|APOC2_HUMAN	Apolipoprotein C-II	78.22	19
sp|P23786|CPT2_HUMAN	Carnitine O-palmitoyltransferase 2, mitochondrial	21.12	12
sp|Q9Y5X3|SNX5_HUMAN	Sorting nexin-5	29.46	10
sp|Q9UMS4|PRP19_HUMAN	Pre-mRNA-processing factor 19	21.23	9
sp|P50995|ANX11_HUMAN	Annexin A11	23.56	13
sp|Q04828|AK1C1_HUMAN	Aldo-keto reductase family 1 member C1	39.63	23
sp|P78329|CP4F2_HUMAN	Phylloquinone omega-hydroxylase CYP4F2	20.00	12
sp|Q9NTX5|ECHD1_HUMAN	Ethylmalonyl-CoA decarboxylase	56.68	16
sp|P49591|SYSC_HUMAN	Serine--tRNA ligase, cytoplasmic	21.40	9
sp|O95497|VNN1_HUMAN	Pantetheinase	24.17	12
sp|P52888|THOP1_HUMAN	Thimet oligopeptidase	16.55	11
sp|Q9Y696|CLIC4_HUMAN	Chloride intracellular channel protein 4	50.20	11
sp|P20073|ANXA7_HUMAN	Annexin A7	21.52	10
sp|Q14749|GNMT_HUMAN	Glycine N-methyltransferase	36.27	20
sp|P32119|PRDX2_HUMAN	Peroxiredoxin-2	37.37	17
sp|P36776|LONM_HUMAN	Lon protease homolog, mitochondrial	13.24	12
sp|Q969P0|IGSF8_HUMAN	Immunoglobulin superfamily member 8	17.94	10
sp|P35613|BASI_HUMAN	Basigin	21.56	9
sp|Q9BT78|CSN4_HUMAN	COP9 signalosome complex subunit 4	27.59	9
sp|P21281|VATB2_HUMAN	V-type proton ATPase subunit B, brain isoform	22.70	10
sp|O60506|HNRPQ_HUMAN	Heterogeneous nuclear ribonucleoprotein Q	17.82	9
sp|Q14240|IF4A2_HUMAN	Eukaryotic initiation factor 4A-II	23.83	10
sp|O75822|EIF3J_HUMAN	Eukaryotic translation initiation factor 3 subunit J	31.78	10
sp|Q08830|FGL1_HUMAN	Fibrinogen-like protein 1	38.78	21
sp|Q96IU4|ABHEB_HUMAN	Protein ABHD14B	47.62	11
sp|P63104|1433Z_HUMAN	14-3-3 protein zeta/delta	63.67	27
sp|P07686|HEXB_HUMAN	Beta-hexosaminidase subunit beta	14.03	9
sp|Q96C11|FGGY_HUMAN	FGGY carbohydrate kinase domain-containing protein	20.69	9
sp|P0DOX7|IGK_HUMAN	Immunoglobulin kappa light chain	54.67	55

sp|P27695|APEX1_HUMAN	DNA-(apurinic or apyrimidinic site) lyase	34.59	14
sp|O00499|BIN1_HUMAN	Myc box-dependent-interacting protein 1	21.08	10
sp|O75533|SF3B1_HUMAN	Splicing factor 3B subunit 1	8.05	11
sp|P13671|CO6_HUMAN	Complement component C6	10.60	13
sp|P08648|ITA5_HUMAN	Integrin alpha-5	15.92	15
sp|Q08211|DHX9_HUMAN	ATP-dependent RNA helicase A	9.76	10
sp|Q13825|AUHM_HUMAN	Methylglutaconyl-CoA hydratase, mitochondrial	33.33	12
sp|Q4G0N4|NAKD2_HUMAN	NAD kinase 2, mitochondrial	21.49	9
sp|P02652|APOA2_HUMAN	Apolipoprotein A-II	62.00	35
sp|Q13162|PRDX4_HUMAN	Peroxiredoxin-4	53.87	23
sp|Q96C23|GALM_HUMAN	Aldose 1-epimerase	34.21	9
sp|P51690|ARSE_HUMAN	Arylsulfatase E	21.39	12
sp|P36537|UDB10_HUMAN	UDP-glucuronosyltransferase 2B10	42.05	29
sp|Q15907|RB11B_HUMAN	Ras-related protein Rab-11B	38.99	8
sp|P02656|APOC3_HUMAN	Apolipoprotein C-III	75.76	47
sp|Q9UHD8|SEPT9_HUMAN	Septin-9	14.51	8
sp|Q96HR9|REEP6_HUMAN	Receptor expression-enhancing protein 6	33.18	30
sp|O94903|PROSC_HUMAN	Proline synthase co-transcribed bacterial homolog protein	45.09	10
sp|P16219|ACADS_HUMAN	Short-chain specific acyl-CoA dehydrogenase, mitochondrial	32.28	9
sp|P59998|ARPC4_HUMAN	Actin-related protein 2/3 complex subunit 4	55.36	17
sp|P07737|PROF1_HUMAN	Profilin-1	70.00	15
sp|P24298|ALAT1_HUMAN	Alanine aminotransferase 1	21.37	9
sp|O60826|CCD22_HUMAN	Coiled-coil domain-containing protein 22	22.17	10
sp|P55263|ADK_HUMAN	Adenosine kinase	28.45	9
sp|Q9NPD5|SO1B3_HUMAN	Solute carrier organic anion transporter family member 1B3	18.52	21
sp|Q02218|ODO1_HUMAN	2-oxoglutarate dehydrogenase, mitochondrial	20.33	18
sp|P13760|2B14_HUMAN	HLA class II histocompatibility antigen, DRB1-4 beta chain	39.85	26
sp|Q96JB5|CK5P3_HUMAN	CDK5 regulatory subunit-associated protein 3	21.54	11
sp|P56470|LEG4_HUMAN	Galectin-4	36.22	14
sp|P51687|SUOX_HUMAN	Sulfite oxidase, mitochondrial	18.17	8
sp|Q9NVA2|SEP11_HUMAN	Septin-11	24.01	13
sp|P43686|PRS6B_HUMAN	26S protease regulatory subunit 6B	36.84	10
sp|Q99623|PHB2_HUMAN	Prohibitin-2	32.11	8
sp|Q15293|RCN1_HUMAN	Reticulocalbin-1	33.23	9
sp|P05455|LA_HUMAN	Lupus La protein	25.49	10
sp|P07357|CO8A_HUMAN	Complement component C8 alpha chain	11.99	8
sp|P47755|CAZA2_HUMAN	F-actin-capping protein subunit alpha-2	44.41	13
sp|O15173|PGRC2_HUMAN	Membrane-associated progesterone receptor component 2	46.64	10
sp|Q9Y5C1|ANGL3_HUMAN	Angiopoietin-related protein 3	21.96	11
sp|Q9BZZ5|API5_HUMAN	Apoptosis inhibitor 5	15.65	8
sp|Q32MZ4|LRRF1_HUMAN	Leucine-rich repeat flightless-interacting protein 1	13.12	8
sp|P07910|HNRPC_HUMAN	Heterogeneous nuclear ribonucleoproteins C1/C2	24.51	9
sp|P27348|1433T_HUMAN	14-3-3 protein theta	53.06	20
sp|P15311|EZRI_HUMAN	Ezrin	35.49	42
sp|P16070|CD44_HUMAN	CD44 antigen	8.22	13
sp|Q13838|DX39B_HUMAN	Spliceosome RNA helicase DDX39B	22.20	10
sp|O95486|SC24A_HUMAN	Protein transport protein Sec24A	9.42	8
sp|Q96HC4|PDLI5_HUMAN	PDZ and LIM domain protein 5	17.79	8
sp|P23528|COF1_HUMAN	Cofilin-1	69.28	13
sp|P15531|NDKA_HUMAN	Nucleoside diphosphate kinase A	57.89	11
sp|P47897|SYQ_HUMAN	Glutamine--tRNA ligase	12.39	8
sp|P11226|MBL2_HUMAN	Mannose-binding protein C	30.24	11
sp|Q96AG4|LRC59_HUMAN	Leucine-rich repeat-containing protein 59	31.60	11
sp|P02743|SAMP_HUMAN	Serum amyloid P-component	30.04	16
sp|P30085|KCY_HUMAN	UMP-CMP kinase	47.45	8
sp|Q14108|SCRB2_HUMAN	Lysosome membrane protein 2	20.92	12

sp|P22897|MRC1_HUMAN	Macrophage mannose receptor 1	7.97	13
sp|O95336|6PGL_HUMAN	6-phosphogluconolactonase	48.06	8
sp|P30566|PUR8_HUMAN	Adenylosuccinate lyase	17.56	7
sp|P46926|GNPI1_HUMAN	Glucosamine-6-phosphate isomerase 1	37.02	15
sp|Q96DG6|CMBL_HUMAN	Carboxymethylenebutenolidase homolog	45.31	12
sp|Q9P0Z9|SOX_HUMAN	Peroxisomal sarcosine oxidase	29.23	11
sp|P61604|CH10_HUMAN	10 kDa heat shock protein, mitochondrial	61.76	11
sp|P22310|UD14_HUMAN	UDP-glucuronosyltransferase 1-4	35.58	31
sp|O75153|CLU_HUMAN	Clustered mitochondria protein homolog	8.25	9
sp|P37802|TAGL2_HUMAN	Transgelin-2	46.73	9
sp|O00487|PSDE_HUMAN	26S proteasome non-ATPase regulatory subunit 14	52.26	10
sp|P28331|NDUS1_HUMAN	NADH-ubiquinone oxidoreductase 75 kDa subunit, mitochondrial	15.68	8
sp|Q9HB71|CYBP_HUMAN	Calcyclin-binding protein	49.12	9
sp|P01130|LDLR_HUMAN	Low-density lipoprotein receptor	10.00	7
sp|Q9UL18|AGO1_HUMAN	Protein argonaute-1	13.65	9
sp|O95342|ABCBB_HUMAN	Bile salt export pump	7.42	9
sp|P30711|GSTT1_HUMAN	Glutathione S-transferase theta-1	37.92	11
sp|Q9NQC3|RTN4_HUMAN	Reticulon-4	7.80	26
sp|Q9Y2B0|CNPY2_HUMAN	Protein canopy homolog 2	53.30	11
sp|O94855|SC24D_HUMAN	Protein transport protein Sec24D	8.53	10
sp|P00918|CAH2_HUMAN	Carbonic anhydrase 2	33.46	11
sp|P30042|ES1_HUMAN	ES1 protein homolog, mitochondrial	36.57	8
sp|P19105|ML12A_HUMAN	Myosin regulatory light chain 12A	50.88	8
sp|P84077|ARF1_HUMAN	ADP-ribosylation factor 1	53.59	11
sp|Q969X5|ERGI1_HUMAN	Endoplasmic reticulum-Golgi intermediate compartment protein 1	30.34	7
sp|P28065|PSB9_HUMAN	Proteasome subunit beta type-9	42.47	14
sp|P47756|CAPZB_HUMAN	F-actin-capping protein subunit beta	32.13	12
sp|P08571|CD14_HUMAN	Monocyte differentiation antigen CD14	28.27	8
sp|O00299|CLIC1_HUMAN	Chloride intracellular channel protein 1	41.08	10
sp|P80303|NUCB2_HUMAN	Nucleobindin-2	25.00	10
sp|P07741|APT_HUMAN	Adenine phosphoribosyltransferase	41.67	9
sp|P07602|SAP_HUMAN	Prosaposin	22.90	16
sp|P10620|MGST1_HUMAN	Microsomal glutathione S-transferase 1	69.68	22
sp|P53597|SUCA_HUMAN	Succinate--CoA ligase [ADP/GDP-forming] subunit alpha, mitochondrial	30.64	10
sp|Q9Y2P5|S27A5_HUMAN	Bile acyl-CoA synthetase	16.81	12
sp|P30043|BLVRB_HUMAN	Flavin reductase (NADPH)	56.31	20
sp|P07305|H10_HUMAN	Histone H1.0	28.35	21
sp|Q16762|THTR_HUMAN	Thiosulfate sulfurtransferase	37.04	11
sp|O43776|SYNC_HUMAN	Asparagine--tRNA ligase, cytoplasmic	15.69	7
sp|Q16543|CDC37_HUMAN	Hsp90 co-chaperone Cdc37	20.63	8
sp|P28062|PSB8_HUMAN	Proteasome subunit beta type-8	52.54	28
sp|O60256|KPRB_HUMAN	Phosphoribosyl pyrophosphate synthase-associated protein 2	28.18	7
sp|P53396|ACLY_HUMAN	ATP-citrate synthase	8.17	9
sp|P00492|HPRT_HUMAN	Hypoxanthine-guanine phosphoribosyltransferase	38.07	7
sp|P48556|PSMD8_HUMAN	26S proteasome non-ATPase regulatory subunit 8	20.86	11
sp|O60664|PLIN3_HUMAN	Perilipin-3	24.65	8
sp|P61247|RS3A_HUMAN	40S ribosomal protein S3a	40.15	11
sp|Q7L2H7|EIF3M_HUMAN	Eukaryotic translation initiation factor 3 subunit M	27.54	7
sp|P62333|PRS10_HUMAN	26S protease regulatory subunit 10B	28.53	9
sp|P41250|GARS_HUMAN	Glycine--tRNA ligase	12.31	9
sp|Q96HS1|PGAM5_HUMAN	Serine/threonine-protein phosphatase PGAM5, mitochondrial	25.26	7
sp|P62249|RS16_HUMAN	40S ribosomal protein S16	42.47	10
sp|Q13464|ROCK1_HUMAN	Rho-associated protein kinase 1	9.45	12
sp|O00560|SDCB1_HUMAN	Syntenin-1	45.64	10
sp|Q9HC38|GLOD4_HUMAN	Glyoxalase domain-containing protein 4	25.56	8

sp|P09874|PARP1_HUMAN	Poly [ADP-ribose] polymerase 1	11.83	9
sp|A6ND91|ASPD_HUMAN	Putative L-aspartate dehydrogenase	31.45	7
sp|O43488|ARK72_HUMAN	Aflatoxin B1 aldehyde reductase member 2	25.63	9
sp|P09601|HMOX1_HUMAN	Heme oxygenase 1	40.97	8
sp|P30038|AL4A1_HUMAN	Delta-1-pyrroline-5-carboxylate dehydrogenase, mitochondrial	14.56	7
sp|P62081|RS7_HUMAN	40S ribosomal protein S7	52.06	9
sp|Q14165|MLEC_HUMAN	Malectin	38.01	9
sp|P06753|TPM3_HUMAN	Tropomyosin alpha-3 chain	31.58	14
sp|Q9Y624|JAM1_HUMAN	Junctional adhesion molecule A	30.10	8
sp|P49773|HINT1_HUMAN	Histidine triad nucleotide-binding protein 1	63.49	11
sp|Q9HCC0|MCCB_HUMAN	Methylcrotonoyl-CoA carboxylase beta chain, mitochondrial	15.81	7
sp|Q9H2U2|IPYR2_HUMAN	Inorganic pyrophosphatase 2, mitochondrial	25.75	7
sp|P31431|SDC4_HUMAN	Syndecan-4	28.79	13
sp|P10768|ESTD_HUMAN	S-formylglutathione hydrolase	30.85	9
sp|O75131|CPNE3_HUMAN	Copine-3	15.83	10
sp|P48147|PPCE_HUMAN	Prolyl endopeptidase	17.89	10
sp|Q9BPW8|NIPS1_HUMAN	Protein NipSnap homolog 1	35.56	15
sp|P16401|H15_HUMAN	Histone H1.5	26.11	8
sp|Q92734|TFG_HUMAN	Protein TFG	33.75	10
sp|P14780|MMP9_HUMAN	Matrix metalloproteinase-9	11.17	9
sp|P16083|NQO2_HUMAN	Ribosyldihydronicotinamide dehydrogenase [quinone]	41.13	7
sp|Q08AM6|VAC14_HUMAN	Protein VAC14 homolog	13.04	8
sp|Q9UNF0|PACN2_HUMAN	Protein kinase C and casein kinase substrate in neurons protein 2	14.20	7
sp|Q86YB7|ECHD2_HUMAN	Enoyl-CoA hydratase domain-containing protein 2, mitochondrial	40.07	10
sp|P30044|PRDX5_HUMAN	Peroxiredoxin-5, mitochondrial	36.92	9
sp|Q9Y295|DRG1_HUMAN	Developmentally-regulated GTP-binding protein 1	21.25	6
sp|P61981|1433G_HUMAN	14-3-3 protein gamma	46.56	23
sp|P04899|GNAI2_HUMAN	Guanine nucleotide-binding protein G(i) subunit alpha-2	32.96	9
sp|P11717|MPRI_HUMAN	Cation-independent mannose-6-phosphate receptor	3.09	7
sp|Q96CX2|KCD12_HUMAN	BTB/POZ domain-containing protein KCTD12	24.92	7
sp|O14975|S27A2_HUMAN	Very long-chain acyl-CoA synthetase	14.19	8
sp|P04632|CPNS1_HUMAN	Calpain small subunit 1	59.70	19
sp|Q8IZ83|A16A1_HUMAN	Aldehyde dehydrogenase family 16 member A1	16.46	9
sp|P30622|CLIP1_HUMAN	CAP-Gly domain-containing linker protein 1	5.98	8
sp|P30048|PRDX3_HUMAN	Thioredoxin-dependent peroxide reductase, mitochondrial	35.16	10
sp|P06702|S10A9_HUMAN	Protein S100-A9	81.58	18
sp|O15245|S22A1_HUMAN	Solute carrier family 22 member 1	11.19	8
sp|P07858|CATB_HUMAN	Cathepsin B	26.55	9
sp|P35858|ALS_HUMAN	Insulin-like growth factor-binding protein complex acid labile subunit	13.22	7
sp|Q96HE7|ERO1A_HUMAN	ERO1-like protein alpha	22.01	10
sp|O43399|TPD54_HUMAN	Tumor protein D54	37.38	6
sp|Q86UE4|LYRIC_HUMAN	Protein LYRIC	11.51	6
sp|O00232|PSD12_HUMAN	26S proteasome non-ATPase regulatory subunit 12	24.78	10
sp|P30740|ILEU_HUMAN	Leukocyte elastase inhibitor	29.29	10
sp|Q8NHM4|TRY6_HUMAN	Putative trypsin-6	27.53	150
sp|O15145|ARPC3_HUMAN	Actin-related protein 2/3 complex subunit 3	53.37	9
sp|P03951|FA11_HUMAN	Coagulation factor XI	9.28	6
sp|P05026|AT1B1_HUMAN	Sodium/potassium-transporting ATPase subunit beta-1	36.96	12
sp|P40261|NNMT_HUMAN	Nicotinamide N-methyltransferase	30.68	11
sp|Q9P035|HACD3_HUMAN	Very-long-chain (3R)-3-hydroxyacyl-CoA dehydratase 3	18.51	7
sp|P62263|RS14_HUMAN	40S ribosomal protein S14	38.41	6
sp|P00390|GSHR_HUMAN	Glutathione reductase, mitochondrial	22.22	8
sp|P60660|MYL6_HUMAN	Myosin light polypeptide 6	58.28	11
sp|Q969H8|MYDGF_HUMAN	Myeloid-derived growth factor	43.93	10
sp|Q9P0L0|VAPA_HUMAN	Vesicle-associated membrane protein-associated protein A	22.49	6

sp|P52565|GDIR1_HUMAN	Rho GDP-dissociation inhibitor 1	41.18	6
sp|P53999|TCP4_HUMAN	Activated RNA polymerase II transcriptional coactivator p15	49.61	9
sp|P05181|CP2E1_HUMAN	Cytochrome P450 2E1	16.84	9
sp|Q92841|DDX17_HUMAN	Probable ATP-dependent RNA helicase DDX17	10.15	7
sp|Q9NTK5|OLA1_HUMAN	Obg-like ATPase 1	18.94	7
sp|Q96DC8|ECHD3_HUMAN	Enoyl-CoA hydratase domain-containing protein 3, mitochondrial	34.32	9
sp|Q13310|PABP4_HUMAN	Polyadenylate-binding protein 4	17.24	11
sp|P12429|ANXA3_HUMAN	Annexin A3	23.53	7
sp|O43464|HTRA2_HUMAN	Serine protease HTRA2, mitochondrial	17.90	6
sp|P09429|HMGB1_HUMAN	High mobility group protein B1	24.65	6
sp|Q12904|AIMP1_HUMAN	Aminoacyl tRNA synthase complex-interacting multifunctional protein 1	26.60	7
sp|P51149|RAB7A_HUMAN	Ras-related protein Rab-7a	38.16	10
sp|Q9UKS6|PACN3_HUMAN	Protein kinase C and casein kinase substrate in neurons protein 3	19.58	8
sp|Q9BYT8|NEUL_HUMAN	Neurolysin, mitochondrial	13.49	9
sp|P53985|MOT1_HUMAN	Monocarboxylate transporter 1	10.80	12
sp|O43390|HNRPR_HUMAN	Heterogeneous nuclear ribonucleoprotein R	17.38	9
sp|Q99447|PCY2_HUMAN	Ethanolamine-phosphate cytidylyltransferase	20.05	9
sp|P22061|PIMT_HUMAN	Protein-L-isoaspartate(D-aspartate) O-methyltransferase	45.81	6
sp|Q9H3G5|CPVL_HUMAN	Probable serine carboxypeptidase CPVL	13.24	6
sp|P00568|KAD1_HUMAN	Adenylate kinase isoenzyme 1	33.51	6
sp|Q9NRV9|HEBP1_HUMAN	Heme-binding protein 1	45.50	6
sp|Q15459|SF3A1_HUMAN	Splicing factor 3A subunit 1	11.85	7
sp|Q08378|GOGA3_HUMAN	Golgin subfamily A member 3	4.87	6
sp|P46777|RL5_HUMAN	60S ribosomal protein L5	23.57	7
sp|Q15631|TSN_HUMAN	Translin	31.14	6
sp|P09960|LKHA4_HUMAN	Leukotriene A-4 hydrolase	18.99	9
sp|P07306|ASGR1_HUMAN	Asialoglycoprotein receptor 1	22.34	10
sp|Q9NY33|DPP3_HUMAN	Dipeptidyl peptidase 3	8.82	5
sp|P33261|CP2CJ_HUMAN	Cytochrome P450 2C19	33.06	21
sp|A0MZ66|SHOT1_HUMAN	Shootin-1	10.78	6
sp|P61106|RAB14_HUMAN	Ras-related protein Rab-14	36.28	9
sp|Q99497|PARK7_HUMAN	Protein DJ-1	67.72	16
sp|O43741|AAKB2_HUMAN	5'-AMP-activated protein kinase subunit beta-2	27.21	6
sp|Q99685|MGLL_HUMAN	Monoglyceride lipase	27.39	6
sp|Q14254|FLOT2_HUMAN	Flotillin-2	17.99	7
sp|Q9H0W9|CK054_HUMAN	Ester hydrolase C11orf54	32.38	13
sp|Q9NQW7|XPP1_HUMAN	Xaa-Pro aminopeptidase 1	12.68	6
sp|P38159|RBMX_HUMAN	RNA-binding motif protein, X chromosome	17.14	7
sp|Q7L5N1|CSN6_HUMAN	COP9 signalosome complex subunit 6	24.77	6
sp|Q16719|KYNU_HUMAN	Kynureninase	18.28	6
sp|Q92954|PRG4_HUMAN	Proteoglycan 4	5.77	8
sp|Q9BRF8|CPPED_HUMAN	Serine/threonine-protein phosphatase CPPED1	19.11	6
sp|Q04917|1433F_HUMAN	14-3-3 protein eta	46.75	19
sp|Q02750|MP2K1_HUMAN	Dual specificity mitogen-activated protein kinase kinase 1	17.56	6
sp|Q8WUY1|THEM6_HUMAN	Protein THEM6	35.58	9
sp|Q9ULX7|CAH14_HUMAN	Carbonic anhydrase 14	20.18	7
sp|Q9NY15|STAB1_HUMAN	Stabilin-1	4.20	9
sp|P21796|VDAC1_HUMAN	Voltage-dependent anion-selective channel protein 1	26.15	8
sp|Q92747|ARC1A_HUMAN	Actin-related protein 2/3 complex subunit 1A	23.78	9
sp|P05067|A4_HUMAN	Amyloid beta A4 protein	9.48	7
sp|Q13098|CSN1_HUMAN	COP9 signalosome complex subunit 1	14.46	7
sp|Q15005|SPCS2_HUMAN	Signal peptidase complex subunit 2	28.32	6
sp|Q9UJM8|HAOX1_HUMAN	Hydroxyacid oxidase 1	27.84	7
sp|Q92820|GGH_HUMAN	Gamma-glutamyl hydrolase	24.53	7
sp|P54802|ANAG_HUMAN	Alpha-N-acetylglucosaminidase	11.31	5

sp|P52907|CAZA1_HUMAN	F-actin-capping protein subunit alpha-1	45.45	12
sp|P48047|ATPO_HUMAN	ATP synthase subunit O, mitochondrial	44.13	7
sp|Q14376|GALE_HUMAN	UDP-glucose 4-epimerase	27.30	7
sp|Q7Z7H5|TMED4_HUMAN	Transmembrane emp24 domain-containing protein 4	39.21	10
sp|Q13620|CUL4B_HUMAN	Cullin-4B	9.20	8
sp|Q9NZL9|MAT2B_HUMAN	Methionine adenosyltransferase 2 subunit beta	22.75	6
sp|P51148|RAB5C_HUMAN	Ras-related protein Rab-5C	47.22	9
sp|P46781|RS9_HUMAN	40S ribosomal protein S9	31.96	7
sp|O14874|BCKD_HUMAN	[3-methyl-2-oxobutanoate dehydrogenase [lipoamide]] kinase, mitochondrial	33.25	10
sp|P62851|RS25_HUMAN	40S ribosomal protein S25	44.80	8
sp|Q9UJS0|CMC2_HUMAN	Calcium-binding mitochondrial carrier protein Aralar2	11.70	6
sp|Q12797|ASPH_HUMAN	Aspartyl/asparaginyl beta-hydroxylase	12.27	8
sp|Q15274|NADC_HUMAN	Nicotinate-nucleotide pyrophosphorylase [carboxylating]	23.23	6
sp|Q13740|CD166_HUMAN	CD166 antigen	11.66	7
sp|Q13423|NNTM_HUMAN	NAD(P) transhydrogenase, mitochondrial	6.35	6
sp|P00747|PLMN_HUMAN	Plasminogen	11.73	9
sp|P02763|A1AG1_HUMAN	Alpha-1-acid glycoprotein 1	43.78	11
sp|Q96HY6|DDRGK_HUMAN	DDRGK domain-containing protein 1	25.48	6
sp|Q00059|TFAM_HUMAN	Transcription factor A, mitochondrial	25.61	7
sp|Q13724|MOGS_HUMAN	Mannosyl-oligosaccharide glucosidase	9.56	6
sp|O75936|BODG_HUMAN	Gamma-butyrobetaine dioxygenase	17.05	7
sp|P55036|PSMD4_HUMAN	26S proteasome non-ATPase regulatory subunit 4	18.04	6
sp|P07360|CO8G_HUMAN	Complement component C8 gamma chain	44.55	8
sp|O75396|SC22B_HUMAN	Vesicle-trafficking protein SEC22b	34.42	6
sp|Q92696|PGTA_HUMAN	Geranylgeranyl transferase type-2 subunit alpha	14.11	7
sp|Q96CN7|ISOC1_HUMAN	Isochorismatase domain-containing protein 1	28.86	6
sp|Q9BUT1|BDH2_HUMAN	3-hydroxybutyrate dehydrogenase type 2	36.33	8
sp|P01019|ANGT_HUMAN	Angiotensinogen	16.29	8
sp|P35558|PCKGC_HUMAN	Phosphoenolpyruvate carboxykinase, cytosolic [GTP]	17.04	11
sp|P16671|CD36_HUMAN	Platelet glycoprotein 4	8.69	7
sp|Q16134|ETFD_HUMAN	Electron transfer flavoprotein-ubiquinone oxidoreductase, mitochondrial	12.32	8
sp|O76054|S14L2_HUMAN	SEC14-like protein 2	24.07	8
sp|P07327|ADH1A_HUMAN	Alcohol dehydrogenase 1A	77.07	221
sp|P11279|LAMP1_HUMAN	Lysosome-associated membrane glycoprotein 1	14.63	10
sp|P09661|RU2A_HUMAN	U2 small nuclear ribonucleoprotein A'	21.18	6
sp|Q9Y3A5|SBDS_HUMAN	Ribosome maturation protein SBDS	34.80	8
sp|Q96CW1|AP2M1_HUMAN	AP-2 complex subunit mu	19.31	8
sp|O43895|XPP2_HUMAN	Xaa-Pro aminopeptidase 2	9.50	8
sp|Q9UBW8|CSN7A_HUMAN	COP9 signalosome complex subunit 7a	28.36	6
sp|P26885|FKBP2_HUMAN	Peptidyl-prolyl cis-trans isomerase FKBP2	47.89	8
sp|Q92506|DHB8_HUMAN	Estradiol 17-beta-dehydrogenase 8	27.97	7
sp|Q15113|PCOC1_HUMAN	Procollagen C-endopeptidase enhancer 1	26.50	8
sp|P16615|AT2A2_HUMAN	Sarcoplasmic/endoplasmic reticulum calcium ATPase 2	7.29	6
sp|P30626|SORCN_HUMAN	Sorcin	34.34	8
sp|O14756|H17B6_HUMAN	17-beta-hydroxysteroid dehydrogenase type 6	26.81	9
sp|Q9HAW9|UD18_HUMAN	UDP-glucuronosyltransferase 1-8	25.85	21
sp|P0CG47|UBB_HUMAN	Polyubiquitin-B	77.29	20
sp|P08708|RS17_HUMAN	40S ribosomal protein S17	56.30	6
sp|P35813|PPM1A_HUMAN	Protein phosphatase 1A	15.71	5
sp|P14324|FPPS_HUMAN	Farnesyl pyrophosphate synthase	14.80	6
sp|P22102|PUR2_HUMAN	Trifunctional purine biosynthetic protein adenosine-3	7.23	5
sp|Q92597|NDRG1_HUMAN	Protein NDRG1	22.34	5
sp|Q15043|S39AE_HUMAN	Zinc transporter ZIP14	13.01	9
sp|P61088|UBE2N_HUMAN	Ubiquitin-conjugating enzyme E2 N	42.11	7

sp|P61923|COPZ1_HUMAN	Coatomer subunit zeta-1	33.90	5
sp|O43708|MAAI_HUMAN	Maleylacetoacetate isomerase	29.63	6
sp|Q96EY8|MMAB_HUMAN	Cob(I)yrinic acid a,c-diamide adenosyltransferase, mitochondrial	28.80	6
sp|P62277|RS13_HUMAN	40S ribosomal protein S13	33.11	6
sp|Q01581|HMCS1_HUMAN	Hydroxymethylglutaryl-CoA synthase, cytoplasmic	21.15	8
sp|O15488|GLYG2_HUMAN	Glycogenin-2	11.38	6
sp|P20742|PZP_HUMAN	Pregnancy zone protein	9.11	14
sp|Q9H7Z7|PGES2_HUMAN	Prostaglandin E synthase 2	16.45	5
sp|Q96LJ7|DHRS1_HUMAN	Dehydrogenase/reductase SDR family member 1	19.49	5
sp|P05177|CP1A2_HUMAN	Cytochrome P450 1A2	14.95	6
sp|P15428|PGDH_HUMAN	15-hydroxyprostaglandin dehydrogenase [NAD(+)]	23.31	5
sp|Q02809|PLOD1_HUMAN	Procollagen-lysine,2-oxoglutarate 5-dioxygenase 1	14.03	7
sp|Q08426|ECHP_HUMAN	Peroxisomal bifunctional enzyme	13.69	8
sp|P36543|VATE1_HUMAN	V-type proton ATPase subunit E 1	37.61	8
sp|P09651|ROA1_HUMAN	Heterogeneous nuclear ribonucleoprotein A1	19.89	8
sp|O95210|STBD1_HUMAN	Starch-binding domain-containing protein 1	24.58	9
sp|Q9UKG1|DP13A_HUMAN	DCC-interacting protein 13-alpha	10.86	6
sp|Q13617|CUL2_HUMAN	Cullin-2	13.83	9
sp|Q15019|SEPT2_HUMAN	Septin-2	28.81	9
sp|P02750|A2GL_HUMAN	Leucine-rich alpha-2-glycoprotein	25.94	7
sp|O00764|PDXK_HUMAN	Pyridoxal kinase	24.04	5
sp|P09211|GSTP1_HUMAN	Glutathione S-transferase P	40.00	7
sp|O43598|DNPH1_HUMAN	2'-deoxynucleoside 5'-phosphate N-hydrolase 1	47.70	7
sp|P46977|STT3A_HUMAN	Dolichyl-diphosphooligosaccharide--protein glycosyltransferase subunit STT3A	8.37	6
sp|Q9Y3A6|TMED5_HUMAN	Transmembrane emp24 domain-containing protein 5	28.82	7
sp|O14791|APOL1_HUMAN	Apolipoprotein L1	13.57	6
sp|Q8IVM8|S22A9_HUMAN	Solute carrier family 22 member 9	8.14	6
sp|P51857|AK1D1_HUMAN	3-oxo-5-beta-steroid 4-dehydrogenase	23.62	8
sp|P37235|HPCL1_HUMAN	Hippocalcin-like protein 1	35.23	7
sp|Q86X76|NIT1_HUMAN	Nitrilase homolog 1	27.83	8
sp|P62701|RS4X_HUMAN	40S ribosomal protein S4, X isoform	25.48	7
sp|Q92542|NICA_HUMAN	Nicastrin	7.33	5
sp|P19013|K2C4_HUMAN	Keratin, type II cytoskeletal 4	16.67	24
sp|O95865|DDAH2_HUMAN	N(G),N(G)-dimethylarginine dimethylaminohydrolase 2	29.12	6
sp|Q7Z6Z7|HUWE1_HUMAN	E3 ubiquitin-protein ligase HUWE1	2.29	7
sp|P09493|TPM1_HUMAN	Tropomyosin alpha-1 chain	31.34	15
sp|P11387|TOP1_HUMAN	DNA topoisomerase 1	7.71	5
sp|A6NDB9|PALM3_HUMAN	Paralemmin-3	8.32	5
sp|P62424|RL7A_HUMAN	60S ribosomal protein L7a	14.29	5
sp|P31153|METK2_HUMAN	S-adenosylmethionine synthase isoform type-2	24.05	9
sp|P05198|IF2A_HUMAN	Eukaryotic translation initiation factor 2 subunit 1	17.46	5
sp|Q9Y394|DHRS7_HUMAN	Dehydrogenase/reductase SDR family member 7	20.06	7
sp|P62760|VISL1_HUMAN	Visinin-like protein 1	29.32	5
sp|O15511|ARPC5_HUMAN	Actin-related protein 2/3 complex subunit 5	39.07	14
sp|P60891|PRPS1_HUMAN	Ribose-phosphate pyrophosphokinase 1	25.47	9
sp|P48739|PIPNB_HUMAN	Phosphatidylinositol transfer protein beta isoform	19.19	5
sp|P50502|F10A1_HUMAN	Hsc70-interacting protein	18.43	7
sp|Q9UNS2|CSN3_HUMAN	COP9 signalosome complex subunit 3	14.89	6
sp|P17858|PFKAL_HUMAN	ATP-dependent 6-phosphofructokinase, liver type	9.36	6
sp|Q9NZ08|ERAP1_HUMAN	Endoplasmic reticulum aminopeptidase 1	6.80	6
sp|P14868|SYDC_HUMAN	Aspartate--tRNA ligase, cytoplasmic	13.37	6
sp|Q86U17|SPA11_HUMAN	Serpin A11	14.93	6
sp|O00468|AGRIN_HUMAN	Agrin	3.58	6
sp|Q8N4T8|CBR4_HUMAN	Carbonyl reductase family member 4	28.27	8
sp|Q8NBX0|SCPDL_HUMAN	Saccharopine dehydrogenase-like oxidoreductase	19.58	5

sp|O95295|SNAPN_HUMAN	SNARE-associated protein Snapin	49.26	5
sp|Q9NUJ1|ABHDA_HUMAN	Mycophenolic acid acyl-glucuronide esterase, mitochondrial	22.88	5
sp|Q14409|GLPK3_HUMAN	Glycerol kinase 3	9.95	4
sp|P02654|APOC1_HUMAN	Apolipoprotein C-I	38.55	10
sp|Q8WTV0|SCRB1_HUMAN	Scavenger receptor class B member 1	11.59	7
sp|Q99988|GDF15_HUMAN	Growth/differentiation factor 15	23.70	5
sp|O15230|LAMA5_HUMAN	Laminin subunit alpha-5	1.76	6
sp|P51689|ARSD_HUMAN	Arylsulfatase D	15.85	8
sp|Q07065|CKAP4_HUMAN	Cytoskeleton-associated protein 4	11.13	5
sp|Q96AC1|FERM2_HUMAN	Fermitin family homolog 2	10.00	6
sp|P09012|SNRPA_HUMAN	U1 small nuclear ribonucleoprotein A	20.57	7
sp|Q00765|REEP5_HUMAN	Receptor expression-enhancing protein 5	28.57	20
sp|P67809|YBOX1_HUMAN	Nuclease-sensitive element-binding protein 1	34.26	5
sp|Q4V328|GRAP1_HUMAN	GRIP1-associated protein 1	7.73	5
sp|P25311|ZA2G_HUMAN	Zinc-alpha-2-glycoprotein	24.50	6
sp|P61201|CSN2_HUMAN	COP9 signalosome complex subunit 2	20.99	8
sp|O60749|SNX2_HUMAN	Sorting nexin-2	11.95	5
sp|Q92896|GSLG1_HUMAN	Golgi apparatus protein 1	4.75	6
sp|Q9H6S3|ES8L2_HUMAN	Epidermal growth factor receptor kinase substrate 8-like protein 2	7.41	5
sp|P48735|IDHP_HUMAN	Isocitrate dehydrogenase [NADP], mitochondrial	15.04	9
sp|Q9HAN9|NMNA1_HUMAN	Nicotinamide/nicotinic acid mononucleotide adenylyltransferase 1	15.05	4
sp|Q13126|MTAP_HUMAN	S-methyl-5'-thioadenosine phosphorylase	31.45	6
sp|Q15363|TMED2_HUMAN	Transmembrane emp24 domain-containing protein 2	30.85	9
sp|P13688|CEAM1_HUMAN	Carcinoembryonic antigen-related cell adhesion molecule 1	11.79	7
sp|O75935|DCTN3_HUMAN	Dynactin subunit 3	27.42	6
sp|Q99624|S38A3_HUMAN	Sodium-coupled neutral amino acid transporter 3	12.90	6
sp|Q04837|SSBP_HUMAN	Single-stranded DNA-binding protein, mitochondrial	47.30	8
sp|P63241|IF5A1_HUMAN	Eukaryotic translation initiation factor 5A-1	38.31	7
sp|P04004|VTNC_HUMAN	Vitronectin	19.46	8
sp|P10599|THIO_HUMAN	Thioredoxin	51.43	7
sp|P00734|THRB_HUMAN	Prothrombin	14.63	9
sp|P13646|K1C13_HUMAN	Keratin, type I cytoskeletal 13	21.18	34
sp|P22695|QCR2_HUMAN	Cytochrome b-c1 complex subunit 2, mitochondrial	14.57	4
sp|Q13439|GOGA4_HUMAN	Golgin subfamily A member 4	2.38	5
sp|Q15738|NSDHL_HUMAN	Sterol-4-alpha-carboxylate 3-dehydrogenase, decarboxylating	16.62	5
sp|P55145|MANF_HUMAN	Mesencephalic astrocyte-derived neurotrophic factor	30.77	8
sp|Q13451|FKBP5_HUMAN	Peptidyl-prolyl cis-trans isomerase FKBP5	12.04	5
sp|P63220|RS21_HUMAN	40S ribosomal protein S21	53.01	5
sp|O43826|G6PT1_HUMAN	Glucose-6-phosphate exchanger SLC37A4	13.52	9
sp|P31946|1433B_HUMAN	14-3-3 protein beta/alpha	43.90	24
sp|P05109|S10A8_HUMAN	Protein S100-A8	39.78	5
sp|Q9ULV4|COR1C_HUMAN	Coronin-1C	8.23	6
sp|P10253|LYAG_HUMAN	Lysosomal alpha-glucosidase	5.15	4
sp|Q9BX68|HINT2_HUMAN	Histidine triad nucleotide-binding protein 2, mitochondrial	44.17	5
sp|Q9Y281|COF2_HUMAN	Cofilin-2	56.02	8
sp|O75955|FLOT1_HUMAN	Flotillin-1	14.05	5
sp|Q5T2W1|NHRF3_HUMAN	Na(+)/H(+) exchange regulatory cofactor NHE-RF3	12.52	4
sp|P35542|SAA4_HUMAN	Serum amyloid A-4 protein	51.54	16
sp|Q15018|F175B_HUMAN	BRISC complex subunit Abro1	12.05	4
sp|P0CG04|IGLC1_HUMAN	Immunoglobulin lambda constant 1	53.77	9
sp|P27701|CD82_HUMAN	CD82 antigen	14.98	8
sp|P04003|C4BPA_HUMAN	C4b-binding protein alpha chain	16.75	14
sp|Q9Y2Q3|GSTK1_HUMAN	Glutathione S-transferase kappa 1	28.76	5
sp|P49638|TTPA_HUMAN	Alpha-tocopherol transfer protein	23.74	4
sp|Q9BQE5|APOL2_HUMAN	Apolipoprotein L2	17.21	5
sp|P00387|NB5R3_HUMAN	NADH-cytochrome b5 reductase 3	15.61	4

sp|P17612|KAPCA_HUMAN	cAMP-dependent protein kinase catalytic subunit alpha	15.38	4
sp|P52566|GDIR2_HUMAN	Rho GDP-dissociation inhibitor 2	31.84	4
sp|O95674|CDS2_HUMAN	Phosphatidate cytidylyltransferase 2	13.03	4
sp|P0C0L5|CO4B_HUMAN	Complement C4-B	49.54	173
sp|P07437|TBB5_HUMAN	Tubulin beta chain	49.77	22
sp|Q5D862|FILA2_HUMAN	Filaggrin-2	3.85	4
sp|Q96GD0|PLPP_HUMAN	Pyridoxal phosphate phosphatase	20.95	4
sp|P62316|SMD2_HUMAN	Small nuclear ribonucleoprotein Sm D2	32.20	5
sp|P56537|IF6_HUMAN	Eukaryotic translation initiation factor 6	27.35	4
sp|P51571|SSRD_HUMAN	Translocon-associated protein subunit delta	30.64	5
sp|P61803|DAD1_HUMAN	Dolichyl-diphosphooligosaccharide--protein glycosyltransferase subunit DAD1	35.40	5
sp|P25398|RS12_HUMAN	40S ribosomal protein S12	41.67	4
sp|P41091|IF2G_HUMAN	Eukaryotic translation initiation factor 2 subunit 3	13.98	4
sp|Q9BVG4|PBDC1_HUMAN	Protein PBDC1	19.74	4
sp|Q7L5Y1|ENOF1_HUMAN	Mitochondrial enolase superfamily member 1	15.12	5
sp|O75340|PDCD6_HUMAN	Programmed cell death protein 6	35.08	6
sp|O75356|ENTP5_HUMAN	Ectonucleoside triphosphate diphosphohydrolase 5	17.29	6
sp|Q15155|NOMO1_HUMAN	Nodal modulator 1	4.58	5
sp|Q9NUP9|LIN7C_HUMAN	Protein lin-7 homolog C	23.86	5
sp|P10155|RO60_HUMAN	60 kDa SS-A/Ro ribonucleoprotein	8.92	4
sp|P05141|ADT2_HUMAN	ADP/ATP translocase 2	16.78	5
sp|P01920|DQB1_HUMAN	HLA class II histocompatibility antigen, DQ beta 1 chain	22.61	6
sp|P12955|PEPD_HUMAN	Xaa-Pro dipeptidase	8.72	5
sp|Q92673|SORL_HUMAN	Sortilin-related receptor	2.21	4
sp|Q04760|LGUL_HUMAN	Lactoylglutathione lyase	21.74	4
sp|P05413|FABPH_HUMAN	Fatty acid-binding protein, heart	57.14	8
sp|Q9NR19|ACSA_HUMAN	Acetyl-coenzyme A synthetase, cytoplasmic	7.28	4
sp|O94874|UFL1_HUMAN	E3 UFM1-protein ligase 1	8.44	6
sp|Q8WW52|F151A_HUMAN	Protein FAM151A	8.21	4
sp|P11117|PPAL_HUMAN	Lysosomal acid phosphatase	9.22	4
sp|P50135|HNMT_HUMAN	Histamine N-methyltransferase	21.92	5
sp|Q13045|FLII_HUMAN	Protein flightless-1 homolog	3.07	5
sp|P00403|COX2_HUMAN	Cytochrome c oxidase subunit 2	24.67	6
sp|P10632|CP2C8_HUMAN	Cytochrome P450 2C8	19.80	9
sp|P00533|EGFR_HUMAN	Epidermal growth factor receptor	6.36	6
sp|O14495|PLPP3_HUMAN	Phospholipid phosphatase 3	15.11	4
sp|P30050|RL12_HUMAN	60S ribosomal protein L12	43.03	11
sp|Q13753|LAMC2_HUMAN	Laminin subunit gamma-2	3.44	4
sp|P02746|C1QB_HUMAN	Complement C1q subcomponent subunit B	19.76	4
sp|P63244|RACK1_HUMAN	Receptor of activated protein C kinase 1	19.24	6
sp|Q9NR28|DBLOH_HUMAN	Diablo homolog, mitochondrial	18.41	5
sp|Q13492|PICAL_HUMAN	Phosphatidylinositol-binding clathrin assembly protein	10.12	5
sp|Q7Z5L0|VMO1_HUMAN	Vitelline membrane outer layer protein 1 homolog	29.70	5
sp|Q09028|RBBP4_HUMAN	Histone-binding protein RBBP4	16.24	5
sp|P0DOX2|IGA2_HUMAN	Immunoglobulin alpha-2 heavy chain	26.37	27
sp|Q9NTJ4|MA2C1_HUMAN	Alpha-mannosidase 2C1	6.25	5
sp|Q15365|PCBP1_HUMAN	Poly(rC)-binding protein 1	14.89	4
sp|Q8TCT9|HM13_HUMAN	Minor histocompatibility antigen H13	14.59	4
sp|Q9BX66|SRBS1_HUMAN	Sorbin and SH3 domain-containing protein 1	5.03	5
sp|Q8IW45|NNRD_HUMAN	ATP-dependent (S)-NAD(P)H-hydrate dehydratase	17.87	4
sp|P35222|CTNB1_HUMAN	Catenin beta-1	7.43	7
sp|Q13630|FCL_HUMAN	GDP-L-fucose synthase	14.95	5
sp|P24539|AT5F1_HUMAN	ATP synthase F(0) complex subunit B1, mitochondrial	26.95	7
sp|O14745|NHRF1_HUMAN	Na(+)/H(+) exchange regulatory cofactor NHE-RF1	15.36	4
sp|P48163|MAOX_HUMAN	NADP-dependent malic enzyme	8.04	5

sp|Q8IWE2|NXP20_HUMAN	Protein NOXP20	11.55	4
sp|Q9H2M3|BHMT2_HUMAN	S-methylmethionine--homocysteine S-methyltransferase BHMT2	33.88	20
sp|Q9Y5P6|GMPPB_HUMAN	Mannose-1-phosphate guanyltransferase beta	11.94	4
sp|Q9NQG5|RPR1B_HUMAN	Regulation of nuclear pre-mRNA domain-containing protein 1B	19.33	5
sp|P22792|CPN2_HUMAN	Carboxypeptidase N subunit 2	7.71	6
sp|P01591|IGJ_HUMAN	Immunoglobulin J chain	35.22	12
sp|P62834|RAP1A_HUMAN	Ras-related protein Rap-1A	26.09	4
sp|Q6UW02|CP20A_HUMAN	Cytochrome P450 20A1	22.08	11
sp|Q9NP79|VTA1_HUMAN	Vacuolar protein sorting-associated protein VTA1 homolog	20.52	5
sp|Q15323|K1H1_HUMAN	Keratin, type I cuticular Ha1	15.38	9
sp|O14936|CSKP_HUMAN	Peripheral plasma membrane protein CASK	7.13	7
sp|P46783|RS10_HUMAN	40S ribosomal protein S10	23.64	4
sp|P04080|CYTB_HUMAN	Cystatin-B	55.10	5
sp|P16284|PECA1_HUMAN	Platelet endothelial cell adhesion molecule	6.77	4
sp|Q16706|MA2A1_HUMAN	Alpha-mannosidase 2	4.72	5
sp|P01042|KNG1_HUMAN	Kininogen-1	5.59	5
sp|P31689|DNJA1_HUMAN	DnaJ homolog subfamily A member 1	18.14	4
sp|Q8WWI5|CTL1_HUMAN	Choline transporter-like protein 1	9.28	6
sp|Q99829|CPNE1_HUMAN	Copine-1	8.01	4
sp|O94875|SRBS2_HUMAN	Sorbin and SH3 domain-containing protein 2	5.73	4
sp|Q71UI9|H2AV_HUMAN	Histone H2A.V	42.19	23
sp|Q9Y3B3|TMED7_HUMAN	Transmembrane emp24 domain-containing protein 7	23.66	5
sp|Q9NPJ3|ACO13_HUMAN	Acyl-coenzyme A thioesterase 13	43.57	5
sp|Q02413|DSG1_HUMAN	Desmoglein-1	7.15	7
sp|P62879|GBB2_HUMAN	Guanine nucleotide-binding protein G(I)/G(S)/G(T) subunit beta-2	15.29	5
sp|Q13907|IDI1_HUMAN	Isopentenyl-diphosphate Delta-isomerase 1	30.84	6
sp|O94956|SO2B1_HUMAN	Solute carrier organic anion transporter family member 2B1	7.47	4
sp|P53680|AP2S1_HUMAN	AP-2 complex subunit sigma	27.46	4
sp|P18462|1A25_HUMAN	HLA class I histocompatibility antigen, A-25 alpha chain	40.27	22
sp|Q92928|RAB1C_HUMAN	Putative Ras-related protein Rab-1C	24.88	4
sp|O14618|CCS_HUMAN	Copper chaperone for superoxide dismutase	21.17	4
sp|Q15181|IPYR_HUMAN	Inorganic pyrophosphatase	23.88	5
sp|P27144|KAD4_HUMAN	Adenylate kinase 4, mitochondrial	20.63	4
sp|P00441|SODC_HUMAN	Superoxide dismutase [Cu-Zn]	66.88	10
sp|Q92692|NECT2_HUMAN	Nectin-2	7.99	4
sp|P98160|PGBM_HUMAN	Basement membrane-specific heparan sulfate proteoglycan core protein	0.96	4
sp|Q14008|CKAP5_HUMAN	Cytoskeleton-associated protein 5	1.72	3
sp|O00151|PDLI1_HUMAN	PDZ and LIM domain protein 1	15.50	4
sp|P01040|CYTA_HUMAN	Cystatin-A	52.04	4
sp|P26599|PTBP1_HUMAN	Polypyrimidine tract-binding protein 1	20.34	7
sp|Q5JTV8|TOIP1_HUMAN	Torsin-1A-interacting protein 1	9.43	5
sp|Q16222|UAP1_HUMAN	UDP-N-acetylhexosamine pyrophosphorylase	11.69	6
sp|P39019|RS19_HUMAN	40S ribosomal protein S19	35.86	8
sp|Q92947|GCDH_HUMAN	Glutaryl-CoA dehydrogenase, mitochondrial	13.93	4
sp|Q92973|TNPO1_HUMAN	Transportin-1	4.68	4
sp|Q07960|RHG01_HUMAN	Rho GTPase-activating protein 1	10.71	3
sp|Q9BR76|COR1B_HUMAN	Coronin-1B	14.31	5
sp|P21291|CSRP1_HUMAN	Cysteine and glycine-rich protein 1	33.16	6
sp|Q9NR45|SIAS_HUMAN	Sialic acid synthase	22.01	5
sp|Q9BV19|CA050_HUMAN	Uncharacterized protein C1orf50	22.61	4
sp|Q9BTZ2|DHRS4_HUMAN	Dehydrogenase/reductase SDR family member 4	14.75	4
sp|Q9UFN0|NPS3A_HUMAN	Protein NipSnap homolog 3A	27.94	5
sp|O60313|OPA1_HUMAN	Dynamin-like 120 kDa protein, mitochondrial	5.21	4
sp|O15258|RER1_HUMAN	Protein RER1	29.59	5
sp|Q8IUE6|H2A2B_HUMAN	Histone H2A type 2-B	60.77	94

sp|O60884|DNJA2_HUMAN	DnaJ homolog subfamily A member 2	17.72	5
sp|Q13765|NACA_HUMAN	Nascent polypeptide-associated complex subunit alpha	25.58	4
sp|P61966|AP1S1_HUMAN	AP-1 complex subunit sigma-1A	30.38	5
sp|Q9H8H3|MET7A_HUMAN	Methyltransferase-like protein 7A	22.13	7
sp|Q14789|GOGB1_HUMAN	Golgin subfamily B member 1	2.82	8
sp|P13693|TCTP_HUMAN	Translationally-controlled tumor protein	34.30	5
sp|P42126|ECI1_HUMAN	Enoyl-CoA delta isomerase 1, mitochondrial	15.23	5
sp|Q13155|AIMP2_HUMAN	Aminoacyl tRNA synthase complex-interacting multifunctional protein 2	20.62	4
sp|Q15811|ITSN1_HUMAN	Intersectin-1	2.96	3
sp|P13073|COX41_HUMAN	Cytochrome c oxidase subunit 4 isoform 1, mitochondrial	24.26	4
sp|Q9UBI6|GBG12_HUMAN	Guanine nucleotide-binding protein G(I)/G(S)/G(O) subunit gamma-12	38.89	5
sp|Q07020|RL18_HUMAN	60S ribosomal protein L18	23.94	6
sp|P05156|CFAI_HUMAN	Complement factor I	5.83	4
sp|P51858|HDGF_HUMAN	Hepatoma-derived growth factor	12.92	4
sp|Q14444|CAPR1_HUMAN	Caprin-1	7.47	5
sp|Q9BUL8|PDC10_HUMAN	Programmed cell death protein 10	19.34	4
sp|Q99611|SPS2_HUMAN	Selenide, water dikinase 2	11.16	4
sp|P49913|CAMP_HUMAN	Cathelicidin antimicrobial peptide	28.82	7
sp|P10606|COX5B_HUMAN	Cytochrome c oxidase subunit 5B, mitochondrial	37.98	4
sp|P14735|IDE_HUMAN	Insulin-degrading enzyme	5.59	5
sp|Q9UJ70|NAGK_HUMAN	N-acetyl-D-glucosamine kinase	22.38	5
sp|Q96GK7|FAH2A_HUMAN	Fumarylacetoacetate hydrolase domain-containing protein 2A	16.88	4
sp|Q15029|U5S1_HUMAN	116 kDa U5 small nuclear ribonucleoprotein component	6.38	6
sp|Q9Y224|CN166_HUMAN	UPF0568 protein C14orf166	22.54	4
sp|Q00688|FKBP3_HUMAN	Peptidyl-prolyl cis-trans isomerase FKBP3	19.64	3
sp|P18085|ARF4_HUMAN	ADP-ribosylation factor 4	43.33	7
sp|P80217|IN35_HUMAN	Interferon-induced 35 kDa protein	12.94	3
sp|Q8N5K1|CISD2_HUMAN	CDGSH iron-sulfur domain-containing protein 2	25.93	3
sp|Q92522|H1X_HUMAN	Histone H1x	17.84	4
sp|P21964|COMT_HUMAN	Catechol O-methyltransferase	21.77	5
sp|P99999|CYC_HUMAN	Cytochrome c	21.90	3
sp|O75477|ERLN1_HUMAN	Erlin-1	14.45	7
sp|Q9UBQ5|EIF3K_HUMAN	Eukaryotic translation initiation factor 3 subunit K	34.86	7
sp|Q15165|PON2_HUMAN	Serum paraoxonase/arylesterase 2	19.77	5
sp|Q9H488|OFUT1_HUMAN	GDP-fucose protein O-fucosyltransferase 1	13.14	3
sp|P02760|AMBP_HUMAN	Protein AMBP	17.33	6
sp|P51452|DUS3_HUMAN	Dual specificity protein phosphatase 3	19.46	3
sp|P60033|CD81_HUMAN	CD81 antigen	28.81	14
sp|O15305|PMM2_HUMAN	Phosphomannomutase 2	20.33	5
sp|O14672|ADA10_HUMAN	Disintegrin and metalloproteinase domain-containing protein 10	4.41	3
sp|P20962|PTMS_HUMAN	Parathymosin	22.55	7
sp|P23246|SFPQ_HUMAN	Splicing factor, proline- and glutamine-rich	7.07	4
sp|Q5ZPR3|CD276_HUMAN	CD276 antigen	15.36	5
sp|P02765|FETUA_HUMAN	Alpha-2-HS-glycoprotein	17.44	5
sp|Q8IV08|PLD3_HUMAN	Phospholipase D3	8.37	4
sp|Q14258|TRI25_HUMAN	E3 ubiquitin/ISG15 ligase TRIM25	5.87	4
sp|P07711|CATL1_HUMAN	Cathepsin L1	17.72	8
sp|P62826|RAN_HUMAN	GTP-binding nuclear protein Ran	18.52	4
sp|P81605|DCD_HUMAN	Dermcidin	30.91	7
sp|P78537|BL1S1_HUMAN	Biogenesis of lysosome-related organelles complex 1 subunit 1	19.61	3
sp|Q13177|PAK2_HUMAN	Serine/threonine-protein kinase PAK 2	11.64	5
sp|P52272|HNRPM_HUMAN	Heterogeneous nuclear ribonucleoprotein M	7.81	5
sp|P15374|UCHL3_HUMAN	Ubiquitin carboxyl-terminal hydrolase isozyme L3	28.70	5
sp|O75347|TBCA_HUMAN	Tubulin-specific chaperone A	35.19	4

sp|P02042|HBD_HUMAN	Hemoglobin subunit delta	92.52	82
sp|P07108|ACBP_HUMAN	Acyl-CoA-binding protein	52.87	5
sp|A1L0T0|ILVBL_HUMAN	Acetolactate synthase-like protein	10.44	4
sp|O43493|TGON2_HUMAN	Trans-Golgi network integral membrane protein 2	9.79	3
sp|P60866|RS20_HUMAN	40S ribosomal protein S20	22.69	3
sp|Q13232|NDK3_HUMAN	Nucleoside diphosphate kinase 3	23.67	3
sp|O75531|BAF_HUMAN	Barrier-to-autointegration factor	42.70	3
sp|O75223|GGCT_HUMAN	Gamma-glutamylcyclotransferase	17.55	3
sp|P13761|2B17_HUMAN	HLA class II histocompatibility antigen, DRB1-7 beta chain	30.45	24
sp|Q99720|SGMR1_HUMAN	Sigma non-opioid intracellular receptor 1	18.83	6
sp|Q8WW59|SPRY4_HUMAN	SPRY domain-containing protein 4	14.98	3
sp|P67812|SC11A_HUMAN	Signal peptidase complex catalytic subunit SEC11A	15.64	3
sp|P04440|DPB1_HUMAN	HLA class II histocompatibility antigen, DP beta 1 chain	13.18	5
sp|P56385|ATP5I_HUMAN	ATP synthase subunit e, mitochondrial	60.87	4
sp|Q9UKM9|RALY_HUMAN	RNA-binding protein Raly	8.50	3
sp|P68366|TBA4A_HUMAN	Tubulin alpha-4A chain	30.36	18
sp|P22392|NDKB_HUMAN	Nucleoside diphosphate kinase B	48.68	10
sp|P79483|DRB3_HUMAN	HLA class II histocompatibility antigen, DR beta 3 chain	30.45	21
sp|Q02338|BDH_HUMAN	D-beta-hydroxybutyrate dehydrogenase, mitochondrial	20.41	5
sp|P13762|DRB4_HUMAN	HLA class II histocompatibility antigen, DR beta 4 chain	28.95	7
sp|P12259|FA5_HUMAN	Coagulation factor V	1.71	3
sp|O43169|CYB5B_HUMAN	Cytochrome b5 type B	44.52	4
sp|A0A0C4DH73|KV112_HUMAN	Immunoglobulin kappa variable 1-12	13.68	3
sp|Q9UBV2|SE1L1_HUMAN	Protein sel-1 homolog 1	5.92	3
sp|P52597|HNRPF_HUMAN	Heterogeneous nuclear ribonucleoprotein F	12.05	3
sp|P01619|KV320_HUMAN	Immunoglobulin kappa variable 3-20	37.07	3
sp|Q9Y5L4|TIM13_HUMAN	Mitochondrial import inner membrane translocase subunit Tim13	53.68	7
sp|Q9BVJ7|DUS23_HUMAN	Dual specificity protein phosphatase 23	16.00	3
sp|Q07812|BAX_HUMAN	Apoptosis regulator BAX	18.75	5
sp|Q96AB3|ISOC2_HUMAN	Isochorismatase domain-containing protein 2	34.63	3
sp|Q15125|EBP_HUMAN	3-beta-hydroxysteroid-Delta(8),Delta(7)-isomerase	16.52	7
sp|P82909|RT36_HUMAN	28S ribosomal protein S36, mitochondrial	44.66	3
sp|P43307|SSRA_HUMAN	Translocon-associated protein subunit alpha	11.89	3
sp|P30273|FCERG_HUMAN	High affinity immunoglobulin epsilon receptor subunit gamma	32.56	3
sp|O75352|MPU1_HUMAN	Mannose-P-dolichol utilization defect 1 protein	13.77	3
sp|P51991|ROA3_HUMAN	Heterogeneous nuclear ribonucleoprotein A3	10.85	3
sp|Q92905|CSN5_HUMAN	COP9 signalosome complex subunit 5	19.46	4
sp|Q9Y3I0|RTCB_HUMAN	tRNA-splicing ligase RtcB homolog	7.13	4
sp|Q93034|CUL5_HUMAN	Cullin-5	5.26	4
sp|P48637|GSHB_HUMAN	Glutathione synthetase	9.92	4
sp|Q9NZK5|CECR1_HUMAN	Adenosine deaminase CECR1	10.76	4
sp|Q9H1E3|NUCKS_HUMAN	Nuclear ubiquitous casein and cyclin-dependent kinase substrate 1	25.51	5
sp|Q99471|PFD5_HUMAN	Prefoldin subunit 5	24.68	3
sp|Q96FW1|OTUB1_HUMAN	Ubiquitin thioesterase OTUB1	15.50	3
sp|P43034|LIS1_HUMAN	Platelet-activating factor acetylhydrolase IB subunit alpha	12.93	5
sp|Q15435|PP1R7_HUMAN	Protein phosphatase 1 regulatory subunit 7	13.33	4
sp|O95255|MRP6_HUMAN	Multidrug resistance-associated protein 6	2.93	4
sp|Q14353|GAMT_HUMAN	Guanidinoacetate N-methyltransferase	18.64	3
sp|Q9H479|FN3K_HUMAN	Fructosamine-3-kinase	12.62	3
sp|O00339|MATN2_HUMAN	Matrilin-2	3.35	3
sp|Q8N1G4|LRC47_HUMAN	Leucine-rich repeat-containing protein 47	10.63	4
sp|Q15041|AR6P1_HUMAN	ADP-ribosylation factor-like protein 6-interacting protein 1	18.23	9
sp|Q9H9B4|SFXN1_HUMAN	Sideroflexin-1	15.22	4
sp|P01859|IGHG2_HUMAN	Immunoglobulin heavy constant gamma 2	38.65	22
sp|P20702|ITAX_HUMAN	Integrin alpha-X	5.16	7
sp|Q9UHB9|SRP68_HUMAN	Signal recognition particle subunit SRP68	5.90	4

sp|Q99808|S29A1_HUMAN	Equilibrative nucleoside transporter 1	8.55	3
sp|P30793|GCH1_HUMAN	GTP cyclohydrolase 1	14.40	4
sp|O14744|ANM5_HUMAN	Protein arginine N-methyltransferase 5	6.12	4
sp|Q9BXD5|NPL_HUMAN	N-acetylneuraminate lyase	20.94	5
sp|Q9Y6N5|SQRD_HUMAN	Sulfide:quinone oxidoreductase, mitochondrial	8.44	3
sp|P50416|CPT1A_HUMAN	Carnitine O-palmitoyltransferase 1, liver isoform	4.66	3
sp|P09382|LEG1_HUMAN	Galectin-1	23.70	3
sp|Q9UKR5|ERG28_HUMAN	Probable ergosterol biosynthetic protein 28	22.86	3
sp|P55058|PLTP_HUMAN	Phospholipid transfer protein	20.28	7
sp|Q92945|FUBP2_HUMAN	Far upstream element-binding protein 2	6.33	3
sp|Q6P587|FAHD1_HUMAN	Acylpyruvase FAHD1, mitochondrial	27.23	3
sp|Q9NQ48|LZTL1_HUMAN	Leucine zipper transcription factor-like protein 1	15.72	4
sp|O60841|IF2P_HUMAN	Eukaryotic translation initiation factor 5B	4.34	3
sp|Q08AH3|ACS2A_HUMAN	Acyl-coenzyme A synthetase ACSM2A, mitochondrial	34.32	17
sp|P62330|ARF6_HUMAN	ADP-ribosylation factor 6	20.57	5
sp|P61163|ACTZ_HUMAN	Alpha-centractin	31.12	7
sp|P63162|RSMN_HUMAN	Small nuclear ribonucleoprotein-associated protein N	15.83	4
sp|P53007|TXTP_HUMAN	Tricarboxylate transport protein, mitochondrial	10.29	3
sp|O75937|DNJC8_HUMAN	DnaJ homolog subfamily C member 8	13.04	3
sp|Q9H993|ARMT1_HUMAN	Protein-glutamate O-methyltransferase	11.56	3
sp|P12004|PCNA_HUMAN	Proliferating cell nuclear antigen	14.94	3
sp|P24821|TENA_HUMAN	Tenascin	1.95	4
sp|Q9NYL9|TMOD3_HUMAN	Tropomodulin-3	15.63	4
sp|Q16629|SRSF7_HUMAN	Serine/arginine-rich splicing factor 7	21.01	8
sp|P30508|1C12_HUMAN	HLA class I histocompatibility antigen, Cw-12 alpha chain	33.33	18
sp|P78310|CXAR_HUMAN	Coxsackievirus and adenovirus receptor	8.49	3
sp|Q6UW68|TM205_HUMAN	Transmembrane protein 205	26.98	8
sp|P15121|ALDR_HUMAN	Aldose reductase	14.56	6
sp|P62244|RS15A_HUMAN	40S ribosomal protein S15a	31.54	4
sp|Q9P0K7|RAI14_HUMAN	Ankycorbin	6.02	6
sp|O95197|RTN3_HUMAN	Reticulon-3	3.00	8
sp|P50851|LRBA_HUMAN	Lipopolysaccharide-responsive and beige-like anchor protein	1.54	4
sp|Q99598|TSNAX_HUMAN	Translin-associated protein X	18.62	4
sp|O96005|CLPT1_HUMAN	Cleft lip and palate transmembrane protein 1	8.52	4
sp|P36578|RL4_HUMAN	60S ribosomal protein L4	11.24	4
sp|P55769|NH2L1_HUMAN	NHP2-like protein 1	30.47	5
sp|P50579|MAP2_HUMAN	Methionine aminopeptidase 2	10.25	4
sp|Q92598|HS105_HUMAN	Heat shock protein 105 kDa	6.06	4
sp|P80188|NGAL_HUMAN	Neutrophil gelatinase-associated lipocalin	38.89	7
sp|Q9BY49|PECR_HUMAN	Peroxisomal trans-2-enoyl-CoA reductase	18.15	4
sp|Q14CZ8|HECAM_HUMAN	Hepatocyte cell adhesion molecule	10.34	3
sp|P62913|RL11_HUMAN	60S ribosomal protein L11	19.66	4
sp|Q86WU2|LDHD_HUMAN	Probable D-lactate dehydrogenase, mitochondrial	5.92	3
sp|Q01105|SET_HUMAN	Protein SET	11.72	3
sp|Q02083|NAAA_HUMAN	N-acylethanolamine-hydrolyzing acid amidase	12.81	4
sp|O95881|TXD12_HUMAN	Thioredoxin domain-containing protein 12	25.58	3
sp|P20645|MPRD_HUMAN	Cation-dependent mannose-6-phosphate receptor	19.13	3
sp|O14579|COPE_HUMAN	Coatomer subunit epsilon	15.91	4
sp|O76094|SRP72_HUMAN	Signal recognition particle subunit SRP72	4.62	3
sp|P08240|SRPRA_HUMAN	Signal recognition particle receptor subunit alpha	7.68	5
sp|O00469|PLOD2_HUMAN	Procollagen-lysine,2-oxoglutarate 5-dioxygenase 2	8.41	5
sp|Q8WZA9|IRGQ_HUMAN	Immunity-related GTPase family Q protein	10.75	4
sp|O14980|XPO1_HUMAN	Exportin-1	7.56	6
sp|Q99538|LGMN_HUMAN	Legumain	17.55	5
sp|P22309|UD11_HUMAN	UDP-glucuronosyltransferase 1-1	28.71	23
sp|Q08722|CD47_HUMAN	Leukocyte surface antigen CD47	8.67	3

sp|P31949|S10AB_HUMAN	Protein S100-A11	42.86	4
sp|Q12996|CSTF3_HUMAN	Cleavage stimulation factor subunit 3	5.30	3
sp|Q7L1Q6|BZW1_HUMAN	Basic leucine zipper and W2 domain-containing protein 1	13.37	6
sp|Q14232|EI2BA_HUMAN	Translation initiation factor eIF-2B subunit alpha	13.11	3
sp|Q8IZP0|ABI1_HUMAN	Abl interactor 1	7.09	3
sp|P62854|RS26_HUMAN	40S ribosomal protein S26	33.91	3
sp|P14618|KPYM_HUMAN	Pyruvate kinase PKM	19.21	8
sp|Q8N163|CCAR2_HUMAN	Cell cycle and apoptosis regulator protein 2	3.03	3
sp|P49326|FMO5_HUMAN	Dimethylaniline monooxygenase [N-oxide-forming] 5	6.19	3
sp|P23381|SYWC_HUMAN	Tryptophan--tRNA ligase, cytoplasmic	8.70	3
sp|Q8TEB1|DCA11_HUMAN	DDB1- and CUL4-associated factor 11	6.59	3
sp|Q9BZZ2|SN_HUMAN	Sialoadhesin	3.51	4
sp|P05090|APOD_HUMAN	Apolipoprotein D	21.16	4
sp|Q9UHJ6|SHPK_HUMAN	Sedoheptulokinase	9.41	3
sp|A8MWL6|SNG2L_HUMAN	Putative synaptogyrin-2 like protein	8.07	2
sp|P61769|B2MG_HUMAN	Beta-2-microglobulin	30.25	5
sp|O15031|PLXB2_HUMAN	Plexin-B2	2.28	3
sp|Q9BSH5|HDHD3_HUMAN	Haloacid dehalogenase-like hydrolase domain-containing protein 3	17.93	3
sp|Q9H008|LHPP_HUMAN	Phospholysine phosphohistidine inorganic pyrophosphate phosphatase	17.78	4
sp|Q9C0E8|LNP_HUMAN	Protein lunapark	8.65	3
sp|P62140|PP1B_HUMAN	Serine/threonine-protein phosphatase PP1-beta catalytic subunit	14.98	5
sp|Q9NYL4|FKB11_HUMAN	Peptidyl-prolyl cis-trans isomerase FKBP11	14.43	4
sp|P61619|S61A1_HUMAN	Protein transport protein Sec61 subunit alpha isoform 1	6.51	4
sp|Q03426|KIME_HUMAN	Mevalonate kinase	8.33	3
sp|Q00839|HNRPU_HUMAN	Heterogeneous nuclear ribonucleoprotein U	5.33	3
sp|P39687|AN32A_HUMAN	Acidic leucine-rich nuclear phosphoprotein 32 family member A	12.45	5
sp|Q9NSE4|SYIM_HUMAN	Isoleucine--tRNA ligase, mitochondrial	3.85	3
sp|Q14651|PLSI_HUMAN	Plastin-1	15.42	10
sp|Q16555|DPYL2_HUMAN	Dihydropyrimidinase-related protein 2	7.87	5
sp|O14773|TPP1_HUMAN	Tripeptidyl-peptidase 1	12.43	5
sp|O95445|APOM_HUMAN	Apolipoprotein M	38.30	7
sp|P08962|CD63_HUMAN	CD63 antigen	8.40	7
sp|Q9Y2V2|CHSP1_HUMAN	Calcium-regulated heat-stable protein 1	34.69	3
sp|Q8IWA5|CTL2_HUMAN	Choline transporter-like protein 2	5.67	4
sp|P62888|RL30_HUMAN	60S ribosomal protein L30	33.91	4
sp|Q9Y5M8|SRPRB_HUMAN	Signal recognition particle receptor subunit beta	15.87	3
sp|P02753|RET4_HUMAN	Retinol-binding protein 4	28.86	11
sp|Q9Y3D6|FIS1_HUMAN	Mitochondrial fission 1 protein	16.45	5
sp|P35237|SPB6_HUMAN	Serpin B6	11.17	4
sp|P01780|HV307_HUMAN	Immunoglobulin heavy variable 3-7	41.03	9
sp|Q14533|KRT81_HUMAN	Keratin, type II cuticular Hb1	8.52	7
sp|O95671|ASML_HUMAN	N-acetylserotonin O-methyltransferase-like protein	4.51	3
sp|P09668|CATH_HUMAN	Pro-cathepsin H	14.63	4
sp|P04217|A1BG_HUMAN	Alpha-1B-glycoprotein	11.31	6
sp|Q14019|COTL1_HUMAN	Coactosin-like protein	31.69	6
sp|Q9Y639|NPTN_HUMAN	Neuroplastin	9.04	3
sp|P36551|HEM6_HUMAN	Oxygen-dependent coproporphyrinogen-III oxidase, mitochondrial	9.25	4
sp|O15067|PUR4_HUMAN	Phosphoribosylformylglycinamidine synthase	3.06	3
sp|Q05086|UBE3A_HUMAN	Ubiquitin-protein ligase E3A	3.89	3
sp|Q13283|G3BP1_HUMAN	Ras GTPase-activating protein-binding protein 1	8.58	3
sp|Q9UBR2|CATZ_HUMAN	Cathepsin Z	9.57	3
sp|P13489|RINI_HUMAN	Ribonuclease inhibitor	7.37	3
sp|P57105|SYJ2B_HUMAN	Synaptojanin-2-binding protein	31.72	4
sp|Q96EK6|GNA1_HUMAN	Glucosamine 6-phosphate N-acetyltransferase	29.89	4
sp|P49961|ENTP1_HUMAN	Ectonucleoside triphosphate diphosphohydrolase 1	7.25	3

sp|Q8NHL6|LIRB1_HUMAN	Leukocyte immunoglobulin-like receptor subfamily B member 1	7.54	4
sp|Q96MV1|TMM56_HUMAN	Transmembrane protein 56	12.55	3
sp|Q04826|1B40_HUMAN	HLA class I histocompatibility antigen, B-40 alpha chain	41.16	20
sp|P15880|RS2_HUMAN	40S ribosomal protein S2	8.53	3
sp|P08729|K2C7_HUMAN	Keratin, type II cytoskeletal 7	25.59	42
sp|Q13347|EIF3I_HUMAN	Eukaryotic translation initiation factor 3 subunit I	18.77	5
sp|Q53EL6|PDCD4_HUMAN	Programmed cell death protein 4	6.82	3
sp|Q04695|K1C17_HUMAN	Keratin, type I cytoskeletal 17	26.39	26
sp|Q96F10|SAT2_HUMAN	Diamine acetyltransferase 2	21.76	4
sp|Q99613|EIF3C_HUMAN	Eukaryotic translation initiation factor 3 subunit C	3.29	3
sp|P42166|LAP2A_HUMAN	Lamina-associated polypeptide 2, isoform alpha	5.91	3
sp|A6NLP5|TTC36_HUMAN	Tetratricopeptide repeat protein 36	24.34	3
sp|P62750|RL23A_HUMAN	60S ribosomal protein L23a	19.87	3
sp|Q9H6R3|ACSS3_HUMAN	Acyl-CoA synthetase short-chain family member 3, mitochondrial	5.69	3
sp|P11441|UBL4A_HUMAN	Ubiquitin-like protein 4A	18.47	3
sp|P30533|AMRP_HUMAN	Alpha-2-macroglobulin receptor-associated protein	6.72	2
sp|Q8IWW8|HOT_HUMAN	Hydroxyacid-oxoacid transhydrogenase, mitochondrial	9.42	3
sp|Q13409|DC1I2_HUMAN	Cytoplasmic dynein 1 intermediate chain 2	5.33	3
sp|O15143|ARC1B_HUMAN	Actin-related protein 2/3 complex subunit 1B	12.63	6
sp|O75970|MPDZ_HUMAN	Multiple PDZ domain protein	1.74	3
sp|P26373|RL13_HUMAN	60S ribosomal protein L13	13.27	3
sp|O95571|ETHE1_HUMAN	Persulfide dioxygenase ETHE1, mitochondrial	16.14	3
sp|P31944|CASPE_HUMAN	Caspase-14	13.22	3
sp|P16455|MGMT_HUMAN	Methylated-DNA--protein-cysteine methyltransferase	19.32	3
sp|Q8NI22|MCFD2_HUMAN	Multiple coagulation factor deficiency protein 2	63.70	5
sp|P52790|HXK3_HUMAN	Hexokinase-3	4.98	4
sp|P53370|NUDT6_HUMAN	Nucleoside diphosphate-linked moiety X motif 6	13.61	3
sp|P23588|IF4B_HUMAN	Eukaryotic translation initiation factor 4B	7.53	3
sp|P02749|APOH_HUMAN	Beta-2-glycoprotein 1	17.10	7
sp|P06454|PTMA_HUMAN	Prothymosin alpha	34.23	3
sp|Q9C0C2|TB182_HUMAN	182 kDa tankyrase-1-binding protein	1.39	2
sp|L0R6Q1|S35U4_HUMAN	SLC35A4 upstream open reading frame protein	38.83	3
sp|P60468|SC61B_HUMAN	Protein transport protein Sec61 subunit beta	37.50	4
sp|P31513|FMO3_HUMAN	Dimethylaniline monooxygenase [N-oxide-forming] 3	6.39	3
sp|P20674|COX5A_HUMAN	Cytochrome c oxidase subunit 5A, mitochondrial	25.33	3
sp|P20160|CAP7_HUMAN	Azurocidin	17.53	7
sp|Q15661|TRYB1_HUMAN	Tryptase alpha/beta-1	13.09	3
sp|P06748|NPM_HUMAN	Nucleophosmin	10.20	2
sp|Q9Y5Z4|HEBP2_HUMAN	Heme-binding protein 2	12.20	2
sp|O76003|GLRX3_HUMAN	Glutaredoxin-3	11.04	2
sp|P55196|AFAD_HUMAN	Afadin	1.37	2
sp|Q15233|NONO_HUMAN	Non-POU domain-containing octamer-binding protein	9.77	6
sp|P04279|SEMG1_HUMAN	Semenogelin-1	9.31	4
sp|Q15758|AAAT_HUMAN	Neutral amino acid transporter B(0)	5.91	2
sp|P34949|MPI_HUMAN	Mannose-6-phosphate isomerase	15.60	4
sp|P00915|CAH1_HUMAN	Carbonic anhydrase 1	11.49	3
sp|O14786|NRP1_HUMAN	Neuropilin-1	5.09	3
sp|P62314|SMD1_HUMAN	Small nuclear ribonucleoprotein Sm D1	37.82	4
sp|Q96A72|MGN2_HUMAN	Protein mago nashi homolog 2	17.57	3
sp|Q08188|TGM3_HUMAN	Protein-glutamine gamma-glutamyltransferase E	3.18	2
sp|P31146|COR1A_HUMAN	Coronin-1A	5.64	2
sp|O75368|SH3L1_HUMAN	SH3 domain-binding glutamic acid-rich-like protein	19.30	2
sp|P61019|RAB2A_HUMAN	Ras-related protein Rab-2A	20.28	4
sp|O43324|MCA3_HUMAN	Eukaryotic translation elongation factor 1 epsilon-1	12.64	2
sp|P06865|HEXA_HUMAN	Beta-hexosaminidase subunit alpha	11.34	6
sp|P20340|RAB6A_HUMAN	Ras-related protein Rab-6A	16.35	3

sp|Q14520|HABP2_HUMAN	Hyaluronan-binding protein 2	9.11	4
sp|P54840|GYS2_HUMAN	Glycogen [starch] synthase, liver	3.13	2
sp|O75608|LYPA1_HUMAN	Acyl-protein thioesterase 1	15.65	3
sp|Q9Y5K5|UCHL5_HUMAN	Ubiquitin carboxyl-terminal hydrolase isozyme L5	9.73	3
sp|P62241|RS8_HUMAN	40S ribosomal protein S8	10.58	2
sp|P04259|K2C6B_HUMAN	Keratin, type II cytoskeletal 6B	50.35	87
sp|Q9UBV8|PEF1_HUMAN	Peflin	15.85	4
sp|Q9Y6B6|SAR1B_HUMAN	GTP-binding protein SAR1b	17.17	3
sp|Q7Z5G4|GOGA7_HUMAN	Golgin subfamily A member 7	18.98	2
sp|P11233|RALA_HUMAN	Ras-related protein Ral-A	16.02	3
sp|P42330|AK1C3_HUMAN	Aldo-keto reductase family 1 member C3	34.67	18
sp|P46782|RS5_HUMAN	40S ribosomal protein S5	28.92	7
sp|O95456|PSMG1_HUMAN	Proteasome assembly chaperone 1	13.19	3
sp|P80748|LV321_HUMAN	Immunoglobulin lambda variable 3-21	38.46	6
sp|P51580|TPMT_HUMAN	Thiopurine S-methyltransferase	8.98	2
sp|O75348|VATG1_HUMAN	V-type proton ATPase subunit G 1	22.03	2
sp|O00461|GOLI4_HUMAN	Golgi integral membrane protein 4	5.46	3
sp|Q9UL25|RAB21_HUMAN	Ras-related protein Rab-21	18.67	3
sp|O14979|HNRDL_HUMAN	Heterogeneous nuclear ribonucleoprotein D-like	10.00	4
sp|P61011|SRP54_HUMAN	Signal recognition particle 54 kDa protein	7.34	3
sp|Q9P2T1|GMPR2_HUMAN	GMP reductase 2	13.51	3
sp|Q9BPX5|ARP5L_HUMAN	Actin-related protein 2/3 complex subunit 5-like protein	33.99	6
sp|Q02878|RL6_HUMAN	60S ribosomal protein L6	9.72	3
sp|P05154|IPSP_HUMAN	Plasma serine protease inhibitor	10.10	4
sp|P68402|PA1B2_HUMAN	Platelet-activating factor acetylhydrolase IB subunit beta	12.23	2
sp|A0A0B4J1V0|HV315_HUMAN	Immunoglobulin heavy variable 3-15	37.82	4
sp|Q07955|SRSF1_HUMAN	Serine/arginine-rich splicing factor 1	7.66	2
sp|P25774|CATS_HUMAN	Cathepsin S	6.34	2
sp|O95777|LSM8_HUMAN	U6 snRNA-associated Sm-like protein LSm8	19.79	2
sp|P28676|GRAN_HUMAN	Grancalcin	21.66	4
sp|Q9NUQ6|SPS2L_HUMAN	SPATS2-like protein	5.38	2
sp|P60953|CDC42_HUMAN	Cell division control protein 42 homolog	19.90	4
sp|P24666|PPAC_HUMAN	Low molecular weight phosphotyrosine protein phosphatase	12.66	2
sp|Q9P000|COMD9_HUMAN	COMM domain-containing protein 9	14.14	2
sp|O43583|DENR_HUMAN	Density-regulated protein	14.14	3
sp|Q92887|MRP2_HUMAN	Canalicular multispecific organic anion transporter 1	2.07	3
sp|P31930|QCR1_HUMAN	Cytochrome b-c1 complex subunit 1, mitochondrial	6.25	2
sp|P68036|UB2L3_HUMAN	Ubiquitin-conjugating enzyme E2 L3	15.58	2
sp|Q14032|BAAT_HUMAN	Bile acid-CoA:amino acid N-acyltransferase	6.70	2
sp|Q9NNW7|TRXR2_HUMAN	Thioredoxin reductase 2, mitochondrial	8.97	3
sp|P50148|GNAQ_HUMAN	Guanine nucleotide-binding protein G(q) subunit alpha	6.68	2
sp|P36969|GPX4_HUMAN	Phospholipid hydroperoxide glutathione peroxidase, mitochondrial	13.20	2
sp|P62304|RUXE_HUMAN	Small nuclear ribonucleoprotein E	25.00	2
sp|Q92900|RENT1_HUMAN	Regulator of nonsense transcripts 1	2.48	2
sp|P50895|BCAM_HUMAN	Basal cell adhesion molecule	3.98	2
sp|O60220|TIM8A_HUMAN	Mitochondrial import inner membrane translocase subunit Tim8 A	52.58	3
sp|Q96GA7|SDSL_HUMAN	Serine dehydratase-like	10.64	3
sp|P35637|FUS_HUMAN	RNA-binding protein FUS	7.22	2
sp|P16949|STMN1_HUMAN	Stathmin	16.78	2
sp|Q9UM00|TMCO1_HUMAN	Calcium load-activated calcium channel	12.77	2
sp|Q9Y333|LSM2_HUMAN	U6 snRNA-associated Sm-like protein LSm2	31.58	2
sp|Q9BSJ8|ESYT1_HUMAN	Extended synaptotagmin-1	2.17	2
sp|Q92575|UBXN4_HUMAN	UBX domain-containing protein 4	6.89	2
sp|P62753|RS6_HUMAN	40S ribosomal protein S6	8.43	3
sp|P45880|VDAC2_HUMAN	Voltage-dependent anion-selective channel protein 2	7.48	2
sp|Q68D91|MBLC2_HUMAN	Metallo-beta-lactamase domain-containing protein 2	10.04	2

sp|P78324|SHPS1_HUMAN	Tyrosine-protein phosphatase non-receptor type substrate 1	4.96	2
sp|Q07021|C1QBP_HUMAN	Complement component 1 Q subcomponent-binding protein, mitochondrial	12.06	2
sp|P06312|KV401_HUMAN	Immunoglobulin kappa variable 4-1	24.79	3
sp|Q9UI32|GLSL_HUMAN	Glutaminase liver isoform, mitochondrial	4.15	2
sp|Q9BUP3|HTAI2_HUMAN	Oxidoreductase HTATIP2	9.50	2
sp|P84090|ERH_HUMAN	Enhancer of rudimentary homolog	21.15	2
sp|P63218|GBG5_HUMAN	Guanine nucleotide-binding protein G(I)/G(S)/G(O) subunit gamma-5	27.94	3
sp|P42677|RS27_HUMAN	40S ribosomal protein S27	28.57	2
sp|O75964|ATP5L_HUMAN	ATP synthase subunit g, mitochondrial	27.18	2
sp|O00584|RNT2_HUMAN	Ribonuclease T2	10.16	2
sp|Q9NRQ2|PLS4_HUMAN	Phospholipid scramblase 4	6.69	2
sp|P52895|AK1C2_HUMAN	Aldo-keto reductase family 1 member C2	31.27	18
sp|P0DOY2|IGLC2_HUMAN	Immunoglobulin lambda constant 2	63.21	16
sp|Q30167|2B1A_HUMAN	HLA class II histocompatibility antigen, DRB1-10 beta chain	31.95	17
sp|Q15366|PCBP2_HUMAN	Poly(rC)-binding protein 2	12.05	3
sp|A0A075B6K4|LV310_HUMAN	Immunoglobulin lambda variable 3-10	23.48	2
sp|Q99627|CSN8_HUMAN	COP9 signalosome complex subunit 8	22.97	3
sp|Q63HM1|KFA_HUMAN	Kynurenine formamidase	7.26	2
sp|P62318|SMD3_HUMAN	Small nuclear ribonucleoprotein Sm D3	15.08	4
sp|P49006|MRP_HUMAN	MARCKS-related protein	14.36	3
sp|P30049|ATPD_HUMAN	ATP synthase subunit delta, mitochondrial	13.69	2
sp|P61626|LYSC_HUMAN	Lysozyme C	12.84	3
sp|Q07666|KHDR1_HUMAN	KH domain-containing, RNA-binding, signal transduction-associated protein 1	5.42	2
sp|P01033|TIMP1_HUMAN	Metalloproteinase inhibitor 1	10.14	2
sp|O00754|MA2B1_HUMAN	Lysosomal alpha-mannosidase	2.37	2
sp|Q9H5X1|FA96A_HUMAN	MIP18 family protein FAM96A	12.50	2
sp|P35268|RL22_HUMAN	60S ribosomal protein L22	18.75	2
sp|P14209|CD99_HUMAN	CD99 antigen	18.92	3
sp|P04433|KV311_HUMAN	Immunoglobulin kappa variable 3-11	23.48	2
sp|Q96IZ0|PAWR_HUMAN	PRKC apoptosis WT1 regulator protein	8.53	2
sp|Q969L2|MAL2_HUMAN	Protein MAL2	18.18	2
sp|Q8NCR9|CLRN3_HUMAN	Clarin-3	12.39	2
sp|Q10589|BST2_HUMAN	Bone marrow stromal antigen 2	13.89	2
sp|Q08623|HDHD1_HUMAN	Pseudouridine-5'-phosphatase	11.40	2
sp|P62857|RS28_HUMAN	40S ribosomal protein S28	30.43	3
sp|P60059|SC61G_HUMAN	Protein transport protein Sec61 subunit gamma	36.76	2
sp|P14406|CX7A2_HUMAN	Cytochrome c oxidase subunit 7A2, mitochondrial	27.71	2
sp|P14174|MIF_HUMAN	Macrophage migration inhibitory factor	17.39	8
sp|P01624|KV315_HUMAN	Immunoglobulin kappa variable 3-15	23.48	2
sp|O95832|CLD1_HUMAN	Claudin-1	14.69	2
sp|O60613|SEP15_HUMAN	Selenoprotein F	15.43	4
sp|O95822|DCMC_HUMAN	Malonyl-CoA decarboxylase, mitochondrial	7.50	3
sp|Q9Y3B4|SF3B6_HUMAN	Splicing factor 3B subunit 6	20.80	2
sp|Q6ZMI0|PPR21_HUMAN	Protein phosphatase 1 regulatory subunit 21	5.13	3
sp|P53602|MVD1_HUMAN	Diphosphomevalonate decarboxylase	5.75	2
sp|Q13586|STIM1_HUMAN	Stromal interaction molecule 1	3.65	3
sp|Q16186|ADRM1_HUMAN	Proteasomal ubiquitin receptor ADRM1	7.62	2
sp|P55957|BID_HUMAN	BH3-interacting domain death agonist	12.31	2
sp|P01116|RASK_HUMAN	GTPase KRas	17.46	3
sp|O75947|ATP5H_HUMAN	ATP synthase subunit d, mitochondrial	17.39	3
sp|O75636|FCN3_HUMAN	Ficolin-3	8.03	5
sp|O95487|SC24B_HUMAN	Protein transport protein Sec24B	4.73	4
sp|Q9Y5B9|SP16H_HUMAN	FACT complex subunit SPT16	3.06	3
sp|Q6YN16|HSDL2_HUMAN	Hydroxysteroid dehydrogenase-like protein 2	8.85	3

sp|Q15370|ELOB_HUMAN	Elongin-B	24.58	3
sp|Q5Y7A7|2B1D_HUMAN	HLA class II histocompatibility antigen, DRB1-13 beta chain	30.83	9
sp|P12273|PIP_HUMAN	Prolactin-inducible protein	15.07	2
sp|Q12857|NFIA_HUMAN	Nuclear factor 1 A-type	4.91	2
sp|O43852|CALU_HUMAN	Calumenin	13.65	4
sp|Q9H0X4|F234A_HUMAN	Protein FAM234A	4.71	2
sp|P08246|ELNE_HUMAN	Neutrophil elastase	6.37	2
sp|P15291|B4GT1_HUMAN	Beta-1,4-galactosyltransferase 1	2.01	1
sp|P12318|FCG2A_HUMAN	Low affinity immunoglobulin gamma Fc region receptor II-a	8.52	3
sp|P0CG29|GST2_HUMAN	Glutathione S-transferase theta-2	14.34	3
sp|P08697|A2AP_HUMAN	Alpha-2-antiplasmin	6.31	2
sp|Q9Y4Z0|LSM4_HUMAN	U6 snRNA-associated Sm-like protein LSm4	20.14	4
sp|P40763|STAT3_HUMAN	Signal transducer and activator of transcription 3	2.86	2
sp|P14384|CBPM_HUMAN	Carboxypeptidase M	16.70	6
sp|P12830|CADH1_HUMAN	Cadherin-1	2.61	2
sp|Q7Z794|K2C1B_HUMAN	Keratin, type II cytoskeletal 1b	19.03	38
sp|Q8NBJ7|SUMF2_HUMAN	Sulfatase-modifying factor 2	12.62	4
sp|Q9Y3F4|STRAP_HUMAN	Serine-threonine kinase receptor-associated protein	8.00	2
sp|Q86VB7|C163A_HUMAN	Scavenger receptor cysteine-rich type 1 protein M130	9.34	11
sp|Q709F0|ACD11_HUMAN	Acyl-CoA dehydrogenase family member 11	6.92	5
sp|P09497|CLCB_HUMAN	Clathrin light chain B	8.30	2
sp|P33778|H2B1B_HUMAN	Histone H2B type 1-B	57.14	127
sp|O75915|PRAF3_HUMAN	PRA1 family protein 3	24.47	6
sp|O43815|STRN_HUMAN	Striatin	1.03	1
sp|Q93008|USP9X_HUMAN	Probable ubiquitin carboxyl-terminal hydrolase FAF-X	0.74	2
sp|P22059|OSBP1_HUMAN	Oxysterol-binding protein 1	5.08	3
sp|P61457|PHS_HUMAN	Pterin-4-alpha-carbinolamine dehydratase	20.19	3
sp|Q6P1A2|MBOA5_HUMAN	Lysophospholipid acyltransferase 5	6.37	3
sp|P62266|RS23_HUMAN	40S ribosomal protein S23	16.08	2
sp|Q9HCN8|SDF2L_HUMAN	Stromal cell-derived factor 2-like protein 1	20.81	4
sp|P61970|NTF2_HUMAN	Nuclear transport factor 2	18.90	2
sp|P24534|EF1B_HUMAN	Elongation factor 1-beta	24.44	6
sp|P01909|DQA1_HUMAN	HLA class II histocompatibility antigen, DQ alpha 1 chain	8.27	2
sp|O75751|S22A3_HUMAN	Solute carrier family 22 member 3	4.14	2
sp|Q93009|UBP7_HUMAN	Ubiquitin carboxyl-terminal hydrolase 7	3.36	4
sp|Q9Y5X1|SNX9_HUMAN	Sorting nexin-9	3.36	2
sp|O95563|MPC2_HUMAN	Mitochondrial pyruvate carrier 2	25.20	3
sp|Q6P4A8|PLBL1_HUMAN	Phospholipase B-like 1	10.49	4
sp|Q5TFE4|NT5D1_HUMAN	5'-nucleotidase domain-containing protein 1	6.15	2
sp|O14735|CDIPT_HUMAN	CDP-diacylglycerol--inositol 3-phosphatidyltransferase	9.86	2
sp|Q9UBI1|COMD3_HUMAN	COMM domain-containing protein 3	10.77	2
sp|Q04637|IF4G1_HUMAN	Eukaryotic translation initiation factor 4 gamma 1	1.38	2
sp|Q5MY95|ENTP8_HUMAN	Ectonucleoside triphosphate diphosphohydrolase 8	6.46	3
sp|P0DOX4|IGE_HUMAN	Immunoglobulin epsilon heavy chain	4.75	2
sp|Q969J3|BORC5_HUMAN	BLOC-1-related complex subunit 5	14.80	3
sp|P13473|LAMP2_HUMAN	Lysosome-associated membrane glycoprotein 2	4.88	2
sp|Q969I6|S38A4_HUMAN	Sodium-coupled neutral amino acid transporter 4	3.47	2
sp|Q8N5M1|ATPF2_HUMAN	ATP synthase mitochondrial F1 complex assembly factor 2	7.27	2
sp|Q9Y5S9|RBM8A_HUMAN	RNA-binding protein 8A	11.49	2
sp|O60869|EDF1_HUMAN	Endothelial differentiation-related factor 1	15.54	2
sp|O75489|NDUS3_HUMAN	NADH dehydrogenase [ubiquinone] iron-sulfur protein 3, mitochondrial	9.85	2
sp|Q5JRX3|PREP_HUMAN	Presequence protease, mitochondrial	2.51	2
sp|P49888|ST1E1_HUMAN	Estrogen sulfotransferase	6.80	2
sp|P15151|PVR_HUMAN	Poliovirus receptor	11.27	3
sp|P21397|AOFA_HUMAN	Amine oxidase [flavin-containing] A	13.47	8

sp|P07203|GPX1_HUMAN	Glutathione peroxidase 1	20.20	3
sp|Q99542|MMP19_HUMAN	Matrix metalloproteinase-19	6.69	2
sp|Q8N1N4|K2C78_HUMAN	Keratin, type II cytoskeletal 78	13.27	14
sp|Q96IX5|USMG5_HUMAN	Up-regulated during skeletal muscle growth protein 5	43.10	2
sp|Q14558|KPRA_HUMAN	Phosphoribosyl pyrophosphate synthase-associated protein 1	16.29	4
sp|P82970|HMGN5_HUMAN	High mobility group nucleosome-binding domain-containing protein 5	22.34	3
sp|P20701|ITAL_HUMAN	Integrin alpha-L	2.39	2
sp|P37108|SRP14_HUMAN	Signal recognition particle 14 kDa protein	26.47	3
sp|P63027|VAMP2_HUMAN	Vesicle-associated membrane protein 2	14.66	1
sp|Q99735|MGST2_HUMAN	Microsomal glutathione S-transferase 2	10.88	2
sp|Q8NC51|PAIRB_HUMAN	Plasminogen activator inhibitor 1 RNA-binding protein	11.03	5
sp|Q08209|PP2BA_HUMAN	Serine/threonine-protein phosphatase 2B catalytic subunit alpha isoform	7.49	3
sp|Q15833|STXB2_HUMAN	Syntaxin-binding protein 2	5.73	2
sp|O60888|CUTA_HUMAN	Protein CutA	15.64	2
sp|Q9UMX5|NENF_HUMAN	Neudesin	11.63	2
sp|Q8WUA8|TSK_HUMAN	Tsukushin	7.08	2
sp|P17735|ATTY_HUMAN	Tyrosine aminotransferase	6.17	3
sp|Q9NPF4|OSGEP_HUMAN	Probable tRNA N6-adenosine threonylcarbamoyltransferase	6.87	2
sp|P62847|RS24_HUMAN	40S ribosomal protein S24	19.55	2
sp|Q9H3Z4|DNJC5_HUMAN	DnaJ homolog subfamily C member 5	8.59	2
sp|Q13596|SNX1_HUMAN	Sorting nexin-1	6.32	3
sp|Q6P3W7|SCYL2_HUMAN	SCY1-like protein 2	1.94	2
sp|P27216|ANX13_HUMAN	Annexin A13	11.39	3
sp|Q15102|PA1B3_HUMAN	Platelet-activating factor acetylhydrolase IB subunit gamma	7.79	2
sp|Q86VS8|HOOK3_HUMAN	Protein Hook homolog 3	3.06	2
sp|P11182|ODB2_HUMAN	Lipoamide acyltransferase component of branched-chain alpha-keto acid dehydrogenase complex, mitochondrial	7.26	2
sp|Q6Y1H2|HACD2_HUMAN	Very-long-chain (3R)-3-hydroxyacyl-CoA dehydratase 2	8.66	2
sp|P18827|SDC1_HUMAN	Syndecan-1	9.35	2
sp|O43813|LANC1_HUMAN	LanC-like protein 1	6.27	2
sp|Q16658|FSCN1_HUMAN	Fascin	5.07	2
sp|P35244|RFA3_HUMAN	Replication protein A 14 kDa subunit	22.31	2
sp|Q12931|TRAP1_HUMAN	Heat shock protein 75 kDa, mitochondrial	7.67	6
sp|O43684|BUB3_HUMAN	Mitotic checkpoint protein BUB3	10.06	3
sp|Q86XE5|HOGA1_HUMAN	4-hydroxy-2-oxoglutarate aldolase, mitochondrial	8.87	2
sp|Q96PE7|MCEE_HUMAN	Methylmalonyl-CoA epimerase, mitochondrial	12.50	2
sp|A0A0G2JMI3|HV692_HUMAN	Immunoglobulin heavy variable 1-69-2	10.26	4
sp|Q5VWZ2|LYPL1_HUMAN	Lysophospholipase-like protein 1	10.97	2
sp|P28907|CD38_HUMAN	ADP-ribosyl cyclase/cyclic ADP-ribose hydrolase 1	8.33	3
sp|P01034|CYTC_HUMAN	Cystatin-C	19.18	2
sp|O60216|RAD21_HUMAN	Double-strand-break repair protein rad21 homolog	3.96	2
sp|Q00325|MPCP_HUMAN	Phosphate carrier protein, mitochondrial	9.12	3
sp|P14927|QCR7_HUMAN	Cytochrome b-c1 complex subunit 7	25.23	2
sp|P54725|RD23A_HUMAN	UV excision repair protein RAD23 homolog A	12.40	5
sp|Q9Y365|PCTL_HUMAN	PCTP-like protein	8.25	2
sp|Q96FZ7|CHMP6_HUMAN	Charged multivesicular body protein 6	9.95	2
sp|P05204|HMGN2_HUMAN	Non-histone chromosomal protein HMG-17	34.44	2
sp|Q9BVC6|TM109_HUMAN	Transmembrane protein 109	13.17	5
sp|Q9Y223|GLCNE_HUMAN	Bifunctional UDP-N-acetylglucosamine 2-epimerase/N-acetylmannosamine kinase	3.32	2
sp|P49411|EFTU_HUMAN	Elongation factor Tu, mitochondrial	11.95	4
sp|O75795|UDB17_HUMAN	UDP-glucuronosyltransferase 2B17	36.79	25
sp|P61009|SPCS3_HUMAN	Signal peptidase complex subunit 3	16.67	6
sp|O75521|ECI2_HUMAN	Enoyl-CoA delta isomerase 2, mitochondrial	6.60	2

sp|P33908|MA1A1_HUMAN	Mannosyl-oligosaccharide 1,2-alpha-mannosidase IA	4.13	2
sp|Q13185|CBX3_HUMAN	Chromobox protein homolog 3	16.39	2
sp|P09496|CLCA_HUMAN	Clathrin light chain A	10.89	6
sp|Q9BRA2|TXD17_HUMAN	Thioredoxin domain-containing protein 17	15.45	2
sp|P15927|RFA2_HUMAN	Replication protein A 32 kDa subunit	7.41	2
sp|Q9UKV8|AGO2_HUMAN	Protein argonaute-2	7.45	5
sp|Q9GZQ3|COMD5_HUMAN	COMM domain-containing protein 5	8.48	2
sp|P68032|ACTC_HUMAN	Actin, alpha cardiac muscle 1	48.28	54
sp|P57088|TMM33_HUMAN	Transmembrane protein 33	8.50	2
sp|P63092|GNAS2_HUMAN	Guanine nucleotide-binding protein G(s) subunit alpha isoforms short	11.42	4
sp|P47985|UCRI_HUMAN	Cytochrome b-c1 complex subunit Rieske, mitochondrial	7.66	2
sp|Q15819|UB2V2_HUMAN	Ubiquitin-conjugating enzyme E2 variant 2	13.10	2
sp|Q687X5|STEA4_HUMAN	Metalloreductase STEAP4	7.19	2
sp|O75764|TCEA3_HUMAN	Transcription elongation factor A protein 3	4.88	2
sp|Q13867|BLMH_HUMAN	Bleomycin hydrolase	6.81	2
sp|P59768|GBG2_HUMAN	Guanine nucleotide-binding protein G(I)/G(S)/G(O) subunit gamma-2	39.44	2
sp|P04066|FUCO_HUMAN	Tissue alpha-L-fucosidase	4.72	2
sp|P60842|IF4A1_HUMAN	Eukaryotic initiation factor 4A-I	21.43	10
sp|O95834|EMAL2_HUMAN	Echinoderm microtubule-associated protein-like 2	3.70	2
sp|P61353|RL27_HUMAN	60S ribosomal protein L27	12.50	2
sp|Q9H3N1|TMX1_HUMAN	Thioredoxin-related transmembrane protein 1	7.86	2
sp|Q9NP72|RAB18_HUMAN	Ras-related protein Rab-18	9.22	2
sp|A6NCS6|CB072_HUMAN	Uncharacterized protein C2orf72	12.54	2
sp|Q3SY84|K2C71_HUMAN	Keratin, type II cytoskeletal 71	10.71	15
sp|P62841|RS15_HUMAN	40S ribosomal protein S15	22.07	3
sp|P35030|TRY3_HUMAN	Trypsin-3	22.70	81
sp|Q9NSB2|KRT84_HUMAN	Keratin, type II cuticular Hb4	10.50	14
sp|P62917|RL8_HUMAN	60S ribosomal protein L8	7.00	2
sp|Q04609|FOLH1_HUMAN	Glutamate carboxypeptidase 2	6.80	3
sp|Q02539|H11_HUMAN	Histone H1.1	16.74	12
sp|Q08554|DSC1_HUMAN	Desmocollin-1	3.02	2
sp|Q9UK55|ZPI_HUMAN	Protein Z-dependent protease inhibitor	5.86	2
sp|P02730|B3AT_HUMAN	Band 3 anion transport protein	2.42	2
sp|Q9NSD9|SYFB_HUMAN	Phenylalanine--tRNA ligase beta subunit	3.23	2
sp|Q9UEY8|ADDG_HUMAN	Gamma-adducin	4.11	3
sp|Q6IA69|NADE_HUMAN	Glutamine-dependent NAD(+) synthetase	4.67	3
sp|P35503|UD13_HUMAN	UDP-glucuronosyltransferase 1-3	31.84	26
sp|O60879|DIAP2_HUMAN	Protein diaphanous homolog 2	3.91	4
sp|P28330|ACADL_HUMAN	Long-chain specific acyl-CoA dehydrogenase, mitochondrial	5.81	2
sp|Q15404|RSU1_HUMAN	Ras suppressor protein 1	9.75	2
sp|Q96M27|PRRC1_HUMAN	Protein PRRC1	6.29	2
sp|P61313|RL15_HUMAN	60S ribosomal protein L15	14.71	3
sp|P23368|MAOM_HUMAN	NAD-dependent malic enzyme, mitochondrial	1.54	1
sp|O76013|KRT36_HUMAN	Keratin, type I cuticular Ha6	6.64	7
sp|Q9NPD3|EXOS4_HUMAN	Exosome complex component RRP41	13.88	3
sp|Q15427|SF3B4_HUMAN	Splicing factor 3B subunit 4	7.55	2
sp|Q9Y6G5|COMDA_HUMAN	COMM domain-containing protein 10	10.40	2
sp|Q567U6|CCD93_HUMAN	Coiled-coil domain-containing protein 93	3.96	2
sp|P20930|FILA_HUMAN	Filaggrin	0.59	2
sp|P20036|DPA1_HUMAN	HLA class II histocompatibility antigen, DP alpha 1 chain	18.46	4
sp|P18124|RL7_HUMAN	60S ribosomal protein L7	13.31	4
sp|Q14141|SEPT6_HUMAN	Septin-6	15.21	9
sp|P25685|DNJB1_HUMAN	DnaJ homolog subfamily B member 1	7.35	2
sp|Q9UQB8|BAIP2_HUMAN	Brain-specific angiogenesis inhibitor 1-associated protein 2	4.17	2

sp|O75821|EIF3G_HUMAN	Eukaryotic translation initiation factor 3 subunit G	10.63	2
sp|P48960|CD97_HUMAN	CD97 antigen	3.95	2
sp|Q96T51|RUFY1_HUMAN	RUN and FYVE domain-containing protein 1	3.11	2
sp|Q03591|FHR1_HUMAN	Complement factor H-related protein 1	18.79	8
sp|O75390|CISY_HUMAN	Citrate synthase, mitochondrial	2.36	1
sp|Q12792|TWF1_HUMAN	Twinfilin-1	8.00	3
sp|P01861|IGHG4_HUMAN	Immunoglobulin heavy constant gamma 4	58.41	26
sp|P98066|TSG6_HUMAN	Tumor necrosis factor-inducible gene 6 protein	8.66	2
sp|P62306|RUXF_HUMAN	Small nuclear ribonucleoprotein F	39.53	2
sp|P08574|CY1_HUMAN	Cytochrome c1, heme protein, mitochondrial	8.61	2
sp|Q13057|COASY_HUMAN	Bifunctional coenzyme A synthase	6.92	2
sp|O75844|FACE1_HUMAN	CAAX prenyl protease 1 homolog	4.63	2
sp|P62312|LSM6_HUMAN	U6 snRNA-associated Sm-like protein LSm6	13.75	1
sp|Q86YT5|S13A5_HUMAN	Solute carrier family 13 member 5	1.23	1
sp|P61586|RHOA_HUMAN	Transforming protein RhoA	24.87	5
sp|P15735|PHKG2_HUMAN	Phosphorylase b kinase gamma catalytic chain, liver/testis isoform	5.91	2
sp|Q86V81|THOC4_HUMAN	THO complex subunit 4	15.95	4
sp|P26022|PTX3_HUMAN	Pentraxin-related protein PTX3	7.09	2
sp|P42285|SK2L2_HUMAN	Superkiller viralicidic activity 2-like 2	2.40	2
sp|P13987|CD59_HUMAN	CD59 glycoprotein	15.63	2
sp|P15907|SIAT1_HUMAN	Beta-galactoside alpha-2,6-sialyltransferase 1	6.40	2
sp|Q9H4M9|EHD1_HUMAN	EH domain-containing protein 1	6.18	3
sp|Q92882|OSTF1_HUMAN	Osteoclast-stimulating factor 1	5.61	1
sp|Q9H788|SH24A_HUMAN	SH2 domain-containing protein 4A	2.42	1
sp|P32942|ICAM3_HUMAN	Intercellular adhesion molecule 3	1.83	1
sp|Q9UDR5|AASS_HUMAN	Alpha-aminoadipic semialdehyde synthase, mitochondrial	3.56	3
sp|P53634|CATC_HUMAN	Dipeptidyl peptidase 1	5.40	2
sp|P36021|MOT8_HUMAN	Monocarboxylate transporter 8	6.49	3
sp|Q6UX53|MET7B_HUMAN	Methyltransferase-like protein 7B	9.43	2
sp|Q5VYK3|ECM29_HUMAN	Proteasome-associated protein ECM29 homolog	1.36	2
sp|Q9NSB4|KRT82_HUMAN	Keratin, type II cuticular Hb2	7.21	4
sp|Q6Q788|APOA5_HUMAN	Apolipoprotein A-V	10.93	4
sp|O75436|VP26A_HUMAN	Vacuolar protein sorting-associated protein 26A	7.64	2
sp|P02452|CO1A1_HUMAN	Collagen alpha-1(I) chain	6.56	13
sp|P08579|RU2B_HUMAN	U2 small nuclear ribonucleoprotein B"	12.00	3
sp|Q13445|TMED1_HUMAN	Transmembrane emp24 domain-containing protein 1	17.62	3
sp|P62899|RL31_HUMAN	60S ribosomal protein L31	13.60	2
sp|P05386|RLA1_HUMAN	60S acidic ribosomal protein P1	66.67	11
sp|Q9Y608|LRRF2_HUMAN	Leucine-rich repeat flightless-interacting protein 2	3.61	2
sp|O75695|XRP2_HUMAN	Protein XRP2	5.14	2
sp|Q96QR8|PURB_HUMAN	Transcriptional activator protein Pur-beta	7.69	2
sp|Q01415|GALK2_HUMAN	N-acetylgalactosamine kinase	3.27	1
sp|Q86WA6|BPHL_HUMAN	Valacyclovir hydrolase	7.22	2
sp|Q5SSJ5|HP1B3_HUMAN	Heterochromatin protein 1-binding protein 3	3.44	2
sp|Q14204|DYHC1_HUMAN	Cytoplasmic dynein 1 heavy chain 1	0.60	2
sp|Q0VF96|CGNL1_HUMAN	Cingulin-like protein 1	1.46	2
sp|Q15369|ELOC_HUMAN	Elongin-C	18.75	2
sp|Q9Y5K6|CD2AP_HUMAN	CD2-associated protein	3.44	2
sp|Q9H0E2|TOLIP_HUMAN	Toll-interacting protein	3.29	1
sp|Q8NCW5|NNRE_HUMAN	NAD(P)H-hydrate epimerase	6.25	1
sp|Q9Y4X1|UD2A1_HUMAN	UDP-glucuronosyltransferase 2A1	16.32	9
sp|P42224|STAT1_HUMAN	Signal transducer and activator of transcription 1-alpha/beta	3.33	2
sp|O75146|HIP1R_HUMAN	Huntingtin-interacting protein 1-related protein	0.94	1
sp|P06681|CO2_HUMAN	Complement C2	2.79	2
sp|P15170|ERF3A_HUMAN	Eukaryotic peptide chain release factor GTP-binding subunit ERF3A	4.01	2

sp|P62310|LSM3_HUMAN	U6 snRNA-associated Sm-like protein LSm3	32.35	2
sp|P17900|SAP3_HUMAN	Ganglioside GM2 activator	5.18	1
sp|Q14449|GRB14_HUMAN	Growth factor receptor-bound protein 14	1.67	1
sp|P48740|MASP1_HUMAN	Mannan-binding lectin serine protease 1	3.72	6
sp|P61081|UBC12_HUMAN	NEDD8-conjugating enzyme Ubc12	5.46	1
sp|P31943|HNRH1_HUMAN	Heterogeneous nuclear ribonucleoprotein H	7.35	2
sp|Q49AH0|CDNF_HUMAN	Cerebral dopamine neurotrophic factor	5.35	1
sp|A9UHW6|MI4GD_HUMAN	MIF4G domain-containing protein	5.86	1
sp|Q8N766|EMC1_HUMAN	ER membrane protein complex subunit 1	1.11	1
sp|O43615|TIM44_HUMAN	Mitochondrial import inner membrane translocase subunit TIM44	2.43	1
sp|P50454|SERPH_HUMAN	Serpin H1	9.09	2
sp|P10619|PPGB_HUMAN	Lysosomal protective protein	4.58	2
sp|O75439|MPPB_HUMAN	Mitochondrial-processing peptidase subunit beta	4.29	2
sp|P61086|UBE2K_HUMAN	Ubiquitin-conjugating enzyme E2 K	6.00	1
sp|O15260|SURF4_HUMAN	Surfeit locus protein 4	13.38	3
sp|P55056|APOC4_HUMAN	Apolipoprotein C-IV	18.11	2
sp|Q9BXK5|B2L13_HUMAN	Bcl-2-like protein 13	8.25	2
sp|O00757|F16P2_HUMAN	Fructose-1,6-bisphosphatase isozyme 2	15.34	10
sp|Q9UKL6|PPCT_HUMAN	Phosphatidylcholine transfer protein	8.41	2
sp|P09543|CN37_HUMAN	2',3'-cyclic-nucleotide 3'-phosphodiesterase	2.14	1
sp|P61960|UFM1_HUMAN	Ubiquitin-fold modifier 1	17.65	1
sp|P31025|LCN1_HUMAN	Lipocalin-1	6.25	1
sp|P61964|WDR5_HUMAN	WD repeat-containing protein 5	11.08	3
sp|P35754|GLRX1_HUMAN	Glutaredoxin-1	10.38	1
sp|P55735|SEC13_HUMAN	Protein SEC13 homolog	14.60	5
sp|Q9UHL4|DPP2_HUMAN	Dipeptidyl peptidase 2	2.85	1
sp|Q16864|VATF_HUMAN	V-type proton ATPase subunit F	19.33	2
sp|Q9NZM3|ITSN2_HUMAN	Intersectin-2	1.12	1
sp|Q9HAV7|GRPE1_HUMAN	GrpE protein homolog 1, mitochondrial	4.61	1
sp|Q8NFW8|NEUA_HUMAN	N-acylneuraminate cytidylyltransferase	2.77	1
sp|Q01469|FABP5_HUMAN	Fatty acid-binding protein, epidermal	13.33	2
sp|P19652|A1AG2_HUMAN	Alpha-1-acid glycoprotein 2	24.38	5
sp|P26447|S10A4_HUMAN	Protein S100-A4	24.75	3
sp|Q15717|ELAV1_HUMAN	ELAV-like protein 1	3.37	1
sp|Q9NSK7|CS012_HUMAN	Protein C19orf12	12.50	2
sp|Q9BT09|CNPY3_HUMAN	Protein canopy homolog 3	4.32	1
sp|Q9UL12|SARDH_HUMAN	Sarcosine dehydrogenase, mitochondrial	2.40	2
sp|Q9NNX6|CD209_HUMAN	CD209 antigen	13.37	1
sp|P49458|SRP09_HUMAN	Signal recognition particle 9 kDa protein	22.09	2
sp|P06276|CHLE_HUMAN	Cholinesterase	3.49	2
sp|P43487|RANG_HUMAN	Ran-specific GTPase-activating protein	5.47	1
sp|Q86UD1|OAF_HUMAN	Out at first protein homolog	6.59	2
sp|P24158|PRTN3_HUMAN	Myeloblastin	7.81	2
sp|Q9NXG2|THUM1_HUMAN	THUMP domain-containing protein 1	3.12	1
sp|Q9NUQ3|TXLNG_HUMAN	Gamma-taxilin	4.92	3
sp|P12821|ACE_HUMAN	Angiotensin-converting enzyme	1.76	2
sp|O43617|TPPC3_HUMAN	Trafficking protein particle complex subunit 3	6.11	1
sp|P02549|SPTA1_HUMAN	Spectrin alpha chain, erythrocytic 1	0.74	2
sp|Q16718|NDUA5_HUMAN	NADH dehydrogenase [ubiquinone] 1 alpha subcomplex subunit 5	8.62	1
sp|P61020|RAB5B_HUMAN	Ras-related protein Rab-5B	21.86	4
sp|O00602|FCN1_HUMAN	Ficolin-1	9.82	3
sp|Q5TDH0|DDI2_HUMAN	Protein DDI1 homolog 2	6.02	2
sp|Q14126|DSG2_HUMAN	Desmoglein-2	1.52	1
sp|Q32P28|P3H1_HUMAN	Prolyl 3-hydroxylase 1	2.04	1
sp|P31151|S10A7_HUMAN	Protein S100-A7	31.68	4
sp|O95470|SGPL1_HUMAN	Sphingosine-1-phosphate lyase 1	1.94	1

sp|Q8N5M9|JAGN1_HUMAN	Protein jagunal homolog 1	13.11	2
sp|P12694|ODBA_HUMAN	2-oxoisovalerate dehydrogenase subunit alpha, mitochondrial	5.62	2
sp|Q9Y2R0|COA3_HUMAN	Cytochrome c oxidase assembly factor 3 homolog, mitochondrial	9.43	1
sp|O43676|NDUB3_HUMAN	NADH dehydrogenase [ubiquinone] 1 beta subcomplex subunit 3	10.20	1
sp|Q9HBI6|CP4FB_HUMAN	Phylloquinone omega-hydroxylase CYP4F11	13.17	7
sp|P07902|GALT_HUMAN	Galactose-1-phosphate uridylyltransferase	2.64	1
sp|P49914|MTHFS_HUMAN	5-formyltetrahydrofolate cyclo-ligase	10.84	3
sp|Q9NXR7|BRE_HUMAN	BRCA1-A complex subunit BRE	3.66	1
sp|Q9Y6M5|ZNT1_HUMAN	Zinc transporter 1	2.37	1
sp|Q9UHV9|PFD2_HUMAN	Prefoldin subunit 2	9.09	1
sp|Q99729|ROAA_HUMAN	Heterogeneous nuclear ribonucleoprotein A/B	5.42	2
sp|P13612|ITA4_HUMAN	Integrin alpha-4	1.84	1
sp|Q68DN1|CB016_HUMAN	Uncharacterized protein C2orf16	0.71	1
sp|P10124|SRGN_HUMAN	Serglycin	8.23	1
sp|Q9HAB8|PPCS_HUMAN	Phosphopantothenate--cysteine ligase	3.54	1
sp|P08621|RU17_HUMAN	U1 small nuclear ribonucleoprotein 70 kDa	2.52	1
sp|O00291|HIP1_HUMAN	Huntingtin-interacting protein 1	1.54	2
sp|P26196|DDX6_HUMAN	Probable ATP-dependent RNA helicase DDX6	2.28	1
sp|P07307|ASGR2_HUMAN	Asialoglycoprotein receptor 2	3.54	1
sp|Q9Y694|S22A7_HUMAN	Solute carrier family 22 member 7	1.64	1
sp|Q9BWH2|FUND2_HUMAN	FUN14 domain-containing protein 2	12.70	2
sp|P49915|GUAA_HUMAN	GMP synthase [glutamine-hydrolyzing]	1.59	1
sp|Q6P996|PDXD1_HUMAN	Pyridoxal-dependent decarboxylase domain-containing protein 1	1.40	1
sp|P10109|ADX_HUMAN	Adrenodoxin, mitochondrial	4.89	1
sp|Q7Z7B0|FLIP1_HUMAN	Filamin-A-interacting protein 1	0.74	1
sp|Q6UWP8|SBSN_HUMAN	Suprabasin	9.15	1
sp|C4AMC7|WASH3_HUMAN	Putative WAS protein family homolog 3	1.94	1
sp|Q12768|WASC5_HUMAN	WASH complex subunit 5	0.95	1
sp|Q9BRG1|VPS25_HUMAN	Vacuolar protein-sorting-associated protein 25	5.68	1
sp|P12724|ECP_HUMAN	Eosinophil cationic protein	7.50	1
sp|Q96C86|DCPS_HUMAN	m7GpppX diphosphatase	2.97	1
sp|Q99417|MYCBP_HUMAN	C-Myc-binding protein	10.68	1
sp|Q96NA2|RILP_HUMAN	Rab-interacting lysosomal protein	4.24	1
sp|O75387|LAT3_HUMAN	Large neutral amino acids transporter small subunit 3	1.61	2
sp|Q96FQ6|S10AG_HUMAN	Protein S100-A16	10.68	1
sp|Q7Z392|TPC11_HUMAN	Trafficking protein particle complex subunit 11	0.79	1
sp|P10321|1C07_HUMAN	HLA class I histocompatibility antigen, Cw-7 alpha chain	38.25	16
sp|Q9UN86|G3BP2_HUMAN	Ras GTPase-activating protein-binding protein 2	5.19	2
sp|Q92626|PXDN_HUMAN	Peroxidasin homolog	0.81	1
sp|Q6ZMZ3|SYNE3_HUMAN	Nesprin-3	0.92	1
sp|P17813|EGLN_HUMAN	Endoglin	2.74	1
sp|P36405|ARL3_HUMAN	ADP-ribosylation factor-like protein 3	6.04	1
sp|Q13442|HAP28_HUMAN	28 kDa heat- and acid-stable phosphoprotein	10.50	1
sp|O75208|COQ9_HUMAN	Ubiquinone biosynthesis protein COQ9, mitochondrial	2.83	1
sp|O00170|AIP_HUMAN	AH receptor-interacting protein	4.24	1
sp|Q9Y4G6|TLN2_HUMAN	Talin-2	3.58	11
sp|P20039|2B1B_HUMAN	HLA class II histocompatibility antigen, DRB1-11 beta chain	24.81	15
sp|Q8IYM0|F186B_HUMAN	Protein FAM186B	0.90	1
sp|Q99439|CNN2_HUMAN	Calponin-2	3.56	1
sp|Q8TF72|SHRM3_HUMAN	Protein Shroom3	0.60	4
sp|Q96IY4|CBPB2_HUMAN	Carboxypeptidase B2	5.67	2
sp|A2VDF0|FUCM_HUMAN	Fucose mutarotase	10.39	1
sp|P04908|H2A1B_HUMAN	Histone H2A type 1-B/E	60.77	100
sp|Q96KR1|ZFR_HUMAN	Zinc finger RNA-binding protein	1.02	1
sp|Q14244|MAP7_HUMAN	Ensconsin	1.20	1
sp|O60716|CTND1_HUMAN	Catenin delta-1	1.24	1

sp|Q99653|CHP1_HUMAN	Calcineurin B homologous protein 1	6.15	1
sp|P33240|CSTF2_HUMAN	Cleavage stimulation factor subunit 2	1.21	1
sp|A0A075B6S5|KV127_HUMAN	Immunoglobulin kappa variable 1-27	29.06	3
sp|P14207|FOLR2_HUMAN	Folate receptor beta	9.80	2
sp|P52294|IMA5_HUMAN	Importin subunit alpha-5	2.04	1
sp|Q9UIV1|CNOT7_HUMAN	CCR4-NOT transcription complex subunit 7	3.51	1
sp|P84085|ARF5_HUMAN	ADP-ribosylation factor 5	38.89	7
sp|O75688|PPM1B_HUMAN	Protein phosphatase 1B	8.14	3
sp|Q496Y0|LONF3_HUMAN	LON peptidase N-terminal domain and RING finger protein 3	1.58	1
sp|Q9UEW3|MARCO_HUMAN	Macrophage receptor MARCO	1.73	1
sp|Q969I3|GLYL1_HUMAN	Glycine N-acyltransferase-like protein 1	3.64	1
sp|O15347|HMGB3_HUMAN	High mobility group protein B3	6.50	1
sp|O14964|HGS_HUMAN	Hepatocyte growth factor-regulated tyrosine kinase substrate	1.54	1
sp|Q53H82|LACB2_HUMAN	Endoribonuclease LACTB2	3.13	1
sp|Q9UI10|EI2BD_HUMAN	Translation initiation factor eIF-2B subunit delta	2.68	1
sp|Q99733|NP1L4_HUMAN	Nucleosome assembly protein 1-like 4	2.93	1
sp|Q658P3|STEA3_HUMAN	Metalloreductase STEAP3	2.25	1
sp|P43007|SATT_HUMAN	Neutral amino acid transporter A	2.07	1
sp|Q9HBK9|AS3MT_HUMAN	Arsenite methyltransferase	3.20	1
sp|P13164|IFM1_HUMAN	Interferon-induced transmembrane protein 1	12.80	1
sp|P01700|LV147_HUMAN	Immunoglobulin lambda variable 1-47	35.04	3
sp|Q99424|ACOX2_HUMAN	Peroxisomal acyl-coenzyme A oxidase 2	2.79	1
sp|Q6IAA8|LTOR1_HUMAN	Ragulator complex protein LAMTOR1	8.08	1
sp|Q8IWV7|UBR1_HUMAN	E3 ubiquitin-protein ligase UBR1	0.51	1
sp|Q9UJ41|RABX5_HUMAN	Rab5 GDP/GTP exchange factor	1.13	1
sp|Q9BV57|MTND_HUMAN	1,2-dihydroxy-3-keto-5-methylthiopentene dioxygenase	7.82	1
sp|Q04323|UBXN1_HUMAN	UBX domain-containing protein 1	4.04	1
sp|P46459|NSF_HUMAN	Vesicle-fusing ATPase	1.34	1
sp|P26583|HMGB2_HUMAN	High mobility group protein B2	6.22	1
sp|Q9H8S9|MOB1A_HUMAN	MOB kinase activator 1A	5.09	1
sp|Q9UJ68|MSRA_HUMAN	Mitochondrial peptide methionine sulfoxide reductase	6.38	1
sp|Q9H8M9|EVA1A_HUMAN	Protein eva-1 homolog A	7.24	1
sp|Q9BY50|SC11C_HUMAN	Signal peptidase complex catalytic subunit SEC11C	5.21	1
sp|Q99584|S10AD_HUMAN	Protein S100-A13	11.22	1
sp|P98179|RBM3_HUMAN	RNA-binding protein 3	11.46	1
sp|P58546|MTPN_HUMAN	Myotrophin	14.41	1
sp|O95873|CF047_HUMAN	Uncharacterized protein C6orf47	8.16	1
sp|O43657|TSN6_HUMAN	Tetraspanin-6	5.31	1
sp|O00442|RTCA_HUMAN	RNA 3'-terminal phosphate cyclase	2.73	1
sp|Q9UI14|PRAF1_HUMAN	Prenylated Rab acceptor protein 1	8.11	1
sp|Q9UHA4|LTOR3_HUMAN	Ragulator complex protein LAMTOR3	8.07	1
sp|Q6IPR1|ETFR1_HUMAN	Electron transfer flavoprotein regulatory factor 1	10.00	1
sp|Q14242|SELPL_HUMAN	P-selectin glycoprotein ligand 1	2.18	1
sp|P78556|CCL20_HUMAN	C-C motif chemokine 20	7.29	1
sp|O43809|CPSF5_HUMAN	Cleavage and polyadenylation specificity factor subunit 5	7.93	1
sp|P08263|GSTA1_HUMAN	Glutathione S-transferase A1	45.05	38
sp|P16403|H12_HUMAN	Histone H1.2	25.35	29
sp|P30466|1B18_HUMAN	HLA class I histocompatibility antigen, B-18 alpha chain	35.36	16
sp|P01834|IGKC_HUMAN	Immunoglobulin kappa constant	82.24	52
sp|Q7RTV2|GSTA5_HUMAN	Glutathione S-transferase A5	18.92	15
sp|Q9P0M6|H2AW_HUMAN	Core histone macro-H2A.2	13.17	7
sp|P01764|HV323_HUMAN	Immunoglobulin heavy variable 3-23	31.62	8
sp|P42025|ACTY_HUMAN	Beta-centractin	22.61	5
sp|Q9NQH7|XPP3_HUMAN	Probable Xaa-Pro aminopeptidase 3	3.55	2
sp|P69891|HBG1_HUMAN	Hemoglobin subunit gamma-1	15.65	13
sp|P04430|KV116_HUMAN	Immunoglobulin kappa variable 1-16	29.06	2

sp|P01594|KV133_HUMAN	Immunoglobulin kappa variable 1-33	29.06	2
sp|O43447|PPIH_HUMAN	Peptidyl-prolyl cis-trans isomerase H	11.30	2
sp|P10114|RAP2A_HUMAN	Ras-related protein Rap-2a	6.01	1
sp|Q96DB5|RMD1_HUMAN	Regulator of microtubule dynamics protein 1	2.87	1
sp|P43243|MATR3_HUMAN	Matrin-3	2.13	1
sp|O43237|DC1L2_HUMAN	Cytoplasmic dynein 1 light intermediate chain 2	2.44	1
sp|Q6PIW4|FIGL1_HUMAN	Fidgetin-like protein 1	1.78	1
sp|Q9C0B5|ZDHC5_HUMAN	Palmitoyltransferase ZDHHC5	1.40	1
sp|Q86TJ2|TAD2B_HUMAN	Transcriptional adapter 2-beta	1.67	2
sp|P60981|DEST_HUMAN	Destrin	6.67	1
sp|O76062|ERG24_HUMAN	Delta(14)-sterol reductase	3.11	1
sp|Q15257|PTPA_HUMAN	Serine/threonine-protein phosphatase 2A activator	2.79	1
sp|O00233|PSMD9_HUMAN	26S proteasome non-ATPase regulatory subunit 9	5.38	2
sp|Q9UBB4|ATX10_HUMAN	Ataxin-10	2.32	1
sp|Q9H330|TM245_HUMAN	Transmembrane protein 245	1.43	1
sp|Q53GS9|SNUT2_HUMAN	U4/U6.U5 tri-snRNP-associated protein 2	2.83	1
sp|Q06136|KDSR_HUMAN	3-ketodihydrosphingosine reductase	3.31	1
sp|P08519|APOA_HUMAN	Apolipoprotein(a)	6.82	1
sp|Q9NRN7|ADPPT_HUMAN	L-aminoadipate-semialdehyde dehydrogenase-phosphopantetheinyl transferase	4.85	1
sp|Q9GZZ1|NAA50_HUMAN	N-alpha-acetyltransferase 50	6.51	1
sp|Q9BQ69|MACD1_HUMAN	O-acetyl-ADP-ribose deacetylase MACROD1	7.08	1
sp|Q86VR2|F134C_HUMAN	Protein FAM134C	3.22	1
sp|P49593|PPM1F_HUMAN	Protein phosphatase 1F	4.41	1
sp|A0A075B6P5|KV228_HUMAN	Immunoglobulin kappa variable 2-28	13.33	5
sp|P04156|PRIO_HUMAN	Major prion protein	4.35	1
sp|O75954|TSN9_HUMAN	Tetraspanin-9	10.88	3
sp|O75170|PP6R2_HUMAN	Serine/threonine-protein phosphatase 6 regulatory subunit 2	1.76	1
sp|Q9Y3C6|PPIL1_HUMAN	Peptidyl-prolyl cis-trans isomerase-like 1	6.63	1
sp|Q9Y237|PIN4_HUMAN	Peptidyl-prolyl cis-trans isomerase NIMA-interacting 4	9.16	1
sp|Q9ULC4|MCTS1_HUMAN	Malignant T-cell-amplified sequence 1	4.97	1
sp|Q9HAT2|SIAE_HUMAN	Sialate O-acetylesterase	2.29	1
sp|Q9H1C7|CYTM1_HUMAN	Cysteine-rich and transmembrane domain-containing protein 1	10.31	1
sp|Q9H0A8|COMD4_HUMAN	COMM domain-containing protein 4	9.55	1
sp|Q9BUN8|DERL1_HUMAN	Derlin-1	4.38	1
sp|Q96AZ6|ISG20_HUMAN	Interferon-stimulated gene 20 kDa protein	6.63	1
sp|Q92600|CNOT9_HUMAN	CCR4-NOT transcription complex subunit 9	4.01	1
sp|Q9P0S3|ORML1_HUMAN	ORM1-like protein 1	7.19	1
sp|Q8N0U8|VKORL_HUMAN	Vitamin K epoxide reductase complex subunit 1-like protein 1	6.25	1
sp|Q14011|CIRBP_HUMAN	Cold-inducible RNA-binding protein	19.77	2
sp|Q13642|FHL1_HUMAN	Four and a half LIM domains protein 1	5.88	2
sp|P82980|RET5_HUMAN	Retinol-binding protein 5	6.67	1
sp|P60903|S10AA_HUMAN	Protein S100-A10	17.53	1
sp|P42766|RL35_HUMAN	60S ribosomal protein L35	8.13	1
sp|P40429|RL13A_HUMAN	60S ribosomal protein L13a	5.42	1
sp|P20290|BTF3_HUMAN	Transcription factor BTF3	9.22	1
sp|P19075|TSN8_HUMAN	Tetraspanin-8	4.22	1
sp|P01308|INS_HUMAN	Insulin	6.36	1
sp|Q9Y5U8|MPC1_HUMAN	Mitochondrial pyruvate carrier 1	7.34	1
sp|Q9UNL2|SSRG_HUMAN	Translocon-associated protein subunit gamma	7.57	2
sp|Q9UII2|ATIF1_HUMAN	ATPase inhibitor, mitochondrial	7.55	1
sp|Q9UBQ0|VPS29_HUMAN	Vacuolar protein sorting-associated protein 29	4.40	1
sp|Q9NXA8|SIR5_HUMAN	NAD-dependent protein deacylase sirtuin-5, mitochondrial	5.16	1
sp|Q9NWV4|CA123_HUMAN	UPF0587 protein C1orf123	10.63	1
sp|Q9H299|SH3L3_HUMAN	SH3 domain-binding glutamic acid-rich-like protein 3	10.75	1
sp|Q9BWJ5|SF3B5_HUMAN	Splicing factor 3B subunit 5	15.12	1

sp|Q9BPX1|DHB14_HUMAN	17-beta-hydroxysteroid dehydrogenase 14	4.44	1
sp|Q99943|PLCA_HUMAN	1-acyl-sn-glycerol-3-phosphate acyltransferase alpha	3.53	1
sp|P63167|DYL1_HUMAN	Dynein light chain 1, cytoplasmic	12.36	1
sp|Q8TBQ9|KISHA_HUMAN	Protein kish-A	12.50	1
sp|Q8N6L1|KTAP2_HUMAN	Keratinocyte-associated protein 2	12.50	1
sp|Q7Z4R8|CF120_HUMAN	UPF0669 protein C6orf120	10.99	1
sp|P61956|SUMO2_HUMAN	Small ubiquitin-related modifier 2	12.63	1
sp|Q5BJF2|TMM97_HUMAN	Transmembrane protein 97	4.54	1
sp|Q16563|SYPL1_HUMAN	Synaptophysin-like protein 1	4.25	1
sp|Q15599|NHRF2_HUMAN	Na(+)/H(+) exchange regulatory cofactor NHE-RF2	2.97	1
sp|Q14657|LAGE3_HUMAN	EKC/KEOPS complex subunit LAGE3	16.08	1
sp|Q04941|PLP2_HUMAN	Proteolipid protein 2	8.55	1
sp|P67775|PP2AA_HUMAN	Serine/threonine-protein phosphatase 2A catalytic subunit alpha isoform	3.56	1
sp|P63208|SKP1_HUMAN	S-phase kinase-associated protein 1	7.36	1
sp|P62942|FKB1A_HUMAN	Peptidyl-prolyl cis-trans isomerase FKBP1A	12.04	2
sp|P62273|RS29_HUMAN	40S ribosomal protein S29	14.29	1
sp|P56134|ATPK_HUMAN	ATP synthase subunit f, mitochondrial	13.83	1
sp|P52434|RPAB3_HUMAN	DNA-directed RNA polymerases I, II, and III subunit RPABC3	8.67	1
sp|P50402|EMD_HUMAN	Emerin	3.94	1
sp|P38571|LICH_HUMAN	Lysosomal acid lipase/cholesteryl ester hydrolase	4.01	1
sp|P19397|CD53_HUMAN	Leukocyte surface antigen CD53	3.65	1
sp|P0DP06|HVD34_HUMAN	Immunoglobulin heavy variable 4-30-4	7.63	1
sp|P06730|IF4E_HUMAN	Eukaryotic translation initiation factor 4E	6.45	1
sp|P04216|THY1_HUMAN	Thy-1 membrane glycoprotein	6.21	1
sp|O95861|BPNT1_HUMAN	3'(2'),5'-bisphosphate nucleotidase 1	3.57	1
sp|O95562|SFT2B_HUMAN	Vesicle transport protein SFT2B	6.25	2
sp|O95168|NDUB4_HUMAN	NADH dehydrogenase [ubiquinone] 1 beta subcomplex subunit 4	7.75	1
sp|O14559|RHG33_HUMAN	Rho GTPase-activating protein 33	0.78	1
sp|O00168|PLM_HUMAN	Phospholemman	13.04	2
sp|A4D1S5|RAB19_HUMAN	Ras-related protein Rab-19	9.22	1
sp|A0A0C4DH68|KV224_HUMAN	Immunoglobulin kappa variable 2-24	10.83	1
sp|P00739|HPTR_HUMAN	Haptoglobin-related protein	39.37	84
sp|O14817|TSN4_HUMAN	Tetraspanin-4	2.94	1
sp|Q8WTS6|SETD7_HUMAN	Histone-lysine N-methyltransferase SETD7	2.46	1
sp|P17568|NDUB7_HUMAN	NADH dehydrogenase [ubiquinone] 1 beta subcomplex subunit 7	7.30	1
sp|Q8N6G6|ATL1_HUMAN	ADAMTS-like protein 1	0.96	2
sp|Q96MM6|HS12B_HUMAN	Heat shock 70 kDa protein 12B	1.46	1
sp|O95070|YIF1A_HUMAN	Protein YIF1A	5.12	1
sp|Q09161|NCBP1_HUMAN	Nuclear cap-binding protein subunit 1	1.39	1
sp|Q9NR31|SAR1A_HUMAN	GTP-binding protein SAR1a	11.62	2
sp|P49770|EI2BB_HUMAN	Translation initiation factor eIF-2B subunit beta	5.13	1
sp|Q8N142|PURA1_HUMAN	Adenylosuccinate synthetase isozyme 1	2.41	1
sp|Q9NTM9|CUTC_HUMAN	Copper homeostasis protein cutC homolog	10.99	2
sp|P19404|NDUV2_HUMAN	NADH dehydrogenase [ubiquinone] flavoprotein 2, mitochondrial	5.22	1
sp|Q00169|PIPNA_HUMAN	Phosphatidylinositol transfer protein alpha isoform	4.81	1
sp|Q14696|MESD_HUMAN	LDLR chaperone MESD	3.85	1
sp|Q14919|NC2A_HUMAN	Dr1-associated corepressor	10.24	2
sp|Q9UKV3|ACINU_HUMAN	Apoptotic chromatin condensation inducer in the nucleus	0.67	1
sp|P08311|CATG_HUMAN	Cathepsin G	3.14	1
sp|A0A075B6I0|LV861_HUMAN	Immunoglobulin lambda variable 8-61	7.38	1
sp|O00418|EF2K_HUMAN	Eukaryotic elongation factor 2 kinase	1.10	1
sp|A1A5D9|BICL2_HUMAN	BICD family-like cargo adapter 2	2.16	1
sp|Q9H0R4|HDHD2_HUMAN	Haloacid dehalogenase-like hydrolase domain-containing protein 2	3.09	1
sp|Q15800|MSMO1_HUMAN	Methylsterol monooxygenase 1	4.78	1
sp|Q29960|1C16_HUMAN	HLA class I histocompatibility antigen, Cw-16 alpha chain	28.96	16

sp|Q99567|NUP88_HUMAN	Nuclear pore complex protein Nup88	2.97	2
sp|Q8N8N7|PTGR2_HUMAN	Prostaglandin reductase 2	4.84	1
sp|P52788|SPSY_HUMAN	Spermine synthase	3.55	1
sp|Q8IUZ5|AT2L2_HUMAN	5-phosphohydroxy-L-lysine phospho-lyase	2.89	1
sp|O15155|BET1_HUMAN	BET1 homolog	15.25	1
sp|Q12874|SF3A3_HUMAN	Splicing factor 3A subunit 3	2.39	1
sp|P63000|RAC1_HUMAN	Ras-related C3 botulinum toxin substrate 1	7.29	1
sp|Q8NEV1|CSK23_HUMAN	Casein kinase II subunit alpha 3	4.60	1
sp|Q13885|TBB2A_HUMAN	Tubulin beta-2A chain	41.35	17
sp|Q96GS4|BORC6_HUMAN	BLOC-1-related complex subunit 6	2.80	1
sp|P02747|C1QC_HUMAN	Complement C1q subcomponent subunit C	12.24	2
sp|Q6UXB4|CLC4G_HUMAN	C-type lectin domain family 4 member G	3.41	1
sp|O95292|VAPB_HUMAN	Vesicle-associated membrane protein-associated protein B/C	15.23	3
sp|O14879|IFIT3_HUMAN	Interferon-induced protein with tetratricopeptide repeats 3	2.04	1
sp|P01036|CYTS_HUMAN	Cystatin-S	7.80	1
sp|P62829|RL23_HUMAN	60S ribosomal protein L23	16.43	2
sp|Q9NX76|CKLF6_HUMAN	CKLF-like MARVEL transmembrane domain-containing protein 6	11.48	2
sp|Q8WWI1|LMO7_HUMAN	LIM domain only protein 7	1.31	2
sp|Q14118|DAG1_HUMAN	Dystroglycan	4.47	3
sp|P24390|ERD21_HUMAN	ER lumen protein-retaining receptor 1	5.19	1
sp|P35611|ADDA_HUMAN	Alpha-adducin	5.16	3
sp|O95817|BAG3_HUMAN	BAG family molecular chaperone regulator 3	5.22	2
sp|O95747|OXSR1_HUMAN	Serine/threonine-protein kinase OSR1	1.71	1
sp|Q9HCE1|MOV10_HUMAN	Putative helicase MOV-10	1.00	1
sp|Q92544|TM9S4_HUMAN	Transmembrane 9 superfamily member 4	1.40	1
sp|O60232|SSA27_HUMAN	Sjoegren syndrome/scleroderma autoantigen 1	11.06	3
sp|Q96DZ1|ERLEC_HUMAN	Endoplasmic reticulum lectin 1	2.69	1
sp|Q9NX62|IMPA3_HUMAN	Inositol monophosphatase 3	10.86	2
sp|Q9BXJ8|T120A_HUMAN	Transmembrane protein 120A	2.62	1
sp|Q969U7|PSMG2_HUMAN	Proteasome assembly chaperone 2	7.95	2
sp|Q7Z478|DHX29_HUMAN	ATP-dependent RNA helicase DHX29	0.66	1
sp|Q14978|NOLC1_HUMAN	Nucleolar and coiled-body phosphoprotein 1	1.43	1
sp|Q9GZZ9|UBA5_HUMAN	Ubiquitin-like modifier-activating enzyme 5	2.23	1
sp|Q9UKK3|PARP4_HUMAN	Poly [ADP-ribose] polymerase 4	1.22	2
sp|P10153|RNAS2_HUMAN	Non-secretory ribonuclease	9.32	1
sp|P14854|CX6B1_HUMAN	Cytochrome c oxidase subunit 6B1	9.30	1
sp|Q14166|TTL12_HUMAN	Tubulin--tyrosine ligase-like protein 12	1.55	1
sp|Q96IJ6|GMPPA_HUMAN	Mannose-1-phosphate guanyltransferase alpha	3.81	1
sp|P35052|GPC1_HUMAN	Glypican-1	1.97	1
sp|P06703|S10A6_HUMAN	Protein S100-A6	8.89	2
sp|Q96QA5|GSDMA_HUMAN	Gasdermin-A	2.02	1
sp|P21283|VATC1_HUMAN	V-type proton ATPase subunit C 1	4.71	2
sp|Q9GIY3|2B1E_HUMAN	HLA class II histocompatibility antigen, DRB1-14 beta chain	16.92	6
sp|Q9GZP4|PITH1_HUMAN	PITH domain-containing protein 1	9.48	2
sp|Q8NBK3|SUMF1_HUMAN	Sulfatase-modifying factor 1	3.21	1
sp|Q6GMV3|PTRD1_HUMAN	Putative peptidyl-tRNA hydrolase PTRHD1	8.57	1
sp|P15814|IGLL1_HUMAN	Immunoglobulin lambda-like polypeptide 1	10.80	2
sp|P48507|GSH0_HUMAN	Glutamate--cysteine ligase regulatory subunit	4.74	1
sp|O15484|CAN5_HUMAN	Calpain-5	1.56	1
sp|Q9P016|THYN1_HUMAN	Thymocyte nuclear protein 1	6.67	1
sp|Q9BQA1|MEP50_HUMAN	Methylosome protein 50	4.39	1
sp|P30047|GFRP_HUMAN	GTP cyclohydrolase 1 feedback regulatory protein	19.05	1
sp|Q9UNU6|CP8B1_HUMAN	7-alpha-hydroxycholest-4-en-3-one 12-alpha-hydroxylase	6.39	2
sp|P09132|SRP19_HUMAN	Signal recognition particle 19 kDa protein	10.42	1
sp|P10301|RRAS_HUMAN	Ras-related protein R-Ras	5.05	1
sp|P06213|INSR_HUMAN	Insulin receptor	1.52	2

sp|Q92890|UFD1_HUMAN	Ubiquitin recognition factor in ER-associated degradation protein 1	3.91	1
sp|Q9P2X0|DPM3_HUMAN	Dolichol-phosphate mannosyltransferase subunit 3	10.87	1
sp|O95816|BAG2_HUMAN	BAG family molecular chaperone regulator 2	4.26	1
sp|P46779|RL28_HUMAN	60S ribosomal protein L28	8.03	1
sp|Q9NUQ9|FA49B_HUMAN	Protein FAM49B	6.79	2
sp|P02814|SMR3B_HUMAN	Submaxillary gland androgen-regulated protein 3B	44.30	3
sp|Q92878|RAD50_HUMAN	DNA repair protein RAD50	1.60	2
sp|Q96B45|BORC7_HUMAN	BLOC-1-related complex subunit 7	9.52	1
sp|Q9NQG1|MANBL_HUMAN	Protein MANBAL	14.12	1
sp|Q92520|FAM3C_HUMAN	Protein FAM3C	5.29	1
sp|Q9Y6K0|CEPT1_HUMAN	Choline/ethanolaminephosphotransferase 1	2.64	1
sp|P49908|SEPP1_HUMAN	Selenoprotein P	2.62	1
sp|P35080|PROF2_HUMAN	Profilin-2	10.00	1
sp|Q8WWQ8|STAB2_HUMAN	Stabilin-2	0.35	1
sp|Q14847|LASP1_HUMAN	LIM and SH3 domain protein 1	14.94	3
sp|Q96RQ3|MCCA_HUMAN	Methylcrotonoyl-CoA carboxylase subunit alpha, mitochondrial	1.52	1
sp|P48059|LIMS1_HUMAN	LIM and senescent cell antigen-like-containing domain protein 1	3.69	1
sp|Q9P2R7|SUCB1_HUMAN	Succinate--CoA ligase [ADP-forming] subunit beta, mitochondrial	7.56	2
sp|Q02487|DSC2_HUMAN	Desmocollin-2	1.11	1
sp|P61599|NAA20_HUMAN	N-alpha-acetyltransferase 20	8.43	1
sp|Q9NTG7|SIR3_HUMAN	NAD-dependent protein deacetylase sirtuin-3, mitochondrial	2.76	1
sp|P31150|GDIA_HUMAN	Rab GDP dissociation inhibitor alpha	19.91	11
sp|P05981|HEPS_HUMAN	Serine protease hepsin	4.08	1
sp|P27694|RFA1_HUMAN	Replication protein A 70 kDa DNA-binding subunit	2.60	1
sp|P01709|LV208_HUMAN	Immunoglobulin lambda variable 2-8	6.78	1
sp|Q86VX2|COMD7_HUMAN	COMM domain-containing protein 7	5.50	1
sp|P20851|C4BPB_HUMAN	C4b-binding protein beta chain	7.54	2
sp|Q9UHY7|ENOPH_HUMAN	Enolase-phosphatase E1	4.60	1
sp|A0AVT1|UBA6_HUMAN	Ubiquitin-like modifier-activating enzyme 6	2.28	2
sp|Q15276|RABE1_HUMAN	Rab GTPase-binding effector protein 1	2.20	1
sp|P00813|ADA_HUMAN	Adenosine deaminase	6.34	2
sp|P30405|PPIF_HUMAN	Peptidyl-prolyl cis-trans isomerase F, mitochondrial	4.35	1
sp|P51608|MECP2_HUMAN	Methyl-CpG-binding protein 2	2.88	1
sp|Q6ZVZ8|ASB18_HUMAN	Ankyrin repeat and SOCS box protein 18	4.94	3
sp|P38919|IF4A3_HUMAN	Eukaryotic initiation factor 4A-III	6.57	2
sp|O75907|DGAT1_HUMAN	Diacylglycerol O-acyltransferase 1	3.28	1
sp|Q96DA0|ZG16B_HUMAN	Zymogen granule protein 16 homolog B	4.33	1
sp|Q9H0V9|LMA2L_HUMAN	VIP36-like protein	9.77	2
sp|Q15428|SF3A2_HUMAN	Splicing factor 3A subunit 2	6.03	2
sp|Q9UBE0|SAE1_HUMAN	SUMO-activating enzyme subunit 1	3.18	1
sp|P26368|U2AF2_HUMAN	Splicing factor U2AF 65 kDa subunit	2.11	1
sp|P03950|ANGI_HUMAN	Angiogenin	10.88	1
sp|Q8N139|ABCA6_HUMAN	ATP-binding cassette sub-family A member 6	0.49	1
sp|P25445|TNR6_HUMAN	Tumor necrosis factor receptor superfamily member 6	4.78	1
sp|P13284|GILT_HUMAN	Gamma-interferon-inducible lysosomal thiol reductase	10.00	3
sp|O00186|STXB3_HUMAN	Syntaxin-binding protein 3	1.52	1
sp|Q4KMQ2|ANO6_HUMAN	Anoctamin-6	0.99	1
sp|Q9BZE9|ASPC1_HUMAN	Tether containing UBX domain for GLUT4	2.89	1
sp|Q4G0F5|VP26B_HUMAN	Vacuolar protein sorting-associated protein 26B	3.27	1
sp|P18621|RL17_HUMAN	60S ribosomal protein L17	5.43	1
sp|Q9UMX0|UBQL1_HUMAN	Ubiquilin-1	7.47	3
sp|Q92466|DDB2_HUMAN	DNA damage-binding protein 2	4.68	2
sp|Q9BTY2|FUCO2_HUMAN	Plasma alpha-L-fucosidase	2.36	1
sp|Q9Y2D2|S35A3_HUMAN	UDP-N-acetylglucosamine transporter	4.61	1
sp|Q13790|APOF_HUMAN	Apolipoprotein F	4.29	1
sp|Q99747|SNAG_HUMAN	Gamma-soluble NSF attachment protein	9.94	3

sp|Q9H078|CLPB_HUMAN	Caseinolytic peptidase B protein homolog	4.24	2
sp|Q9UBT2|SAE2_HUMAN	SUMO-activating enzyme subunit 2	1.41	1
sp|Q96GX9|MTNB_HUMAN	Methylthioribulose-1-phosphate dehydratase	4.96	1
sp|P27986|P85A_HUMAN	Phosphatidylinositol 3-kinase regulatory subunit alpha	1.52	1
sp|P19388|RPAB1_HUMAN	DNA-directed RNA polymerases I, II, and III subunit RPABC1	4.29	1
sp|P00748|FA12_HUMAN	Coagulation factor XII	1.30	1
sp|O94985|CSTN1_HUMAN	Calsyntenin-1	2.45	2
sp|Q6P179|ERAP2_HUMAN	Endoplasmic reticulum aminopeptidase 2	0.94	1
sp|P42892|ECE1_HUMAN	Endothelin-converting enzyme 1	2.34	1
sp|Q99615|DNJC7_HUMAN	DnaJ homolog subfamily C member 7	2.23	1
sp|Q92974|ARHG2_HUMAN	Rho guanine nucleotide exchange factor 2	1.01	1
sp|Q13509|TBB3_HUMAN	Tubulin beta-3 chain	33.33	16
sp|Q8NBF2|NHLC2_HUMAN	NHL repeat-containing protein 2	1.24	1
sp|Q8TF65|GIPC2_HUMAN	PDZ domain-containing protein GIPC2	4.76	1
sp|Q9Y646|CBPQ_HUMAN	Carboxypeptidase Q	4.24	2
sp|Q16576|RBBP7_HUMAN	Histone-binding protein RBBP7	9.41	4
sp|Q9NZ01|TECR_HUMAN	Very-long-chain enoyl-CoA reductase	7.79	2
sp|Q10471|GALT2_HUMAN	Polypeptide N-acetylgalactosaminyltransferase 2	5.43	2

Supplementary Table 2

Accession	Protein	Fold -Change (Approach 1)	p-value (Approach 1)	Fold change (Approach 2)	p-value (Approach 2)
P55072	Transitional endoplasmic reticulum ATPase	-34.267	0.006	-6.061	0.006
P35573	Glycogen debranching enzyme	-9.912	0.002	-3.295	0.038
P22310	UDP-glucuronosyltransferase 1-4	-9.897	0.005	-4.903	0.008
P36537	UDP-glucuronosyltransferase 2B10	-9.824	0.005	-7.726	0.026
P05166	Propionyl-CoA carboxylase beta chain, mitochondrial	-9.265	0.020	-3.334	0.013
P16662	UDP-glucuronosyltransferase 2B7	-9.122	0.003	-5.666	0.014
P61158	Actin-related protein 3	-7.974	0.000	-2.746	0.009
P22760	Arylacetamide deacetylase	-7.604	0.003	-17.457	0.012
P31327	Carbamoyl-phosphate synthase [ammonia], mitochondrial	-6.762	0.000	-7.465	0.001
P19224	UDP-glucuronosyltransferase 1-6	-6.538	0.004	-6.649	0.009
P30101	Protein disulfide-isomerase A3	-4.502	0.010	-5.444	0.002
O75891	Cytosolic 10-formyltetrahydrofolate dehydrogenase	-4.071	0.019	-4.197	0.017
P00966	Argininosuccinate synthase	-3.703	0.003	-4.426	0.003
P21549	Serine--pyruvate aminotransferase	-3.681	0.009	-2.559	0.030
O15145	Actin-related protein 2/3 complex subunit 3	-3.599	0.030	-13.529	0.003
P42765	3-ketoacyl-CoA thiolase, mitochondrial	-3.156	0.000	-3.635	0.002
P80404	4-aminobutyrate aminotransferase, mitochondrial	-2.804	0.038	-4.325	0.036
P09417	Dihydropteridine reductase	-2.767	0.018	-3.142	0.022
P00558	Phosphoglycerate kinase 1	-2.761	0.004	-4.002	0.005
O95954	Formimidoyltransferase-cyclodeaminase	-2.729	0.025	-2.961	0.044
Q00796	Sorbitol dehydrogenase	-2.633	0.001	-4.314	0.000
P07099	Epoxide hydrolase 1	-2.239	0.043	-4.280	0.008
P54868	Hydroxymethylglutaryl-CoA synthase, mitochondrial	-2.150	0.021	-4.227	0.001
O75874	Isocitrate dehydrogenase [NADP] cytoplasmic	-2.132	0.018	-2.643	0.017
P16152	Carbonyl reductase [NADPH] 1	-2.081	0.031	-2.465	0.013
P08319	Alcohol dehydrogenase 4	-1.916	0.002	-3.627	0.004
P36871	Phosphoglucomutase-1	-1.837	0.044	-4.127	0.005
Q13510	Acid ceramidase	3.716	0.022	3.402	0.034
P30049	ATP synthase subunit delta, mitochondrial	4.317	0.031	9.208	0.040
P06756	Integrin alpha-V	4.404	0.042	4.753	0.034
P61981	14-3-3 protein gamma	4.406	0.010	4.539	0.045
P18206	Vinculin	6.068	0.019	10.292	0.017
P13716	Delta-aminolevulinic acid dehydratase	7.135	0.048	5.950	0.013
P68871	Hemoglobin subunit beta	16.006	0.004	5.456	0.012
P13796	Plastin-2	29.612	0.000	11.668	0.013

Supplementary Table 3

A.

Accession	Protein	Fold-Change (Approach 1)	p-value (Approach 1)	Fold-Change (Approach 2)	p-value (Approach 2)
P18669	Phosphoglycerate mutase 1	-9.617	0.000	-3.309	0.023
P11766	Alcohol dehydrogenase class-3	-6.561	0.001	-4.624	0.021
P34897	Serine hydroxymethyltransferase, mitochondrial	-5.416	0.020	-7.888	0.022

B.

Accession	Protein	Fold-Change (Approach 1)	p-value (Approach 1)	Fold-Change (Approach 2)	p-value (Approach 2)
P02751	Fibronectin	-29.674	0.003	-5.269	0.033
P04114	Apolipoprotein B-100	-13.729	0.033	-5.686	0.012
Q08380	Galectin-3-binding protein	-12.588	0.007	-4.863	0.044
P02743	Serum amyloid P-component	-11.927	0.010	-9.991	0.040
P09871	Complement C1s subcomponent	-11.464	0.002	-3.829	0.046
P10909	Clusterin	-10.348	0.001	-2.953	0.018
P00736	Complement C1r subcomponent	-9.763	0.004	-3.705	0.046
P18669	Phosphoglycerate mutase 1	-5.734	0.002	-2.749	0.046
P45954	Short/branched chain specific acyl-CoA dehydrogenase, mitochondrial	-4.281	0.016	-2.084	0.035
P13639	Elongation factor 2	-2.316	0.020	-1.765	0.032

C.

Accession	Protein	Fold-Change (Approach 1)	p-value (Approach 1)	Fold-Change (Approach 2)	p-value (Approach 2)
P34897	Serine hydroxymethyltransferase, mitochondrial	-6.271	0.013	-10.353	0.012
P02749	Beta-2-glycoprotein 1	-5.697	0.001	-20.971	0.009
P04075	Fructose-bisphosphate aldolase A	-3.958	0.026	-2.315	0.031
Q14117	Dihydropyrimidinase	-3.663	0.042	2.592	0.038
P52907	F-actin-capping protein subunit alpha-1	-3.313	0.022	-3.281	0.020
P13639	Elongation factor 2	-3.162	0.004	-2.221	0.007
Q00796	Sorbitol dehydrogenase	-2.813	0.003	-1.923	0.042

D.

Accession	Protein	Fold-Change (Approach 1)	p-value (Approach 1)	Fold-Change (Approach 2)	p-value (Approach 2)
P55072	Transitional endoplasmic reticulum ATPase	-118.363	0.004	-9.011	0.008
P05166	Propionyl-CoA carboxylase beta chain, mitochondrial	-21.912	0.013	-4.647	0.013
P61158	Actin-related protein 3	-21.849	0.000	-3.912	0.006
P31327	Carbamoyl-phosphate synthase [ammonia], mitochondrial	-13.944	0.000	-9.629	0.002
P21549	Serine--pyruvate aminotransferase	-12.637	0.001	-3.485	0.024
P25787	Proteasome subunit alpha type-2	-11.459	0.047	-4.488	0.000
O75891	Cytosolic 10-formyltetrahydrofolate dehydrogenase	-11.302	0.004	-6.735	0.013
P04114	Apolipoprotein B-100	-10.841	0.047	-5.865	0.011
P36537	UDP-glucuronosyltransferase 2B10	-10.734	0.013	-11.767	0.032
P16662	UDP-glucuronosyltransferase 2B7	-10.513	0.008	-5.035	0.049
P42765	3-ketoacyl-CoA thiolase, mitochondrial	-8.624	0.000	-4.513	0.003
P00966	Argininosuccinate synthase	-7.680	0.001	-5.409	0.005
Q96DG6	Carboxymethylenebutenolidase homolog	-7.186	0.024	-3.104	0.025
P19224	UDP-glucuronosyltransferase 1-6	-6.977	0.012	-5.458	0.042
P59998	Actin-related protein 2/3 complex subunit 4	-6.956	0.039	-9.438	0.029
P30101	Protein disulfide-isomerase A3	-6.811	0.010	-4.781	0.011
P80404	4-aminobutyrate aminotransferase, mitochondrial	-6.793	0.007	-8.720	0.019
Q00796	Sorbitol dehydrogenase	-6.258	0.000	-5.275	0.000
P00558	Phosphoglycerate kinase 1	-5.641	0.001	-4.108	0.014
O15145	Actin-related protein 2/3 complex subunit 3	-5.444	0.025	-33.334	0.002
P13639	Elongation factor 2	-5.403	0.000	-1.897	0.019
P09417	Dihydropteridine reductase	-5.345	0.005	-4.726	0.016
P16152	Carbonyl reductase [NADPH] 1	-4.551	0.003	-2.744	0.023
P54868	Hydroxymethylglutaryl-CoA synthase, mitochondrial	-3.947	0.004	-4.906	0.003
P09871	Complement C1s subcomponent	-3.886	0.032	-3.726	0.050
P36871	Phosphoglucomutase-1	-3.748	0.003	-4.604	0.011
P51659	Peroxisomal multifunctional enzyme type 2	-3.569	0.039	-6.938	0.008
P08319	Alcohol dehydrogenase 4	-3.431	0.000	-3.552	0.016
P07099	Epoxide hydrolase 1	-3.191	0.026	-3.713	0.037
P10909	Clusterin	-2.805	0.042	-3.052	0.016
P24752	Acetyl-CoA acetyltransferase, mitochondrial	-2.785	0.007	-3.297	0.007
P05091	Aldehyde dehydrogenase, mitochondrial	-2.668	0.045	-5.822	0.001
P68871	Hemoglobin subunit beta	22.068	0.008	6.160	0.025
